# Complete Assignments of ^1^H and ^13^C NMR Chemical Shift Changes Observed upon Protection of Hydroxy Group in Borneol and Isoborneol and Their DFT Verification

**DOI:** 10.3390/molecules30030597

**Published:** 2025-01-28

**Authors:** Baohe Lyu, Yoshikazu Hiraga, Ryukichi Takagi, Satomi Niwayama

**Affiliations:** 1 Graduate School of Science and Technology, Hiroshima Institute of Technology, 2-1-1 Miyake, Saeki-ku, Hiroshima 731-5193, Japan; 2 Department of Chemistry, Graduate School of Advanced Science and Engineering, Hiroshima University, 1-3-1 Kagamiyama, Higashi-Hiroshima 739-8526, Japan; 3 Graduate School of Engineering, Muroran Institute of Technology, 27-1 Mizumoto-cho, Muroran 050-8585, Japan

**Keywords:** NMR assignment, chemical shift change, DFT calculations, iso-chemical shielding surface, anisotropic effect

## Abstract

Complete assignments of the ^1^H and ^13^C NMR chemical shifts for the monoterpenes, borneol **1a** and isoborneol **2a**, as well as their derivatives (**1b**–**1g** and **2b**–**2g**), in which the secondary hydroxy group is protected with various protecting groups, have been made in various solvents. Upon protection of the hydroxy groups in **1a** and **2a**, many protons and carbons within the bicyclic ring exhibited downfield or upfield shifts in their chemical shift values, facilitating the unambiguous assignments of these protons and carbons. These chemical shift values also showed excellent correlations with those obtained from density functional theory (DFT) calculations. Furthermore, the anisotropic effect of the benzene ring was estimated by the analysis of the iso-chemical shielding surface (ICSS) resulting from substituents introduced to the hydroxyl groups of **1a** and **2a**.

## 1. Introduction

**Scheme 1:**
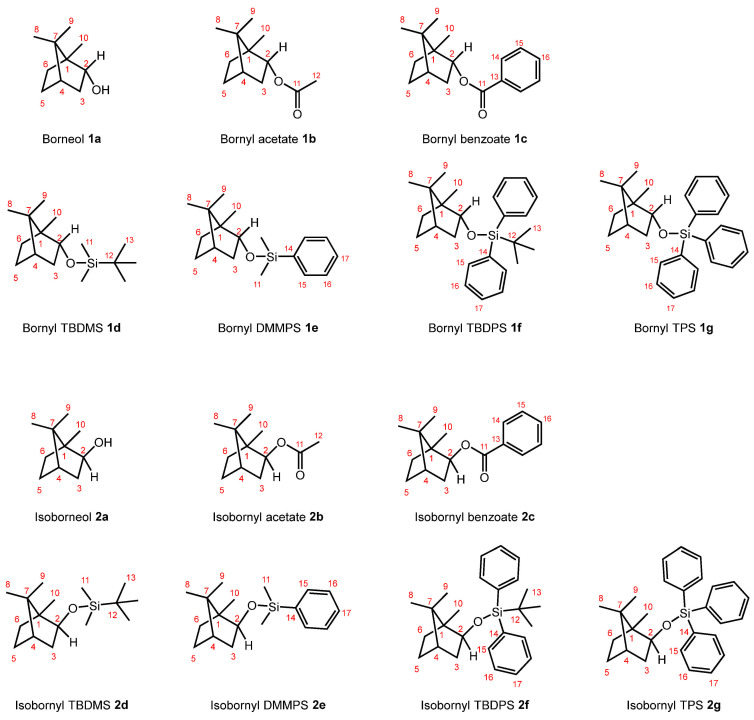
Borneol **1a**, isoborneol **2a**, and their derivatives **1b**–**1g** and **2b**–**2g**.

Borneol **1a** and isoborneol **2a** are chiral monoterpenoids abundant in nature and constitute components of plant essential oils (Scheme 1) [1,2,3,4,5,6,7,8]. These natural products consist of a bicyclic ring, and are epimers of each other in which the stereochemistry of the hydroxy group is different. Both of them have been utilized in a wide range of products such as pharmaceuticals, insect repellant, fragrance, and chiral ligands since many decades ago [9,10,11,12]. Therefore, their spectroscopic properties such as ^1^H and ^13^C NMR spectroscopic behaviors have been actively investigated for the last few decades [13,14,15,16,17,18,19].

NMR spectroscopy plays a key role for structure determination as well as obtaining information about various chemical environments of organic compounds in general, even distinguishing subtle structural differences. Although high resolution NMR spectroscopy made magnificent advancements recently, the complete assignments of ^1^H NMR and ^13^C NMR chemical shifts for these compounds have still been rather challenging, for isobrorneol **2a** in particular, because of some overlapping peaks. In spite of the difficulties, we previously reported our complete assignments of ^1^H NMR chemical shifts of both borneol **1a** and isoborneol **2a** by protecting each hydroxy group with various protecting groups [20,21]. We found high-frequency shifts or low-frequency shifts (hereafter referred to as downfield shifts and upfield shifts, meaning deshielding and shielding, respectively) for many protons, including those distant from the protecting groups that have phenyl groups. This is likely to be attributed to anisotropic effects, facilitating the complete assignments.

Here, based on quantum chemical calculations, we investigated the change of the chemical shift values observed upon the protection of the hydroxy groups in **1a** and **2a** with various carbonyl-containing protecting groups or with silyl-protecting groups having a various number of phenyl rings. We optimized the most stable structures of **1a**, **2a**, and their derivatives (**1b**–**1g** and **2b**–**2g**), all of which were adopted in the experiments, using density functional theory (DFT) calculations with the Gaussian 09 program [22]. We further predicted their ^1^H NMR and ^13^C NMR chemical shifts based on these structures and compared them with the experimental ^1^H NMR and ^13^C NMR chemical shifts. Furthermore, for quantitative investigation of the anisotropic effects arising from the carbonyl group or the phenyl group, we calculated the iso-chemical shielding surface (ICSS) [23], which is a real-space function closely related to the nucleus-independent chemical shift (NICS) [24,25,26,27,28,29] and can be applied to the evaluation of the aromaticity of cyclic molecules or local rings [30,31,32,33,34,35,36,37,38,39,40,41]. The ICSS method can clearly reveal the extent of magnetic shielding and deshielding effects at different regions due to the delocalized electrons, and therefore we have been able to explain the origin of the chemical shift changes observed upon the protection of each hydroxy group with the above protecting groups.

## 2. Results and Discussion

### 2.1. The Correlation Between the Experimentally Obtained and DFT-Calculated ^1^H and ^13^C NMR Chemical Shifts of Borneol ***1a***, Isoborneol ***2a***, and Their Derivatives ***1b***–***1g*** and ***2b***–***2g***

The chemical shift changes in ^1^H NMR and ^13^C NMR upon the introduction of acyl (acetyl and benzoyl) or silyl protective groups to borneol and isoborneol were investigated. The NMR solvents used were CDCl_3_, C_6_D_6_, and CD_3_OD, and the differences due to the solvent effects were also examined. However, compounds **1d**–**1g** and **2d**–**2g** with the silyl groups were insoluble in CD_3_OD, and therefore, comparisons were made using CDCl_3_ and C_6_D_6_.

**Figure 1:**
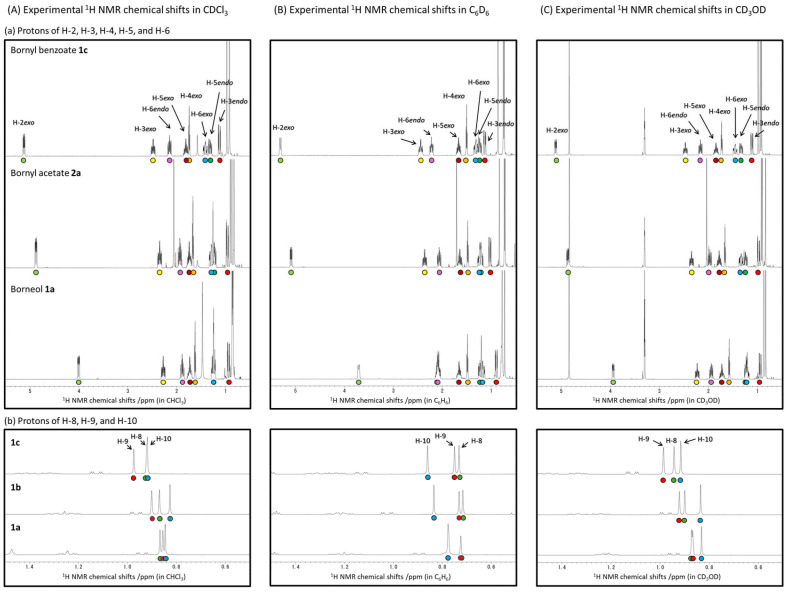
^1^H NMR chemical shift changes of borneol **1a** and its derivatives **1b**–**1c** in different solvents (CDCl_3_, C_6_D_6_ or CD_3_OD).

**Figure 2:**
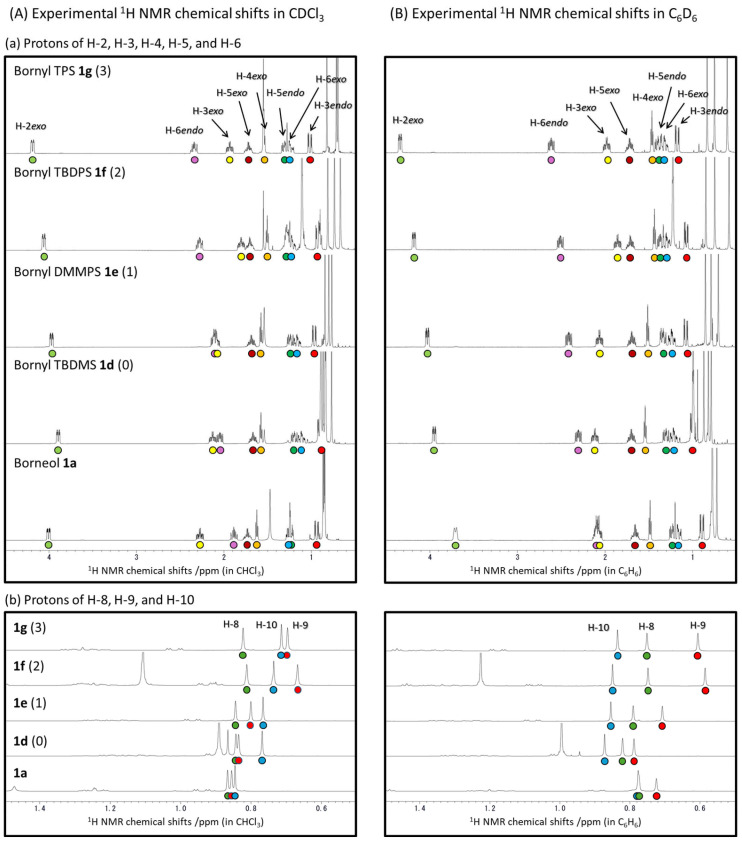
^1^H NMR chemical shift changes of borneol **1a** and its derivatives **1d**–**1g** in different solvents (CDCl_3_ or C_6_D_6_). The number in parentheses under the compound number indicates the number of phenyl groups.

The assignments in the ^1^H NMR and ^13^C NMR spectra upon the introduction of an acetyl group or a benzoyl group to the hydroxyl group of borneol and isoborneol are shown in Tables S1–S4, and the assignments in the spectra upon the introduction of silyl groups with varying numbers of phenyl groups are shown in Tables S5–S8 [20,21]. Furthermore, the ^1^H NMR chemical shift changes caused by the difference in electron-withdrawing properties between acetyl and benzoyl groups are shown in Figure 1 for borneol **1a** and its derivatives **1b**–**1c** and in Figure S1 for isoborneol **2a** and its derivatives **2b**–**1c**. The chemical shift changes caused by the number of phenyl groups in silyl groups with varying numbers of phenyl groups are shown in Figure 2 for borneol derivatives **1d**–**1g** and in Figure S2 for isoborneol derivatives **2d**–**2g**.

The anisotropy and shielding effects of the phenyl group will be discussed in a later chapter. It was found that the introduction of an acetyl group induces downfield shifts for most protons in borneol derivatives and isoborneol derivatives, as in Figure 1 and Figure S1. Additional downfield shifts were also found upon the introduction of a benzoyl group. Additionally, regarding the solvent effects caused by the NMR solvent, similar chemical shifts were observed in CDCl_3_ and CD_3_OD. However, in C_6_D_6_, due to the anisotropic effects of the solvent [42], the methyl groups exhibited chemical shifts and chemical shift changes that were different from those in the other solvents, as shown in Figure 1 and Figure S1.

On the other hand, as shown in Figure 2 and Figure S2, the chemical shift changes observed upon introducing silyl protecting groups to borneol **1a** and isoborneol **2a** showed minimal differences regardless of the solvent. However, unlike the case of acyl groups, as the number of phenyl groups in the silyl protecting groups increases, not only did the protons of the compounds exhibit downfield shifts in the ^1^H NMR chemical shifts, but protons showing upfield shifts, such as the methyl protons at C-8, were also observed, as shown in Figure 2 and Figure S2. Therefore, we used quantum chemical calculations to investigate the changes in the chemical shifts of ^1^H NMR and ^13^C NMR when acyl groups or silyl groups were introduced into borneol **1a** and isoborneol **1b**.

The most stable structure of each compound was determined with the use of the Gaussian 09 program with DFT methods at the B3LYP/6-311+G(2d,p), ωB97XD/6-311+G(2d,p), and *m*PW1PW91/6-311+G(2d,p) levels [43,44,45,46,47], and GaussView 5 was utilized for the creation of the input files and analysis of the results [22]. The calculations of the NMR chemical shifts were performed with the use of the gauge, including the atomic orbital (GIAO) technique in combination with the *m*PW1PW91/6-311+G(2d,p) level [48,49,50,51,52]. In order to verify the solvent effects and validities of the chemical shifts of each compound in each solvent, we compared the chemical shift values obtained by the DFT calculations (GIAO/*m*PW1PW91/6-311+G(2d,p) level of theory with IEFPCM in the gas phase and several solvents) in each solvent and the spectroscopically observed values in each solvent [53,54,55,56,57,58,59,60].

The linear correlations between the experimental and calculated NMR chemical shifts for the bornyl and isobornyl skeletons were investigated. The results are shown in Figures S3 and S4 for borneol **1a** and its derivatives **1b** and **1c,** as well as in Figures S5 and S6 for isoborneol **2a** and its derivatives **2b** and **2c**.

As for ^13^C NMR chemical shifts, a good correlation was observed between the calculated values in the gas phase and the observed values in each solvent (CDCl_3_, C_6_D_6_, or CD_3_OD) for both borneol **1a** and isoborneol **2a**, as well as for their derivatives **1b**, **1c**, **2b**, and **2c,** as shown in Figures S3 and S5. However, for the calculated values of ^1^H NMR chemical shifts in the gas phase, some variation was observed in the high-field region depending on the measurement solvent. Therefore, the correlations between the calculated values of ^1^H NMR chemical shift with IEFPCM in each solvent and the corresponding observed values were examined. As shown in Figures S4 and S6, far better correlations were found in these solvents than in the gas phase. In particular, good correlations were observed in chloroform and in methanol for all these compounds, especially for isoborneol **2a** and its derivative **2c** (Figure S6). As a result, excellent correlations were found between the chemical shift values obtained by the calculations and those observed by ^1^H and ^13^C NMR spectroscopy in each solvent. In general, it is considered difficult to estimate the extent of anisotropic effects by theoretical calculations, but these results are likely to indicate that quantum chemical calculations at the GIAO/*m*PW1PW91/6-311+G(2d,p) level with IEFPCM in solvent (CDCl_3_, C_6_D_6_, or CD_3_OD) can properly estimate the ^1^H and ^13^C NMR chemical shift values for these uniquely strained bicyclic molecules.

Next, in order to verify the changes in the chemical shifts observed in the ^1^H and ^13^C NMR spectra of borneol **1a** and isoborneol **2a** and their derivatives **1d**–**1g** and **2d**–**2g** protected with silyl groups having various numbers of phenyl groups in CDCl_3_ or C_6_D_6_ solutions, we compared the experimental ^1^H NMR and ^13^C NMR chemical shifts in these solutions with those estimated by quantum chemical calculations based on the most stable structures in the gas phase.

Figures S7 and S8 show the correlation between the experimental ^1^H and ^13^C NMR chemical shifts for the bornyl skeleton in CDCl_3_ and C_6_D_6_ solutions and the calculated ^1^H and ^13^C NMR chemical shifts in the gas phase for borneol **1a** and its silyl-protected derivatives **1d**–**1g**. They were insoluble in CD_3_OD, as noted previously. Very good correlations were observed between the experimental ^1^H and ^13^C NMR chemical shifts in CDCl_3_ or C_6_D_6_ and the calculated ^1^H and ^13^C NMR chemical shifts, respectively, for the same set of compounds. These results suggest that the expected ^13^C NMR chemical shift values calculated from the most stable structures reflect the experimentally observed values even after the introduction of the silyl group and the increase in the number of phenyl groups within the silyl groups. It is also suggested that the most stable structures are all valid, and the ^1^H NMR and ^13^C NMR shielding calculations derived from these most stable structures are also proper. Furthermore, as shown in Figure S8, concerning the ^1^H NMR chemical shift correlation, a slightly better linear correlation between the calculated values and the observed values was observed in C_6_D_6_ than in CDCl_3_. Therefore, we believe that the most stable structures in this case properly reflect the experimentally observed anisotropic effects from the phenyl groups in C_6_D_6_ solution [61,62,63,64,65,66].

Figures S9 and S10 show the correlation between the experimental ^1^H and ^13^C NMR chemical shifts for the isobornyl skeleton in CDCl_3_ and C_6_D_6_ solutions, for isoborneol **2a** and its silyl-protected derivatives **2d**–**2g,** and the calculated ^1^H and ^13^C NMR chemical shifts in the gas phase. As with the results for borneol **1a** and its derivatives **1d**–**1g**, very good correlations were found between the experimental ^13^C NMR chemical shifts and the calculated ^13^C NMR chemical shifts for isoborneol **2a** and its derivatives **2d**–**2g** in CDCl_3_ or C_6_D_6_. Notably, although isoborneol **2a** possesses a very crowded *exo* hydroxy group at C-2, good correlations between the experimental chemical shifts and calculated ones were still detected even after the introduction of the silyl groups, verifying the most stable conformations.

From the above results, the experimental values of ^1^H NMR chemical shifts in the gas phase, some variation was observed in the high-field region depending on the measurement solvent, as shown in Figures S7 and S9. Therefore, the correlations between the calculated values of ^1^H NMR chemical shift with IEFPCM in each solvent and the corresponding observed values were examined. As shown in Figures S8 and S10, far better correlations were found in these solvents than in the gas phase.

### 2.2. Relationship Between the Changes in ^1^H NMR Chemical Shifts of Borneol ***1a***, Isoborneol ***2a***, and Their Derivatives Containing an Acetyl Group or a Benzoyl Group ***1b***–***1c*** or ***2b***–***2c*** and the Iso-Chemical Shielding Surface (ICSS)

To investigate the changes in the ^1^H NMR chemical shifts of the ring protons and the three methyl groups in borneol **1a** and isoborneol **2a** upon the introduction of an acetyl group or a benzoyl group, we performed the Iso-chemical Shielding Surface (ICSS) calculations based on the most stable structure of each compound **1b**–**1c** and **2b**–**2c** in the gas phase [23].

**Figure 3:**
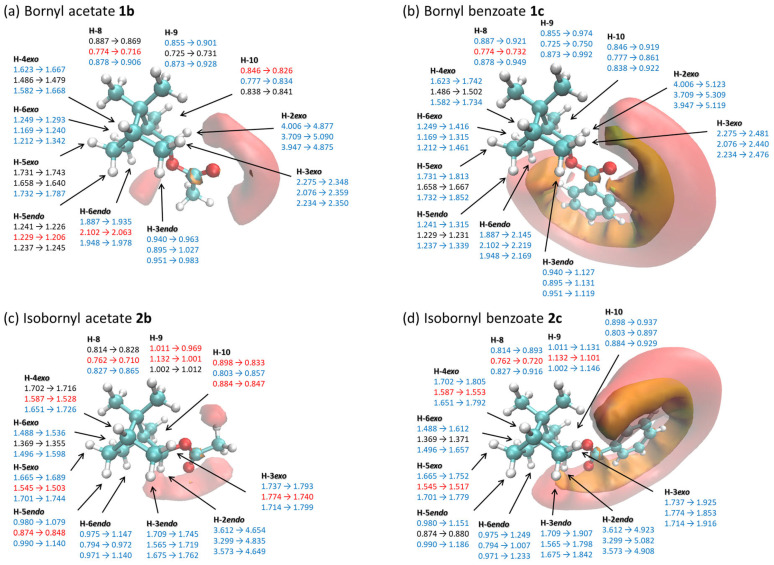
(**a**) Bornyl acetate **1b**; (**b**) Bornyl benzoate **1c**; (**c**) Isobornyl acetate **2b**; (**d**) Isobornyl benzoate **2c**. The changes in ^1^H NMR chemical shifts of borneol **1a**, isoborneol **2a**, and their derivatives containing acetyl and benzoyl groups **1b**–**1c** and **2b**–**2c**, along with their Iso-chemical Shielding Surface (ICSS). Color of ICSS: pink, ICSS = −0.2 ppm; yellow-brown, ICSS = −0.5 ppm. Solvent used for the chemical shift values: Top row, CDCl_3_; Middle row, C_6_D_6_; Bottom row, CD_3_OD. Color of changes in chemical shifts: Red, upfield shift upon the protection; Blue, downfield shift upon the protection; Black, less than ±0.02 ppm upon the protection.

The results are shown in Figure 3. The values of the ^1^H NMR chemical shifts shown in the top row represent the changes in CDCl_3_, those in the middle row represent changes in C_6_D_6_, and those in the bottom row represent changes in CD_3_OD. For each compound, the change in the ^1^H NMR chemical shift of each proton is expressed relative to unprotected **1a** or **2a**, respectively. The chemical shifts of protons that exhibited upfield shifts due to the introduction of the protecting group are indicated in red, while those that showed downfield shifts are indicated in blue. The ICSS is represented by a pink color for regions where ICSS = −0.2 ppm, while regions where ICSS = −0.5 ppm are shown in a yellow-brown color.

Figure 3a illustrates the relationship between the ^1^H NMR chemical shift changes and the ICSS regions for bornyl acetate **1b**. In bornyl acetate **1b**, a small region where ICSS = −0.2 ppm was observed, along with a tiny region where ICSS = −0.5 ppm around the carbonyl group. Therefore, the influence of the anisotropic effect due to the carbonyl group is considered to be small. As shown in Figure 1 and Figure 3a, the introduction of an acetyl group into borneol **1a** resulted in downfield shifts in the ^1^H NMR chemical shifts of the H-2*exo*, H-3*endo*, H-3*exo*, and H-6*exo* protons in all solvents. These changes are probably attributed to the electron-withdrawing nature of the acetyl group. Furthermore, it is believed that these protons exhibit downfield shifts due to the additional small deshielding effect of the carbonyl group, as indicated by the ICSS data shown in Figure 3a [67,68]. The other protons showed similar changes in CDCl_3_ and CD_3_OD. However, in C_6_D_6_, protons such as H-5*endo*, H-6*endo*, and the H-8 methyl group exhibited upfield shifts. These changes are likely to be due to the anisotropy effects from the solvent.

The introduction of an acetyl group to the hydroxyl group of borneol **1a** caused most protons to exhibit downfield shifts. Furthermore, as shown in Figure 3b, which exhibits large ICSS regions around the benzoyl group, when a benzoyl group was introduced to borneol **1a**, most protons exhibited downfield shifts in all solvents. In particular, the H-2*exo*, H-3*endo*, H-3*exo*, and H-6*endo* protons showed significant downfield shifts due to the introduction of the benzoyl group. These changes can be attributed not only to the electron-withdrawing nature of the benzoyl group, but also to the ring-current effect caused by the benzene ring according to the ICSS results.

Figure 3c shows the relationship between the chemical shifts and the ICSS regions for isobornyl acetate **2b**. As observed with bornyl acetate **1b**, there was almost no region where ICSS = −0.5 ppm, and the region where ICSS = −0.2 ppm was present near the carbonyl group. Similar to borneol acetate **1b**, the ring protons excluding the methyl group showed a similar downfield-shift trend in CDCl_3_ and CD_3_OD, while in C_6_D_6_, they exhibited chemical shift changes toward upfield shifts. Especially, H-2*endo*, H-3*endo*, and H-6*endo* showed downfield shifts in all solvents as shown in Figure 3c. These changes are likely attributed to the additional deshielding effect of the carbonyl group combined with the electron-withdrawing property of the acetyl group as inferred from the ICSS region of the carbonyl group.

Figure 3d shows the relationship between the ^1^H NMR chemical shift changes and the ICSS regions for isobornyl benzoate **2c**. Similar to bornyl benzoate **1c**, the H-2*endo*, H-3*endo*, H-3*exo*, and H-6*endo* protons, which are located near the deshielded region of the benzene ring as calculated by the ICSS, showed downfield shifts in all solvents. These chemical shifts are considered to be due to the anisotropic effect of the benzene ring. Furthermore, the downfield shifts observed for the hydrogen atoms of the H-10 methyl group is thought to be due to their proximity to the deshielded region of the benzene ring.

The methyl groups of isoborneol derivatives **2b** and **2c** exhibited a mixture of upfield and downfield shifts upon the introduction of acetyl or benzoyl groups to isoborneol **2a**, as shown in Figure 3c,d.

### 2.3. Relationship Between the Changes in ^1^H NMR Chemical Shifts of Borneol ***1a***, Isoborneol ***2a***, and Their Derivatives Containing Various Silyl Protective Groups ***1d***–***1g*** and ***2d***–***2g*** and the Iso-Chemical Shielding Surface (ICSS)

**Figure 4:**
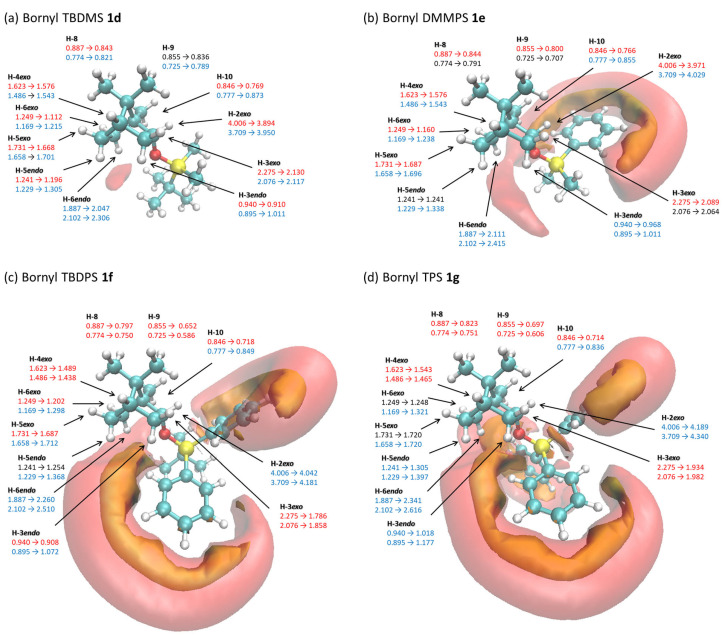
(**a**) Bornyl TBDMS **1d**; (**b**) Bornyl DMMPS **1e**; (**c**) Bornyl TBDPS **1f**; (**d**) Bornyl TPS **1g**. The changes in ^1^H NMR chemical shifts of borneol **1a** and its derivatives containing various silyl protective groups **1d**–**1g** along with their Iso-chemical Shielding Surface (ICSS). Color of ICSS: pink, ICSS = −0.2 ppm; yellow-brown, ICSS = −0.5 ppm. Solvent used for the chemical shift values: Top row, CDCl_3_; Bottom row, C_6_D_6_. Color of changes in chemical shifts: Red, upfield shift upon the protection; Blue, downfield shift upon the protection; Black, less than ±0.02 ppm upon the protection.

**Figure 5:**
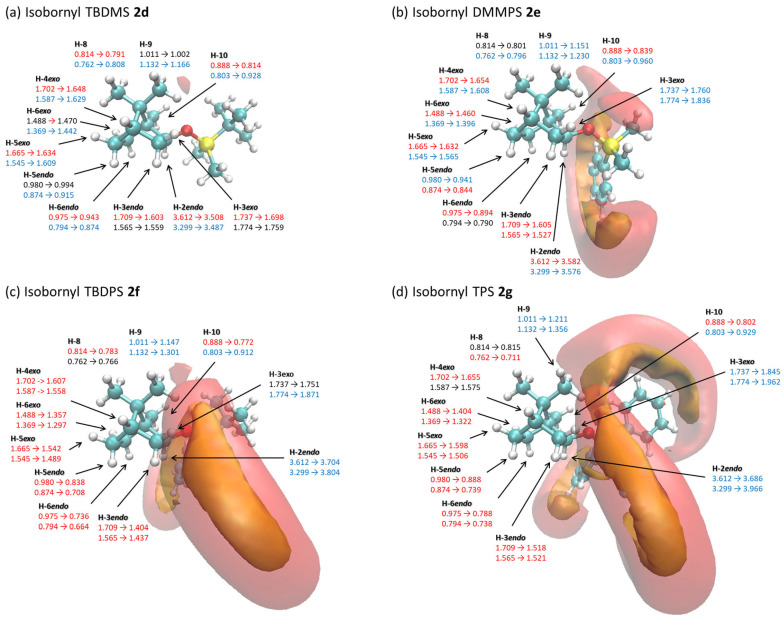
(**a**) Isobornyl TBDMS **2d**; (**b**) Isobornyl DMMPS **2e**; (**c**) Isobornyl TBDPS **2f**; (**d**) Isobornyl TPS **2g**. The changes in ^1^H NMR chemical shifts of isoborneol **2a** and its derivatives containing various silyl protective groups **2d**–**2g**, along with their Iso-chemical Shielding Surface (ICSS). Color of ICSS: pink, ICSS = −0.2 ppm; yellow-brown, ICSS = −0.5 ppm. Solvent used for the chemical shift values: Top row, CDCl_3_; Bottom row, C_6_D_6_. Color of changes in chemical shifts: Red, upfield shift upon the protection; Blue, downfield shift upon the protection; Black, less than ±0.02 ppm upon the protection.

Next, we examined the relationship between the ^1^H NMR chemical shift changes of borneol **1a** and isoborneol **2a** on the protection of the hydroxy groups with silyl-protecting groups having varying numbers of phenyl groups and the ICSS calculated from the most stable structures of the silyl-protected derivatives **1d**–**1g** and **2d**–**2g** (Figure 4 and Figure 5). In these figures, the values of the chemical shifts in the upper row represent the changes in CDCl_3_, while the values in the lower row represent the changes in C_6_D_6_. As in the previous section, the chemical shifts of hydrogens that exhibited upfield shifts due to the introduction of the protecting group are indicated in red, those that showed downfield shifts are indicated in blue, and those with almost no change (±0.02 ppm) are shown in black.

In bornyl TBDMS **1d**, with the introduction of a TBDMS group, which lacks a benzene ring, all of the ^1^H NMR chemical shifts of protons except for the H-6*endo* proton shifted upfield in CDCl_3_ and downfield in C_6_D_6_. These chemical shift changes were opposite to those observed in bornyl derivatives with the carbonyl-containing protective groups, particularly the acetyl group, as shown in Figure 4a. As depicted in Figure 4a, no region where ICSS = −0.5 ppm was present in bornyl TBDMS **1d**, and there was only a small region where ICSS = −0.2 ppm was observed around the oxygen atom of the silyl moiety. Therefore, the chemical shift changes observed in bornyl TBDMS **1d** are attributed to changes in electron density due to the introduction of the TBDMS silyl protecting group to the hydroxyl group of borneol **1a**.

Figure 4b shows the chemical shift changes and ICSS regions of bornyl DMMPS **1e**. Due to the presence of a benzene ring in the DMMPS group, a deshielding region was observed around the benzene ring. Among the ^1^H NMR chemical shift changes in bornyl DMMPS **1e**, both the H-3*exo* and H-9 methyl group protons exhibited upfield shifts in both CDCl_3_ and C_6_D_6_. These changes can be attributed to the anisotropic effect of the benzene ring as these protons are located in the shielding region of the benzene ring as indicated by the ICSS calculations. On the other hand, both the H-3*endo* and H-6*endo* protons showed downfield shifts, which are attributed to the ring current effect resulting from their proximity to the deshielding region of the benzene ring in the DMMPS group.

Figure 4c shows the ICSS regions and chemical shift changes of bornyl TBDPS **1f**, which contains a TBDPS group with two benzene rings. The protons H-3*exo*, H-4*exo*, the H-8 methyl group, and the H-9 methyl group of **1f**, which are located above the plane of the benzene rings, exhibited upfield shifts in both solvents due to the anisotropic effect of the benzene rings. Similarly, the downfield shifts observed for the H-2*exo* and H-6*endo* protons in **1f** can be attributed to their proximity to the deshielding region of the benzene rings in the TBDPS group.

Figure 4d shows the ICSS regions and chemical shift changes of bornyl TPS **1g**, which contains three benzene rings in the TPS group. Similar to **1f**, the protons H-3*exo*, H-4*exo*, the H-8 methyl group, and the H-9 methyl group of **1g**, which are located above the shielding region of the benzene rings, exhibited upfield shifts in both solvents. Conversely, the H-2*exo*, H-3*endo*, H-5*endo*, and H-6*endo* protons, which are close to the deshielding region of the benzene rings, showed downfield shifts. These observations demonstrate that the changes in ^1^H NMR chemical shifts when borneol **1a** is protected with silyl groups containing different numbers of phenyl groups can be understood by considering the shielding and deshielding regions indicated by the ICSS, as determined from the most stable structure of each borneol derivative.

As depicted in Figure 5a, similar to bornyl TBDMS **1d** in Figure 4a, no ICSS = −0.5 ppm region was observed for isobornyl TBDMS **2d**, and only a small −0.2 ppm region was present around the oxygen atom. Furthermore, when isoborneol **2a** was protected with a TBDMS group, chemical shift changes similar to those of bornyl TBDMS **1d** were observed. These changes were attributed to the change in electron density of isoborneol **2a** due to the protection with TBDMS, as indicated by the ICSS analysis results similar to bornyl TBDMS **1d**.

Figure 5b shows the relationship between the chemical shift changes of isobornyl DMMPS **2e** containing a benzene ring and the ICSS regions. Upfield shifts were observed for the H-3*endo* proton in both solvents. Considering the shielding regions of the benzene ring depicted by the ICSS, the upfield shifts of the H-3*endo* proton are thought to be due to their positions within the shielding region of the benzene ring. On the other hand, the downfield shifts observed for the H-3*exo* proton and the H-9 methyl group protons could be attributed to their proximity to the deshielding region of the benzene ring in the DMMPS group.

Figure 5c shows the correlation between the chemical shift changes and the ICSS regions of isobornyl TBDPS **2f**, which contains two benzene rings. The chemical shift changes of the H-2*endo* proton differed from those observed for the H-2*endo* proton of **2d** and **2e** with downfield shifts observed in both solvents. According to the ICSS analysis, this chemical shift change of H-2*endo* of **2f** can be attributed to the benzene rings of the TBDPS group being located in the deshielding region. Similarly, the upfield shifts observed for the H-3*endo*, H-4*exo*, H-5*endo*, H-5*exo*, H-6*endo*, and H-6*exo* protons are attributed to their positions within the shielding region of the benzene rings, while the downfield shifts observed for the H-3*exo* and H-9 methyl group protons are presumed to result from their proximity to the deshielding region of the benzene rings.

Finally, Figure 5d shows the correlation between the chemical shift changes of the hydrogen atoms observed in isobornyl TPS **2g** and its ICSS. As shown in Figure 5d, the chemical shift changes observed in isobornyl TPS **2g** exhibited a pattern similar to those observed in isobornyl TBDPS **2f**. In the isoborneol derivatives **2e**–**2g**, which have silyl protecting groups containing one to three benzene rings, the H-9 methyl group exhibited downfield shifts in both solvents, while the H-8 and H-10 methyl groups showed similar changes as shown in Figure 5b–d. Based on the ICSS analysis, the downfield shifts of the H-9 methyl group are considered to be due to the ring current effects of the benzene rings, whereas those of the H-8 and H-10 methyl groups are not due to the influence by the benzene ring.

In conclusion, it has been demonstrated that the behavior of the ^1^H NMR chemical shifts of hydrogen atoms in bornyl and isobornyl derivatives containing benzoyl groups or silyl groups with varying numbers of benzene rings can be explained by consideration of the shielding and deshielding regions of the benzene rings based on ICSS analysis of the most stable structure of each compound.

## 3. Materials and Methods

### 3.1. Chemicals

(–)-Borneol **1a**, purity > 95% (GC), and (±)-isobornel **2a**, purity > 90% (GC) were purchased from Tokyo Chemical Industry Co., Ltd. (Tokyo, Japan), and were used without further purification. The deuterated solvents, CDCl_3_, C_6_D_6_, and CD_3_OD and other reagents were purchased from Fujifilm Wako Chemicals Co., Ltd. (Osaka, Japan). Borneol derivatives **1b**–**1g** and isoborneol derivatives **2b**–**2g** were prepared from **1a** and **2a**, respectively, according to the reported procedure [20,21].

### 3.2. NMR Measurements

All NMR spectra were recorded using a JEOL JNM-ECZ400S NMR spectrometer (^1^H: 399.9 MHz, ^13^C: 100.5 MHz, JEOL Ltd., Tokyo, Japan) at 298 K with 5 mm (o.d.) Pyrex glass tubes. ^1^H and ^13^C NMR chemical shifts were measured with reference to the residual ^1^H and ^13^C signals of the deuterated solvents (CDCl_3_: δ_H_ 7.260, δ_C_ 77.01; C_6_D_6_: δ_H_ 7.156, δ_C_ 128.03, CD_3_OD: δ_H_ 3.306, δ_C_ 49.04) [69]. The ^1^H and ^13^C NMR chemical shifts of borneol **1a** and isoborneol **2a** and their derivatives **1b**–**1g** and **2b**–**2g** in CDCl_3_, C_6_D_6_ and CD_3_OD are listed in Tables S1–S8, respectively.

### 3.3. Computational Methods

Conformational search of borneol **1a**, isoborneol **2a**, and their derivatives (**1b**–**1g** and **2b**–**2g**) was performed with the use of the Spartan’06 molecular modeling program [70] with the semi-empirical method, AM1. Each compound was structurally optimized at the B3LYP/3-31G(d) level with the Gaussian 09 program from the conformers obtained by the scan of dihedral angles on the potential energy surface. The most stable structures of borneol **1a**, isoborneol **2a**, and their derivatives (**1b**–**1g** and **2b**–**2g**) were further optimized at the B3LYP/6-311+G(2d,p) level in the gas phase. The ^1^H NMR and ^13^C NMR chemical shifts of the compounds calculated at the GIAO/B3LYP/6-311+G(2d,p) level, GIAO/ωB97XD/6-311+G(2d,p) level, and GIAO/*m*PW1PW91/6-311+G(2d,p) level with the use of the integral equation formalism model of the polarizable continuum model (IEFPCM) at the same theory level, were referenced with respect to the standard TMS that was optimized at the same level [48,49,50,51,52,53,54,55,56,57,58,59,60]. The calculations of anisotropic effects are based on the nucleus independent chemical shift (NICS) concept, and the iso-chemical shielding calculations were performed with the GIAO method with the use of the HF/6-31G(d) level in the gas phase [23]. The generation of the Gaussian input files for the NICS calculations and the analysis of ICSS were performed with the use of Multiwfn 3.8 [71]. The iso-chemical surface maps were rendered by means of Visual Molecular Dynamics (VMD) software [72] based on the files exported from Multiwfn.

## 4. Conclusions

In conclusion, complete assignments of both ^1^H and ^13^C NMR chemical shifts for borneol **1a**, isoborneol **2a**, and their derivatives **1b**–**1g** and **2b**–**2g,** in which the hydroxy groups were protected with various protecting groups, were made in two solvents, and the validity of these assignments has further been confirmed by quantum chemical calculations at the GIAO/*m*PW1PW91/6-311+G(2d,p) level. The origins of the chemical shift changes upon protection of the hydroxy groups were further investigated by the ICSS method. The ICSS results suggest some regions may have been affected by the anisotropic effects from the phenyl rings or the carbonyl groups in the protecting groups. To our knowledge, this is the first study reporting the complete assignments of ^1^H and ^13^C NMR chemical shifts of borneol **1a** and isoborneol **2a** and their derivatives in various solvents and also reports their strong correlations with those predicted by quantum chemical calculations.

## Data Availability

The original contributions presented in the study are included in the article/Supplementary Materials, and further inquiries can be directed to the corresponding author.

## Supplementary Materials

The following supporting information can be downloaded at: https://www.mdpi.com/article/10.3390/molecules30030597/s1, Figure S1. ^1^H NMR chemical shift changes of isoborneol **2a** and its derivatives **2b**–**2c** in different solvents (CDCl_3_, C_6_D_6_ or CD_3_OD); Figure S2. ^1^H NMR chemical shift changes of isoborneol **2a** and its derivatives **2d**–**2g** in different solvents (CDCl_3_ or C_6_D_6_); Figure S3. Correlations between the experimental ^1^H and ^13^C NMR chemical shifts of borneol **1** and its derivatives **1b**–**1c** in different solvents (CDCl_3_, C_6_D_6_, or CD_3_OD) and their calculated values (in the gas phase) at the GIAO/*m*PW1PW91/6-311+G(2d,p) level; Figure S4. Correlations between the experimental ^1^H NMR chemical shifts of borneol **1** and its derivatives **1b**–**1c** in different solvents (CDCl_3_, C_6_D_6_, or CD_3_OD) and their calculated values in the corresponding solvent at the GIAO/*m*PW1PW91/6-311+G(2d,p) level; Figure S5. Correlations between the experimental ^1^H and ^13^C NMR chemical shifts of isoborneol **2a** and its derivatives **2b**–**2c** in different solvents (CDCl_3_, C_6_D_6_, or CD_3_OD) and their calculated values (in the gas phase) at the GIAO/*m*PW1PW91/6-311+G(2d,p) level; Figure S6. Correlations between the experimental ^1^H NMR chemical shifts of isoborneol **2a** and its derivatives **2b**–**2c** in different solvents (CDCl_3_, C_6_D_6_, or CD_3_OD) and their calculated values in the corresponding solvent at the GIAO/*m*PW1PW91/6-311+G(2d,p) level; Figure S7. Correlations between the experimental ^1^H and ^13^C NMR chemical shifts of bornyl derivatives **1d**–**1g** in different solvents (CDCl_3_ or C_6_D_6_) and their calculated values (in the gas phase) at the GIAO/*m*PW1PW91/6-311+G(2d,p) level; Figure S8. Correlations between the experimental ^1^H NMR chemical shifts of bornyl derivatives **1d**–**1g** in different solvents (CDCl_3_ or C_6_D_6_) and their calculated values in the corresponding solvent at the GIAO/*m*PW1PW91/6-311+G(2d,p) level; Figure S9. Correlations between the experimental ^1^H NMR and ^13^C NMR chemical shifts of isobornyl derivatives **2d**–**2g** in different solvents (CDCl_3_ or C_6_D_6_) and their calculated values (in the gas phase) at the GIAO/*m*PW1PW91/6-311+G(2d,p) level; Figure S10. Correlations between the experimental ^1^H NMR chemical shifts of isobornyl derivatives **2d**–**2g** in different solvents (CDCl_3_ or C_6_D_6_) and their calculated values in the corresponding solvent at the GIAO/*m*PW1PW91/6-311+G(2d,p) level; Table S1. ^1^H NMR chemical shifts for borneol **1** and its derivatives **1b**–**1c** in different solvents; Table S2. ^13^C NMR chemical shifts for borneol **1** and its derivatives **1b**–**1c** in different solvents; Table S3. ^1^H NMR chemical shifts for isoborneol **2a** and its derivatives **2b**–**2c** in different solvents; Table S4. ^13^C NMR chemical shifts for isoborneol **2a** and its derivatives **2b**–**2c** in different solvents; Table S5. ^1^H NMR chemical shifts of borneol **1a** and its derivatives **1b**–**1e** in CDCl_3_ and C_6_D_6_; Table S6. ^13^C NMR chemical shifts of borneol **1a** and its derivatives **1b**–**1e** in CDCl_3_ and C_6_D_6_; Table S7. ^1^H NMR chemical shifts of isoborneol **2a** and its derivatives **2b**–**2e** in CDCl_3_ and C_6_D_6_; Table S8. ^13^C NMR chemical shifts of isoborneol **2a** and its derivatives **2b**–**2e** in CDCl_3_ and C_6_D_6_; Table S9: Optimized coordinates, energies, and calculated NMR chemical shifts of borneol **1a**; Table S10: Optimized coordinates, energies, and calculated NMR chemical shifts of isoborneol **2a**; Table S11: Optimized coordinates, energies, and calculated NMR chemical shifts of bornyl acetate **1b**; Table S12: Optimized coordinates, energies, and calculated NMR chemical shifts of bornyl benzoate **1c**; Table S13: Optimized coordinates, energies, and calculated NMR chemical shifts of isobornyl acetate **2b**; Table S14: Optimized coordinates, energies, and calculated NMR chemical shifts of isobornyl benzoate **2c**; Table S15: Optimized coordinates, energies, and calculated NMR chemical shifts of bornyl TBDMS **1d**; Table S16: Optimized coordinates, energies, and calculated NMR chemical shifts of bornyl DMMPS **1e**; Table S17: Optimized coordinates, energies, and calculated NMR chemical shifts of bornyl TBDPS **1f**; Table S18: Optimized coordinates, energies, and calculated NMR chemical shifts of bornyl TPS **1g**; Table S19: Optimized coordinates, energies, and calculated NMR chemical shifts of isobornyl TBDMS **2d**; Table S20: Optimized coordinates, energies, and calculated NMR chemical shifts of isobornyl DMMPS **2e**; Table S21: Optimized coordinates, energies, and calculated NMR chemical shifts of isobornyl TBDPS **2f**; Table S22: Optimized coordinates, energies, and calculated NMR chemical shifts of isobornyl TPS **2g**; Table S23: Comparison of the coefficient of determination (*r*^2^) and RMS between experimental and calculated ^1^H NMR chemical shifts for compounds **1a**–**1c** and **2a**–**2c** using various calculation methods; Table S24: Comparison of the coefficient of determination (*r*^2^) and RMS between experimental and calculated ^1^H NMR chemical shifts for compounds **1d**–**1g** and **2d**–**2g** using various calculation methods; Table S25: Comparison of the coefficient of determination (*r*^2^) and RMS between experimental and calculated ^13^C NMR chemical shifts for compounds **1a**–**1c** and **2a**–**2c** using various calculation methods; Table S26: Comparison of the coefficient of determination (*r*^2^) and RMS between experimental and calculated ^13^C NMR chemical shifts for compounds **1d**–**1g** and **2d**–**2g** using various calculation methods. Video S1: 1b-Bornyl-Acetate.mp4; Video S2: 1c-Bornyl-Acetate.mp4; Video S3: 1d-Bornyl-TBDMS.mp4; Video S4: 1e-Bornyl-DMMPS.mp4; Video S5: 1f-Bornyl-TBDPS.mp4; Video S6: 1g-Bornyl-TPS.mp4; Video S7: 2b-Isobornyl-Acetate.mp4; Video S8: 2c-Isobornyl-Acetate.mp4; Video S9: 2d-Isobornyl-TBDMS.mp4; Video S10: 2e-Isobornyl-DMMPS.mp4; Video S11: 2f-Isobornyl-TBDPS.mp4; Video S12: 2g-Isobornyl-TPS.mp4.
