# Supplementary material for: Complete Assignments of 1H and 13C NMR Chemical Shift Changes Observed upon Protection of Hydroxy Group in Borneol and Isoborneol and Their DFT Verification
Source: Molecules. 2025 Jan 28;30(3):597. doi: 10.3390/molecules30030597 (PMC11819931; doi:10.3390/molecules30030597)
Supplement: Supplementary file 1 [file molecules-30-00597-s001.zip › molecules-3420509-supplementary.pdf]

Supplementary Material

Complete Assignments of ^{1}H and ^{13}C NMR Chemical Shift Changes Observed upon Protection of Hydroxy Group in Borneol and Isoborneol and Their DFT Verification

Authors:

Baohe Lyu[1], Yoshikazu Hiraga[1,*], Ryukichi Takagi[2], and Satomi Niwayama[3,*]

Affiliations:

1 Graduate School of Science and Technology, Hiroshima Institute of Technology, 2-1-1 Miyake, Saeki-ku, Hiroshima 731-5193, Japan

2 Department of Chemistry, Graduate School of Advanced Science and Engineering, Hiroshima University, 1-3-1 Kagamiyama, Higashi-Hiroshima 739-8526, Japan

3 Graduate School of Engineering, Muroran Institute of Technology, 27-1, Mizumoto-cho, Muroran 050-8585, Japan

***Correspondence:**

y.hiraga.65@it-hiroshima.ac.jp; Tel.: +81-82-921-9468 (Y.H.);

niwayama@muroran-it.ac.jp; Tel.: +81-143-46-5746 (S.N.)

Index

S4 **Figure S1.** ^{1}H NMR chemical shift changes of isoborneol **2a** and its derivatives **2b**–**2c** in different solvents ($CDCl_3$, C_6D_6 or CD_3OD).

S5 **Figure S2.** ^{1}H NMR chemical shift changes of isoborneol **2a** and its derivatives **2d**–**2g** in different solvents ($CDCl_3$ or C_6D_6).

S6 **Figure S3.** Correlations between the experimental ^{1}H and ^{13}C NMR chemical shifts of borneol **1** and its derivatives **1b**－**1c** in different solvents ($CDCl_3$, C_6D_6, or CD_3OD) and their calculated values (in the gas phase) at the GIAO/*m*PW1PW91/6-311+G(2d,p) level.

S7 **Figure S4.** Correlations between the experimental ^{1}H NMR chemical shifts of borneol **1** and its derivatives **1b**－**1c** in different solvents ($CDCl_3$, C_6D_6, or CD_3OD) and their calculated values in the corresponding solvent at the GIAO/*m*PW1PW91/6-311+G(2d,p) level.

S8 **Figure S5.** Correlations between the experimental ^{1}H and ^{13}C NMR chemical shifts of isoborneol **2a** and its derivatives **2b**－**2c** in different solvents ($CDCl_3$, C_6D_6, or CD_3OD) and their calculated values (in the gas phase) at the GIAO/*m*PW1PW91/6-311+G(2d,p) level.

S9 **Figure S6.** Correlations between the experimental ^{1}H NMR chemical shifts of isoborneol **2a** and its derivatives **2b**－**2c** in different solvents ($CDCl_3$, C_6D_6, or CD_3OD) and their calculated values in the corresponding solvent at the GIAO/*m*PW1PW91/6-311+G(2d,p) level.

S10 **Figure S7.** Correlations between the experimental ^{1}H and ^{13}C NMR chemical shifts of bornyl derivatives **1d–1g** in different solvents ($CDCl_3$ or C_6D_6) and their calculated values (in the gas phase) at the GIAO/*m*PW1PW91/6-311+G(2d,p) level.

S11 **Figure S8.** Correlations between the experimental ^{1}H NMR chemical shifts of bornyl derivatives **1d–1g** in different solvents ($CDCl_3$ or C_6D_6) and their calculated values in the corresponding solvent at the GIAO/*m*PW1PW91/6-311+G(2d,p) level.

S12 **Figure S9.** Correlations between the experimental ^{1}H NMR and ^{13}C NMR chemical shifts of isobornyl derivatives **2d–2g** in different solvents ($CDCl_3$ or C_6D_6) and their calculated values (in the gas phase) at the GIAO/*m*PW1PW91/6-311+G(2d,p) level.

S13 **Figure S10.** Correlations between the experimental ^{1}H NMR chemical shifts of isobornyl derivatives **2d–2g** in different solvents ($CDCl_3$ or C_6D_6) and their calculated values in the corresponding solvent at the GIAO/*m*PW1PW91/6-311+G(2d,p) level.

S14 **Table S1.** ^{1}H NMR chemical shifts for borneol **1** and its derivatives **1b**－**1c** in different solvents.

S15 **Table S2.** ^{13}C NMR chemical shifts for borneol **1** and its derivatives **1b**－**1c** in different solvents.

S16 **Table S3.** ^{1}H NMR chemical shifts for isoborneol **2a** and its derivatives **2b**－**2c** in different solvents.

S17 **Table S4.** ^{13}C NMR chemical shifts for isoborneol **2a** and its derivatives **2b**－**2c** in different solvents.

S18 **Table S5.** ^{1}H NMR chemical shifts of borneol **1a** and its derivatives **1b**−**1e** in $CDCl_3$ and C_6D_6.

S19 **Table S6.** ^{13}C NMR chemical shifts of borneol **1a** and its derivatives **1b**−**1e** in $CDCl_3$ and C_6D_6.

S20 **Table S7.** ^{1}H NMR chemical shifts of isoborneol **2a** and its derivatives **2b**−**2e** in $CDCl_3$ and C_6D_6.

S21 **Table S8.** ^{13}C NMR chemical shifts of isoborneol **2a** and its derivatives **2b**−**2e** in $CDCl_3$ and C_6D_6.

S22 **Table S9**: Optimized coordinates, energies, and calculated NMR chemical shifts of borneol **1a**.

S26 **Table S10**: Optimized coordinates, energies, and calculated NMR chemical shifts of isoborneol **2a**.

S30 **Table S11**: Optimized coordinates, energies, and calculated NMR chemical shifts of bornyl acetate **1b**.

S34 **Table S12**: Optimized coordinates, energies, and calculated NMR chemical shifts of bornyl benzoate **1c**.

S39 **Table S13**: Optimized coordinates, energies, and calculated NMR chemical shifts of isobornyl acetate **2b**.

S43 **Table S14**: Optimized coordinates, energies, and calculated NMR chemical shifts of isobornyl benzoate **2c**.

S47 **Table S15**: Optimized coordinates, energies, and calculated NMR chemical shifts of bornyl TBDMS **1d**.

S52 **Table S16**: Optimized coordinates, energies, and calculated NMR chemical shifts of bornyl DMMPS **1e**.

S57 **Table S17**: Optimized coordinates, energies, and calculated NMR chemical shifts of bornyl TBDPS **1f**.

S60 **Table S18**: Optimized coordinates, energies, and calculated NMR chemical shifts of bornyl TPS **1g**.

S67 **Table S19**: Optimized coordinates, energies, and calculated NMR chemical shifts of isobornyl TBDMS **2d**.

S72 **Table S20**: Optimized coordinates, energies, and calculated NMR chemical shifts of isobornyl DMMPS **2e**.

S77 **Table S21**: Optimized coordinates, energies, and calculated NMR chemical shifts of isobornyl TBDPS **2f**.

S82 **Table S22**: Optimized coordinates, energies, and calculated NMR chemical shifts of isobornyl TPS **2g**.

S87 **Table S23**: Comparison of the coefficient of determination (r^2) and RMS between experimental and calculated ^{1}H NMR chemical shifts for compounds **1a-1c** and **2a-2c** using various calculation methods.

S88 **Table S24**: Comparison of the coefficient of determination (r^2) and RMS between experimental and calculated ^{1}H NMR chemical shifts for compounds **1d–1g** and **2d–2g** using various calculation methods.

S89 **Table S25**: Comparison of the coefficient of determination (r^2) and RMS between experimental and calculated ^{13}C NMR chemical shifts for compounds **1a-1c** and **2a-2c** using various calculation methods.

S90 **Table S26**: Comparison of the coefficient of determination (r^2) and RMS between experimental and calculated ^{13}C NMR chemical shifts for compounds **1d–1g** and **2d–2g** using various calculation methods.

Video S1-S12

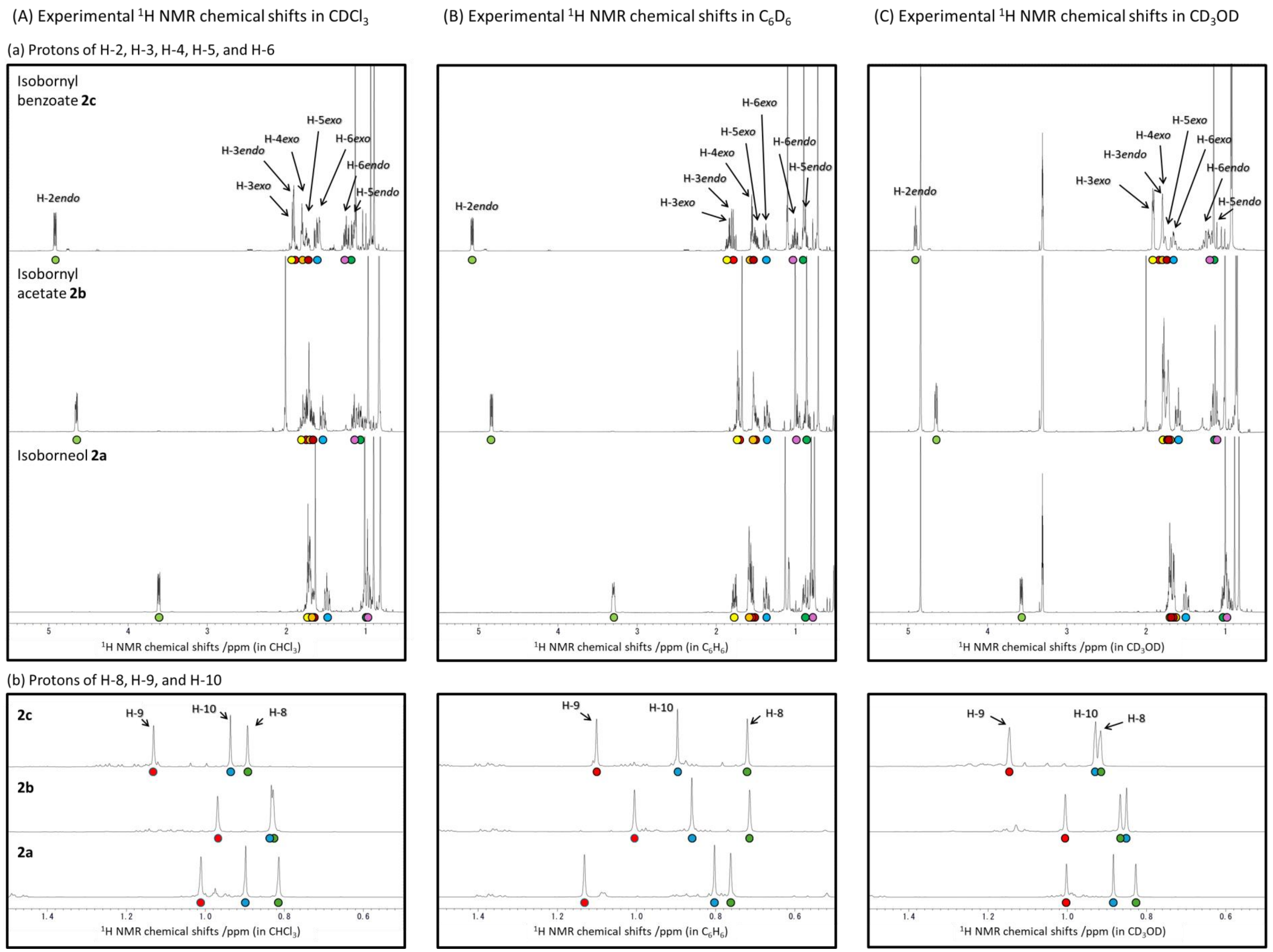

Figure S1. ^{1}H NMR chemical shift changes of isoborneol **2a** and its derivatives **2b–2c** in different solvents ($CDCl_3$, C_6D_6 or CD_3OD).

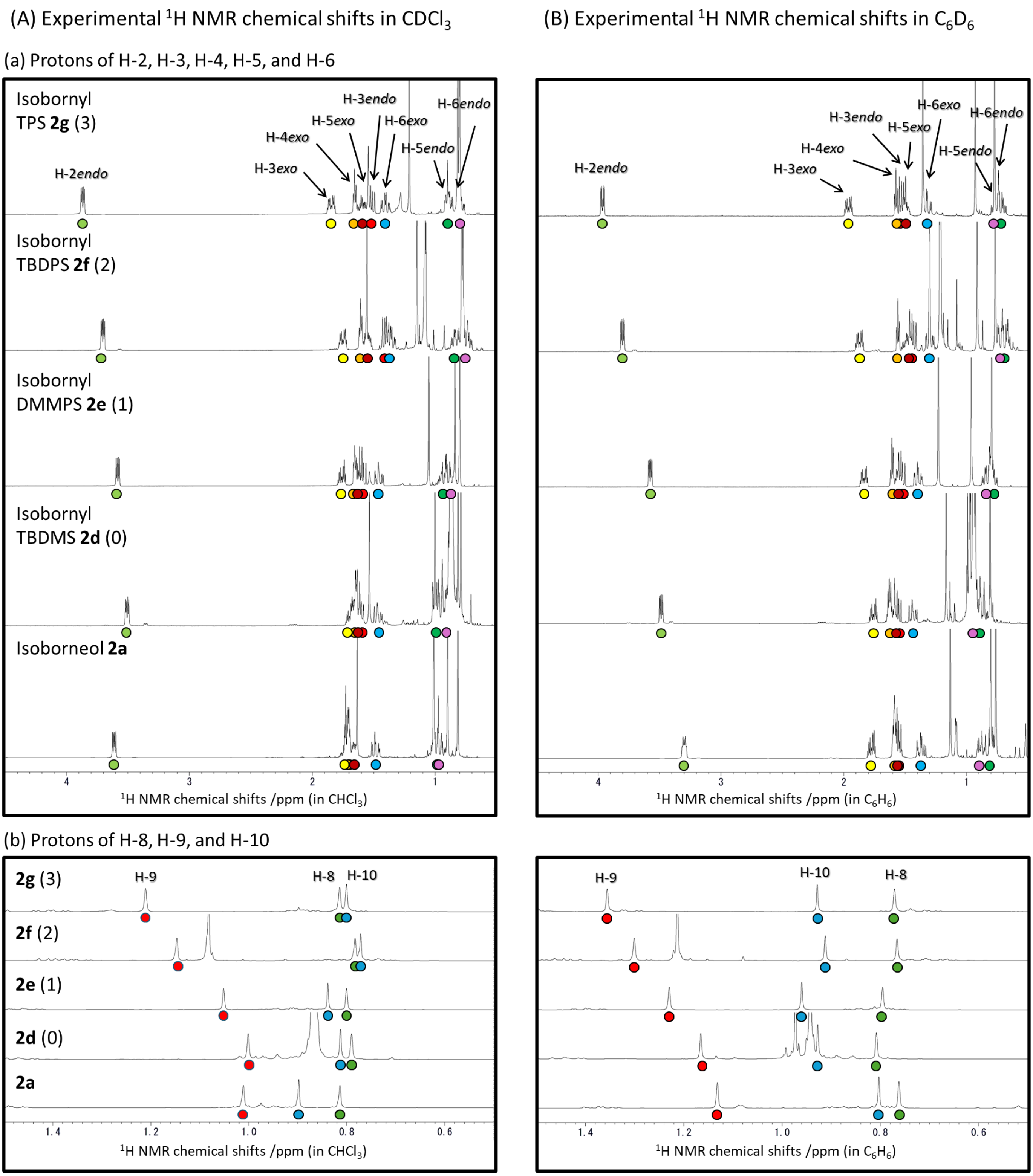

Figure S2. ¹H NMR chemical shift changes of isoborneol **2a** and its derivatives **2d–2g** in different solvents ($CDCl_3$ or C_6D_6). The number in parentheses under the compound number indicates the number of phenyl groups.

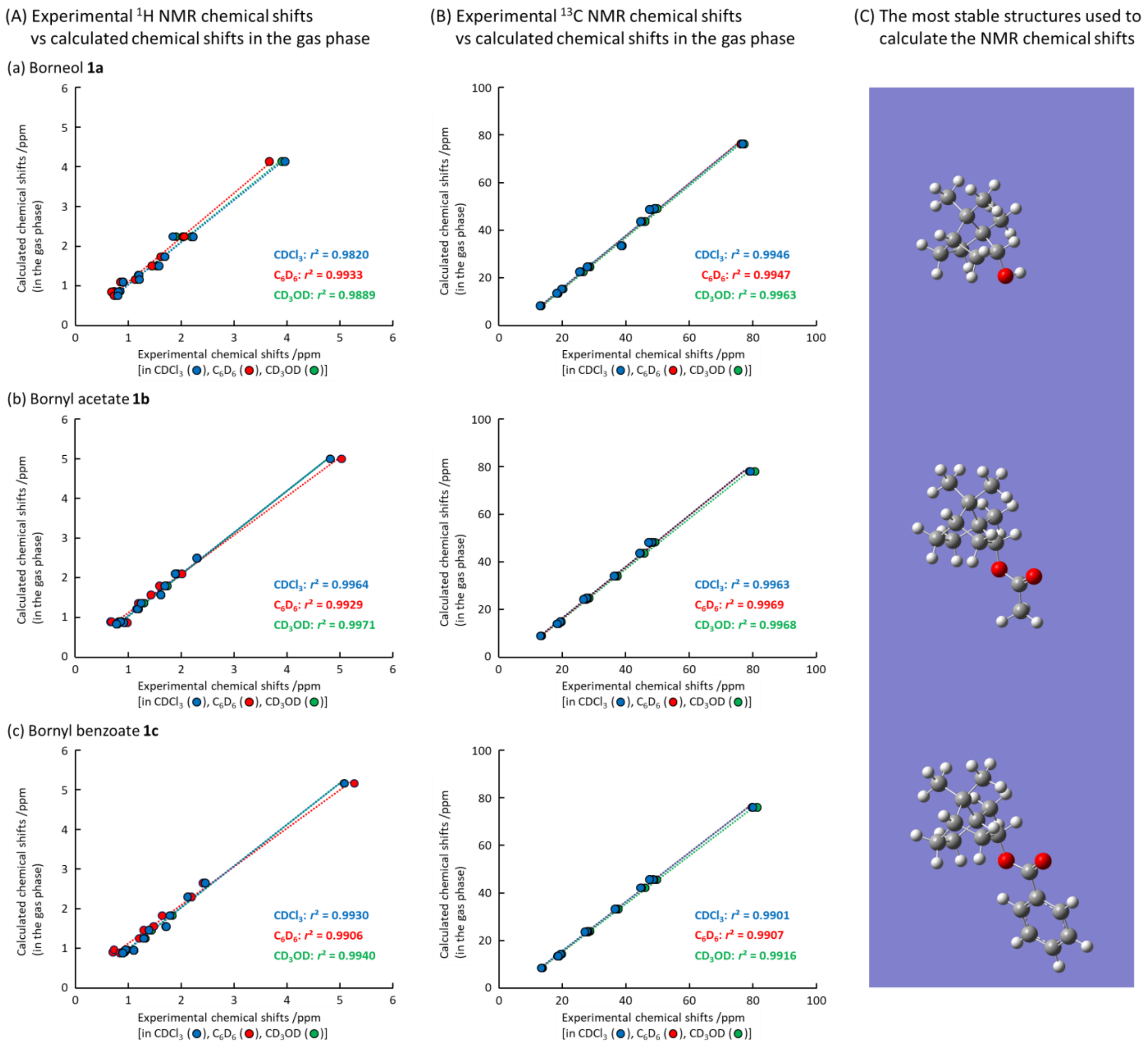

Figure S3. Correlations between the experimental ^{1}H and ^{13}C NMR chemical shifts of borneol **1a**, bornyl acetate **1b**, bornyl benzoate **1c** in different solvents ($CDCl_3$, C_6D_6, or CD_3OD) and their calculated values (in the gas phase) at the GIAO/*m*PW1PW91/6-311+G(2d,p) level. The coefficient of determination (r^2) values for **1b** and **1c** exclude the values of the substituents attached to the bicyclic ring.

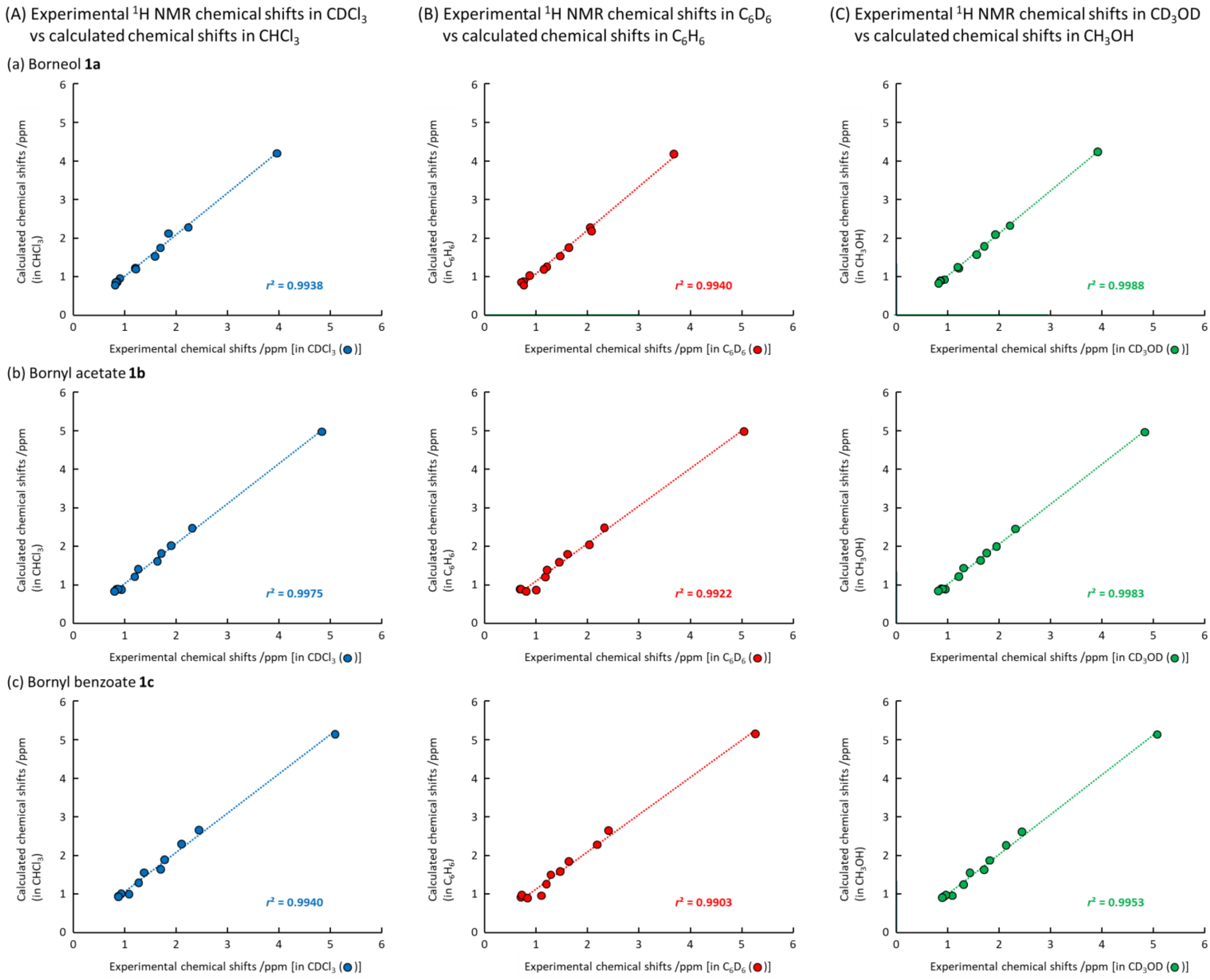

Figure S4. Correlations between the experimental ^{1}H NMR chemical shifts of borneol **1a**, bornyl acetate **1b**, bornyl benzoate **1c** in different solvents ($CDCl_3$, C_6D_6, or CD_3OD) and their calculated values in the corresponding solvent at the GIAO/*m*PW1PW91/6-311+G(2d,p) level. The coefficient of determination (r^2) values for **1b** and **1c** exclude the values of the substituents attached to the bicyclic ring.

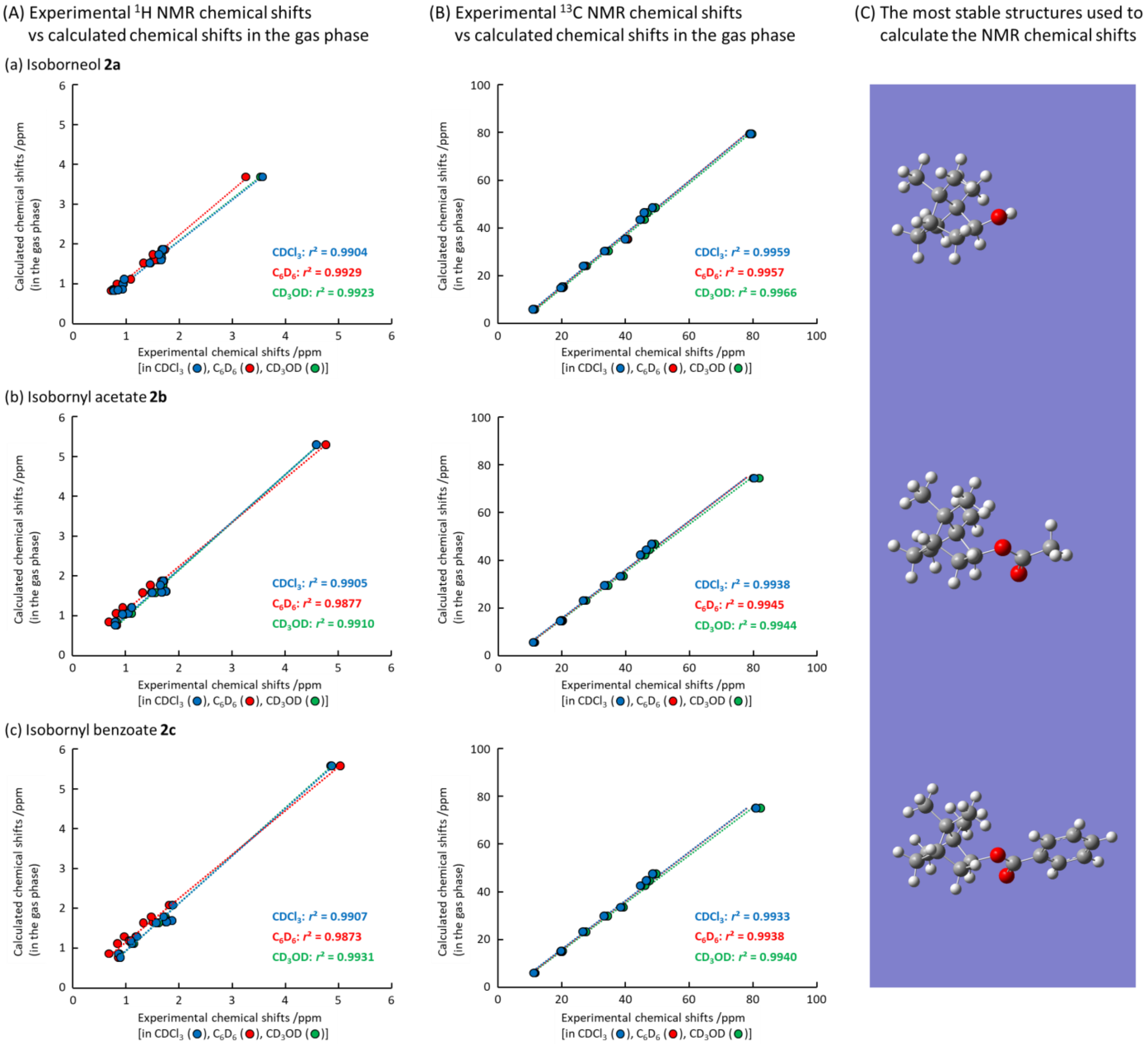

Figure S5. Correlations between the experimental ^{1}H and ^{13}C NMR chemical shifts of isoborneol **2a**, isobornyl acetate **2b**, isobornyl benzoate **2c** in different solvents ($CDCl_3$, C_6D_6, or CD_3OD) and their calculated values (in the gas phase) at the GIAO/*m*PW1PW91/6-311+G(2d,p) level. The coefficient of determination (r^2) values for **2b** and **2c** exclude the values of the substituents attached to the bicyclic ring.

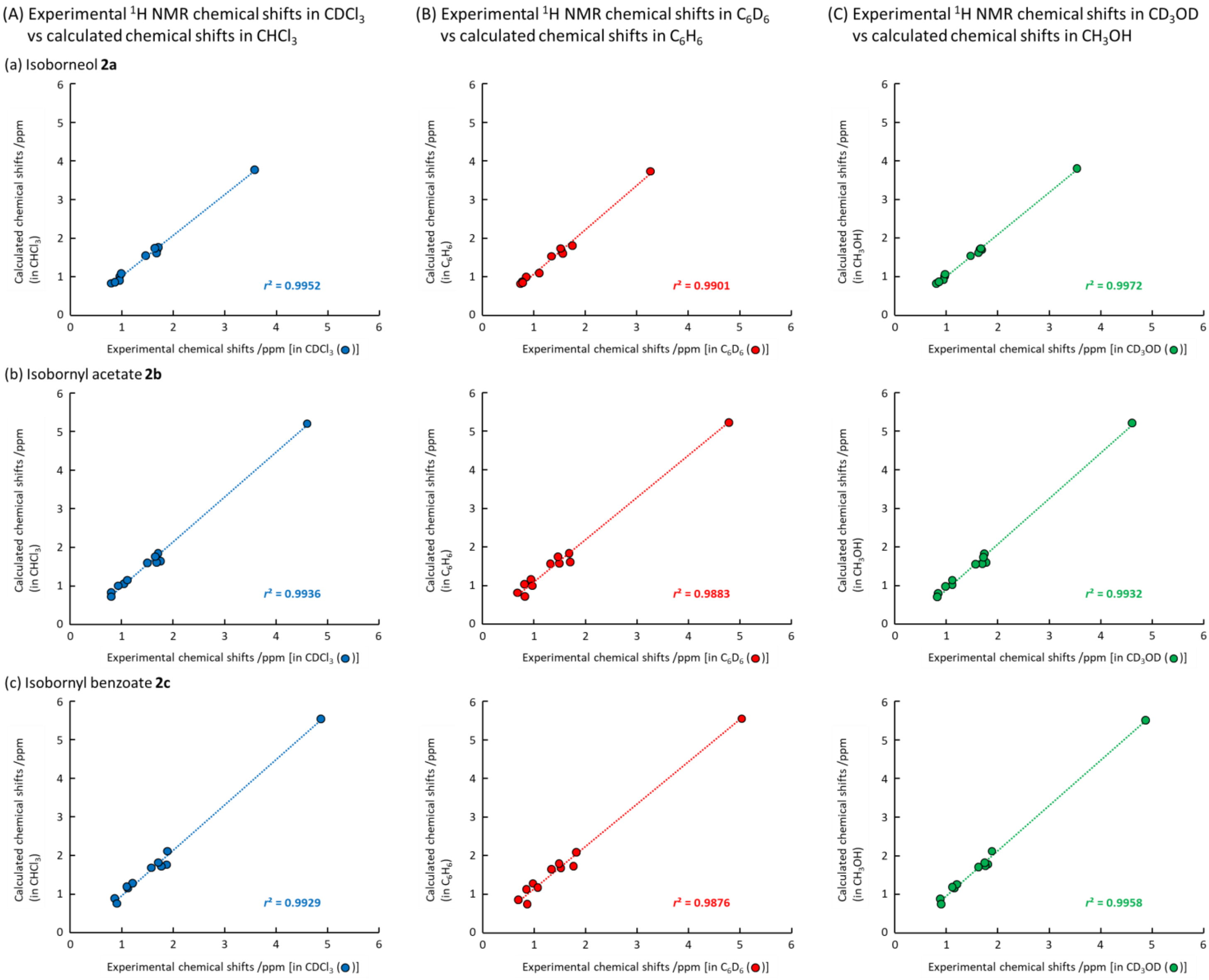

Figure S6. Correlations between the experimental ^{1}H NMR chemical shifts of isoborneol **2a**, isobornyl acetate **2b**, isobornyl benzoate **2c** in different solvents ($CDCl_3$, C_6D_6, or CD_3OD) and their calculated values in the corresponding solvent at the GIAO/*m*PW1PW91/6-311+G(2d,p) level. The coefficient of determination (r^2) values for **2b** and **2c** exclude the values of the substituents attached to the bicyclic ring.

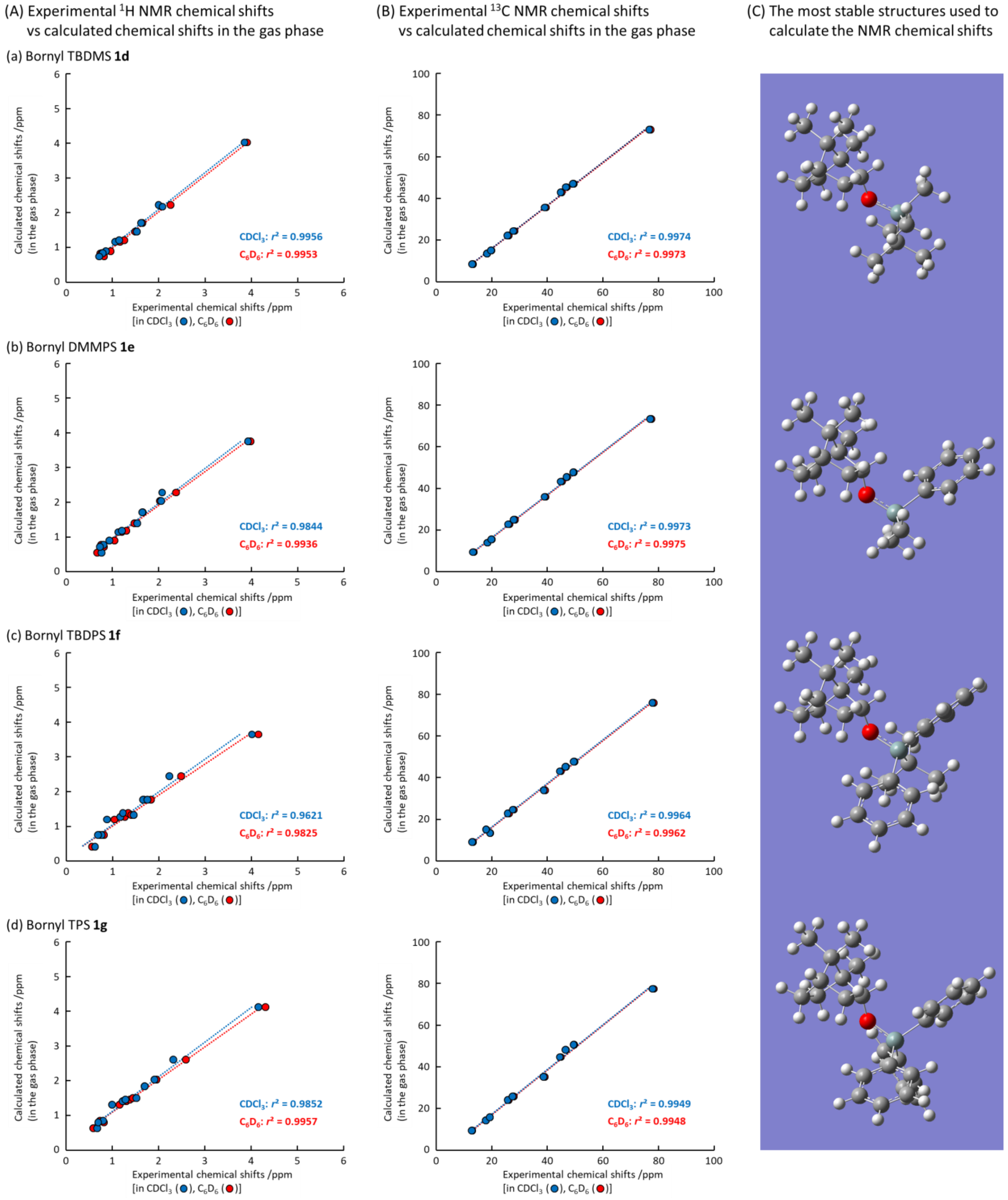

Figure S7. Correlations between the experimental ¹H and ¹³C NMR chemical shifts of bornyl derivatives **1d**–**1g** in different solvents ($CDCl_3$ or C_6D_6) and their calculated values (in the gas phase) at the GIAO/*m*PW1PW91/6-311+G(2d,p) level. The coefficient of determination (r^2) values for **1d**–**1g** exclude the values of the substituents attached to the bicyclic ring.

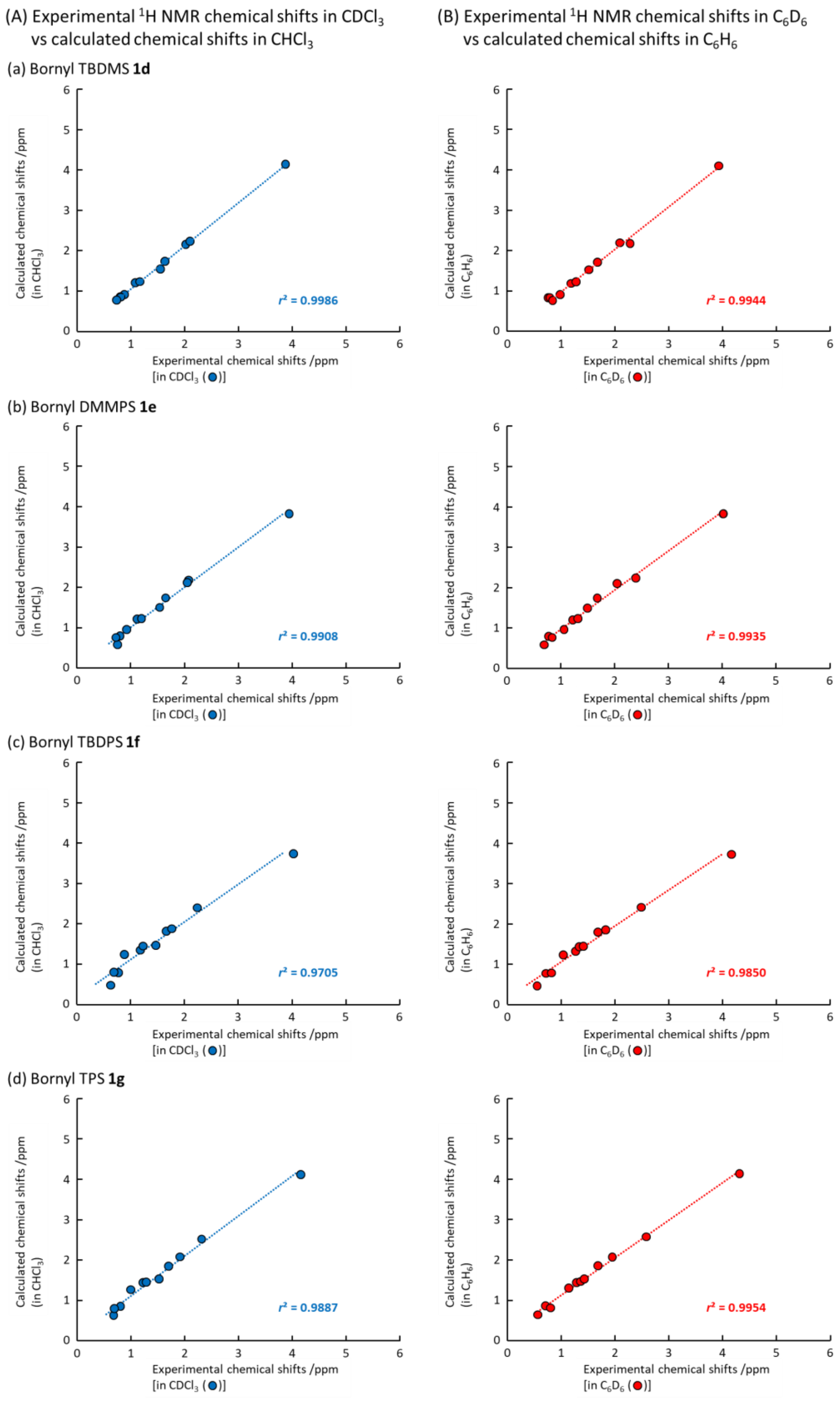

Figure S8. Correlations between the experimental ^{1}H NMR chemical shifts of bornyl derivatives **1d–1g** in different solvents ($CDCl_3$ or C_6D_6) and their calculated values in the corresponding solvent at the GIAO/*m*PW1PW91/6-311+G(2d,p) level. The coefficient of determination (r^2) values for **1d–1g** exclude the values of the substituents attached to the bicyclic ring.

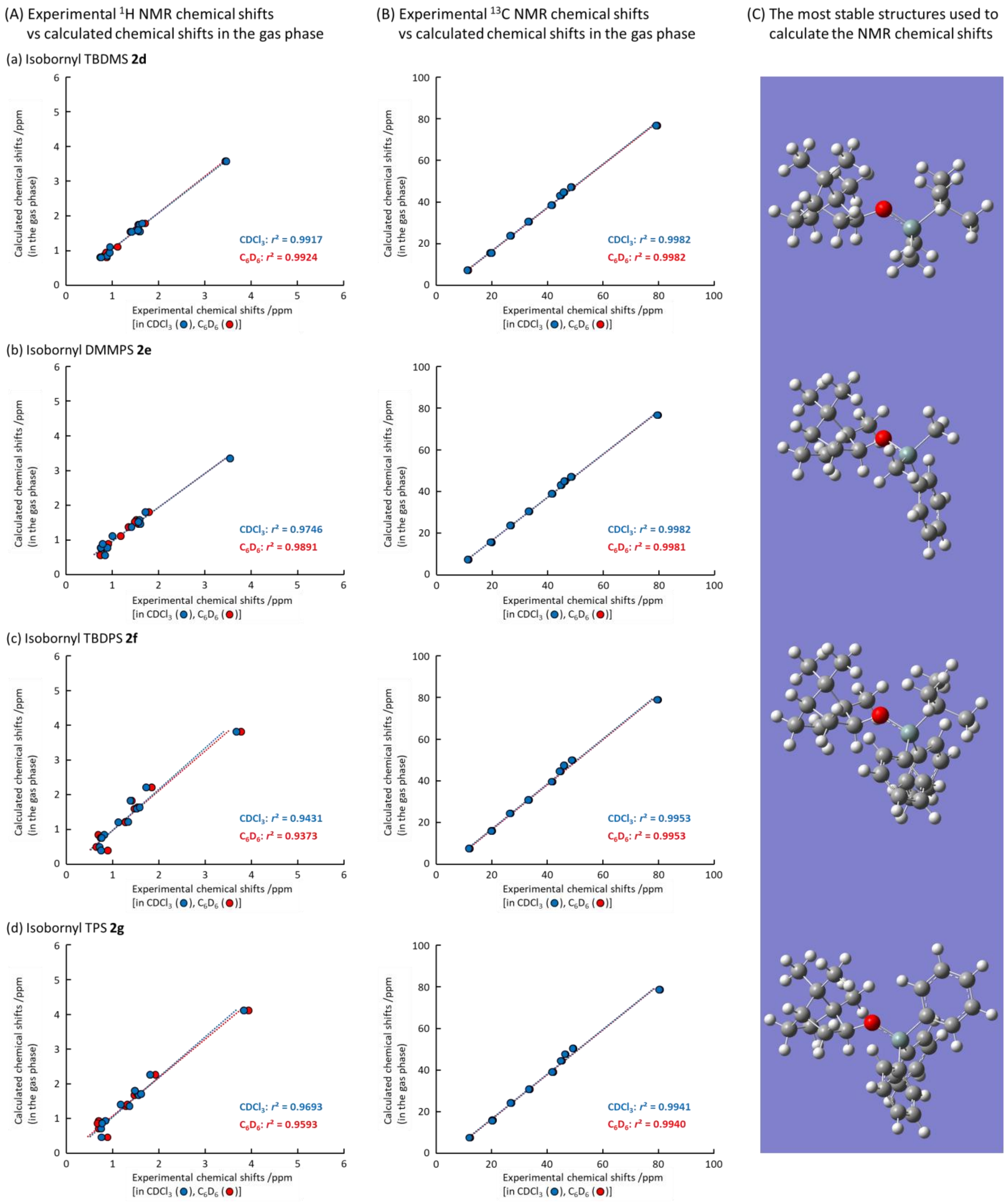

Figure S9. Correlations between the experimental ^{1}H NMR and ^{13}C NMR chemical shifts of isobornyl derivatives **2d–2g** in different solvents ($CDCl_3$ or C_6D_6) and their calculated values (in the gas phase) at the GIAO/*m*PW1PW91/6-311+G(2d,p) level. The coefficient of determination (r^2) values for **2d–2g** exclude the values of the substituents attached to the bicyclic ring.

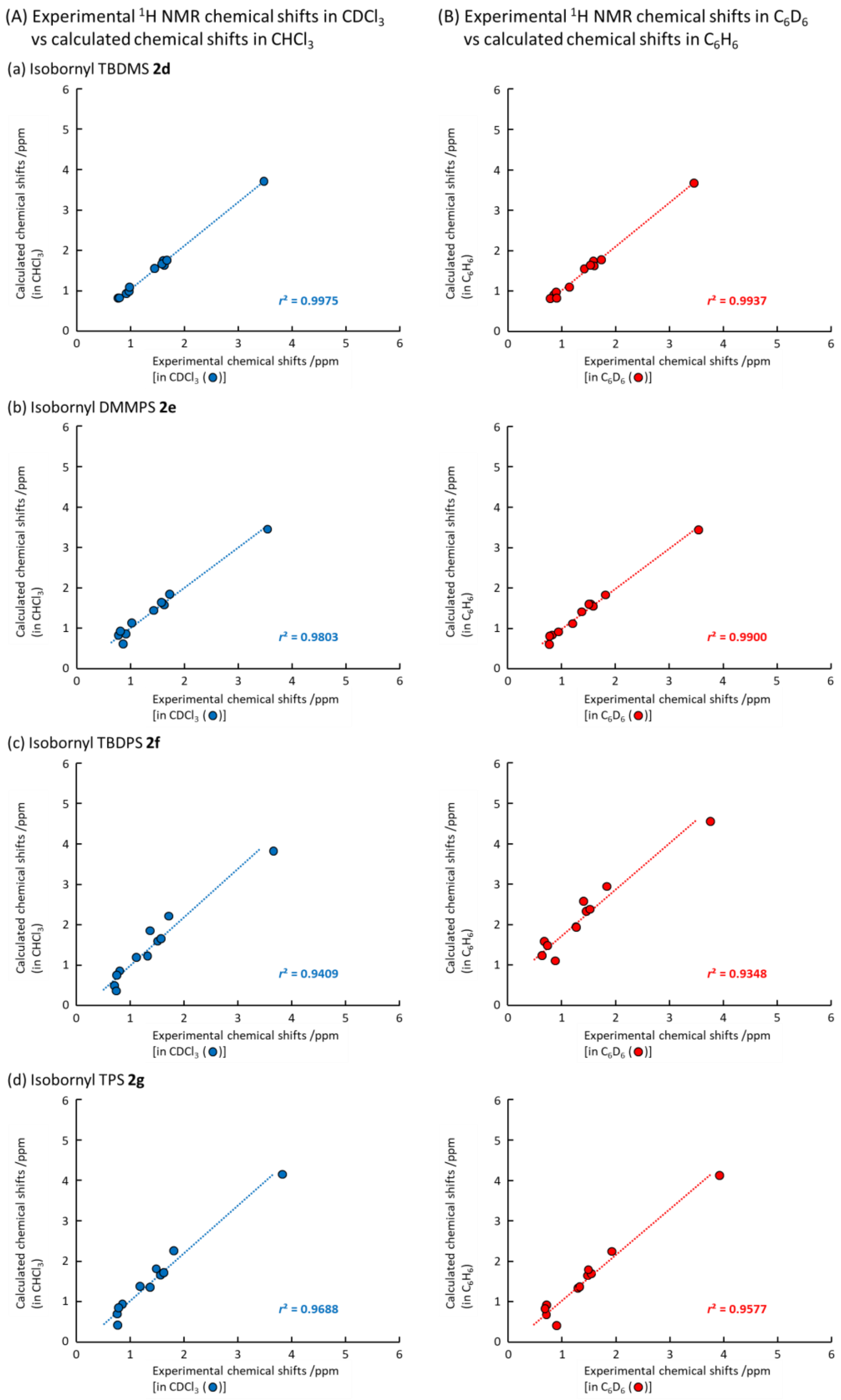

Figure S10. Correlations between the experimental ^{1}H NMR chemical shifts of isobornyl derivatives **2d**–**2g** in different solvents ($CDCl_3$ or C_6D_6) and their calculated values in the corresponding solvent at the GIAO/*m*PW1PW91/6-311+G(2d,p) level. The coefficient of determination (r^2) values for **2d**–**2g** exclude the values of the substituents attached to the bicyclic ring.

Table S1. ^{1}H NMR chemical shifts (in ppm) for borneol **1a**, bornyl acetate **1b**, and bornyl benzoate **1c** in different solvents

	$CDCl_3$ δ (^{1}H) ppm			C_6D_6 δ (^{1}H) ppm			CD_3OD δ (^{1}H) ppm		
Atom	Borneol **1a**	Bornyl acetate **1b**	Bornyl benzoate **1c**	Borneol **1a**	Bornyl acetate **1b**	Bornyl benzoate **1c**	Borneol **1a**	Bornyl acetate **1b**	Bornyl benzoate **1c**
H-2*exo*	4.006	4.877	5.123	3.709	5.090	5.309	3.947	4.875	5.119
H-3*endo*	0.940	0.963	1.127	0.895	1.027	1.131	0.951	0.983	1.119
H-3*exo*	2.275	2.348	2.481	2.076	2.359	2.440	2.234	2.350	2.476
H-4	1.623	1.667	1.742	1.486	1.479	1.502	1.582	1.668	1.734
H-5*endo*	1.241	1.226	1.315	1.229	1.206	1.231	1.237	1.245	1.339
H-5*exo*	1.731	1.743	1.813	1.658	1.640	1.667	1.732	1.787	1.852
H-6*endo*	1.887	1.935	2.145	2.102	2.063	2.219	1.948	1.978	2.169
H-6*exo*	1.249	1.293	1.416	1.169	1.240	1.315	1.212	1.342	1.461
H-8	0.887	0.869	0.921	0.774	0.716	0.732	0.878	0.906	0.949
H-9	0.855	0.901	0.974	0.725	0.731	0.750	0.873	0.928	0.992
H-10	0.846	0.826	0.919	0.777	0.834	0.861	0.838	0.841	0.922
H-12		2.057			1.706			2.040	
H-14*o*			8.066			8.209			8.019
H-15*m*			7.450			7.068			7.483
H-16*p*			7.560			7.111			7.602

Table S2. ^{13}C NMR chemical shifts (in ppm) for borneol **1a**, bornyl acetate **1b**, and bornyl benzoate **1c** in different solvents

	$CDCl_3$ δ (^{13}C) ppm			C_6D_6 δ (^{13}C) ppm			CD_3OD δ (^{13}C) ppm		
Atom	Borneol **1a**	Bornyl acetate **1b**	Bornyl benzoate **1c**	Borneol **1a**	Bornyl acetate **1b**	Bornyl benzoate **1c**	Borneol **1a**	Bornyl acetate **1b**	Bornyl benzoate **1c**
C-1	49.49	48.68	49.09	49.62	48.91	49.28	50.51	49.73	50.16
C-2	77.40	79.87	80.53	76.85	79.60	80.38	77.84	81.37	81.91
C-3	39.04	36.75	36.91	39.40	37.18	37.26	39.34	37.76	37.95
C-4	45.10	44.88	45.00	45.53	45.20	45.28	46.51	46.30	46.41
C-5	28.27	28.03	28.09	28.63	28.39	28.37	29.19	28.93	29.01
C-6	25.90	27.06	27.40	26.19	27.44	27.76	27.06	28.07	28.42
C-7	48.02	47.77	47.88	47.99	47.88	47.94	48.98	48.89	49.04
C-8	20.18	19.70	19.72	20.27	19.73	19.73	20.63	20.11	20.13
C-9	18.67	18.82	18.92	18.77	18.76	18.82	19.11	19.19	19.28
C-10	13.31	13.47	13.60	13.50	13.67	13.72	13.73	13.85	14.00
C-11		171.50	166.82		170.36	166.47		173.39	168.36
C-12		21.31			20.77			21.11	
C-13			130.91			131.63			131.94
C-14			129.51			129.84			130.42
C-15			128.33			128.56			129.66
C-16			132.74			132.70			134.24

Table S3. ^{1}H NMR chemical shifts (in ppm) for isborneol **2a**, isobornyl acetate **2b**, and isobornyl benzoate **2c** in different solvents

	$CDCl_3$ δ (^{1}H) ppm			C_6D_6 δ (^{1}H) ppm			CD_3OD δ (^{1}H) ppm		
Atom	Isoborneol **2a**	Isobornyl acetate **2b**	Isobornyl benzoate **2c**	Isoborneol **2a**	Isobornyl acetate **2b**	Isobornyl benzoate **2c**	Isoborneol **2a**	Isobornyl acetate **2b**	Isobornyl benzoate **2c**
H-2*endo*	3.612	4.654	4.923	3.299	4.835	5.082	3.573	4.649	4.908
H-3*endo*	1.709	1.745	1.907	1.565	1.719	1.798	1.675	1.762	1.842
H-3*exo*	1.737	1.793	1.925	1.774	1.740	1.853	1.714	1.799	1.916
H-4	1.702	1.716	1.805	1.587	1.528	1.553	1.651	1.726	1.792
H-5*endo*	0.980	1.079	1.151	0.874	0.848	0.880	0.990	1.140	1.186
H-5*exo*	1.665	1.689	1.752	1.545	1.503	1.517	1.701	1.744	1.779
H-6*endo*	0.975	1.147	1.249	0.794	0.972	1.007	0.971	1.140	1.233
H-6*exo*	1.488	1.536	1.612	1.369	1.355	1.371	1.496	1.598	1.656
H-8	0.814	0.828	0.893	0.762	0.710	0.720	0.827	0.865	0.916
H-9	1.011	0.969	1.131	1.132	1.001	1.101	1.002	1.012	1.146
H-10	0.888	0.833	0.937	0.803	0.857	0.897	0.884	0.847	0.929
H-12		2.013			1.672			2.000	
H-14*o*			8.016			8.190			7.976
H-15*m*			7.434			7.068			7.470
H-16*p*			7.547			7.100			7.590

Table S4. ^{13}C NMR chemical shifts (in ppm) for isborneol **2a**, isobornyl acetate **2b**, and isobornyl benzoate **2c** in different solvents

	$CDCl_3$ δ (^{13}C) ppm			C_6D_6 δ (^{13}C) ppm			CD_3OD δ (^{13}C) ppm		
Atom	Isoborneol **2a**	Isobornyl acetate **2b**	Isobornyl benzoate **2c**	Isoborneol **2a**	Isobornyl acetate **2b**	Isobornyl benzoate **2c**	Isoborneol **2a**	Isobornyl acetate **2b**	Isobornyl benzoate **2c**
C-1	48.94	48.59	49.01	49.02	48.80	49.18	49.93	49.72	50.15
C-2	79.91	80.95	81.57	79.60	80.67	81.43	80.34	82.54	83.03
C-3	40.38	38.75	38.92	40.88	38.98	39.07	41.19	39.69	39.84
C-4	45.03	45.02	45.12	45.41	45.32	45.38	46.49	46.48	46.55
C-5	27.22	27.02	27.08	27.63	27.26	27.26	28.21	27.97	28.01
C-6	33.90	33.73	33.76	34.17	33.97	33.90	35.16	34.85	34.83
C-7	46.33	46.91	47.03	46.49	47.04	47.11	47.29	47.95	48.06
C-8	20.46	20.10	20.08	20.66	20.20	20.17	21.02	20.57	20.57
C-9	20.09	19.84	20.13	20.39	20.09	20.23	20.64	20.40	20.64
C-10	11.29	11.35	11.59	11.44	11.54	11.69	11.88	11.79	12.00
C-11		170.70	166.06		169.57	165.73		172.58	167.58
C-12		21.30			20.78			21.18	
C-13			129.46			129.79			130.39
C-14			130.90			131.66			131.96
C-15			128.35			128.59			129.67
C-16			132.70			132.68			134.21

Table S5. ^{1}H NMR chemical shifts (in ppm) for borneol **1a** and its derivatives **1d**-**1g** in $CDCl_3$ and C_6D_6

	$CDCl_3$					C_6D_6				
	Borneol	Bornyl TBDMS	Bornyl DMMPS	Borneol TBDPS	Bornyl TPS	Borneol	Bornyl TBDMS	Bornyl DMMPS	Bornyl TBDPS	Bornyl TPS
	**1a**	**1d**	**1e**	**1f**	**1g**	**1a**	**1d**	**1e**	**1f**	**1g**
H-2*exo*	4.006	3.894	3.971	4.042	4.189	3.709	3.950	4.029	4.181	4.340
H-3*endo*	0.940	0.910	0.968	0.908	1.018	0.895	1.011	1.078	1.072	1.177
H-3*exo*	2.275	2.130	2.089	1.786	1.934	2.076	2.117	2.064	1.858	1.982
H-4*exo*	1.623	1.576	1.576	1.489	1.543	1.486	1.543	1.513	1.438	1.465
H-5*endo*	1.241	1.196	1.241	1.254	1.305	1.229	1.305	1.338	1.368	1.397
H-5*exo*	1.731	1.668	1.687	1.687	1.720	1.658	1.701	1.696	1.712	1.720
H-6*endo*	1.887	2.047	2.111	2.260	2.341	2.102	2.306	2.415	2.510	2.616
H-6*exo*	1.249	1.112	1.160	1.202	1.248	1.169	1.215	1.238	1.298	1.321
H-8	0.887	0.843	0.844	0.797	0.823	0.774	0.821	0.791	0.750	0.751
H-9	0.855	0.836	0.800	0.652	0.697	0.725	0.789	0.707	0.586	0.606
H-10	0.846	0.769	0.766	0.718	0.714	0.777	0.873	0.855	0.849	0.836
H-11		0.017	0.337				0.054	0.349		
H-13		0.891		1.089			0.996		1.225	
H-15*o*			7.590	7.650	7.630			7.633	7.778	7.789
H-16*m*			7.350	7.360	7.371			7.225	7.216	7.199
H-17*p*			7.375	7.406	7.428			7.218	7.206	7.190

Table S6. ^{13}C NMR chemical shifts (in ppm) for borneol **1a** and its derivatives **1d**-**1g** in $CDCl_3$ and C_6D_6

	$CDCl_3$					C_6D_6				
	Borneol **1a**	Bornyl TBDMS **1d**	Bornyl DMMPS **1e**	Borneol TBDPS **1f**	Bornyl TPS **1g**	Borneol **1a**	Bornyl TBDMS **1d**	Bornyl DMMPS **1e**	Bornyl TBDPS **1f**	Bornyl TPS **1g**
C-1	49.49	49.76	49.71	50.18	50.19	49.62	50.09	50.05	50.51	50.53
C-2	77.40	77.20	77.59	78.39	78.56	76.85	77.70	78.01	78.85	78.99
C-3	39.04	39.62	39.40	39.40	39.42	39.40	40.02	39.83	39.89	39.90
C-4	45.10	45.32	45.27	45.19	45.25	45.53	45.71	45.67	45.59	45.65
C-5	28.27	28.33	28.32	28.34	28.35	28.63	28.70	28.72	28.75	28.75
C-6	25.90	26.17	26.22	26.47	26.52	26.19	26.61	26.67	26.95	27.00
C-7	48.02	47.27	47.31	47.26	47.35	47.99	47.48	47.51	47.46	47.53
C-8	20.18	20.24	20.22	20.15	20.16	20.27	20.36	20.33	20.26	20.26
C-9	18.67	18.80	18.77	18.71	18.74	18.77	18.88	18.81	18.76	18.77
C-10	13.31	13.54	13.51	13.74	13.62	13.50	13.85	13.80	14.08	13.94
C-11		–4.49	–0.96				–4.32	–0.92		
C-11		–4.96	–0.98				–4.80			
C-12		18.09		19.34			18.34		19.60	
C-13		25.88		27.09			26.12		27.37	
C-14*ipso*			139.08	134.67	135.15			139.04	134.94	135.61
C-14*ipso*				135.15					135.41	
C-15*o*			133.47	135.99	135.54			133.86	136.38	136.00
C-16*m*			127.66	127.37	127.71			128.09	127.87	128.16
C-17*p*			129.27	129.36	129.80			129.71	129.84	130.19
C-17*p*				129.40					129.89	

Table S7. ^{1}H NMR chemical shifts of isoborneol **2a** and its derivatives **2d**-**2g** in $CDCl_3$ and C_6D_6

	$CDCl_3$					C_6D_6				
	Isoborneol	Isobornyl TBDMS	Isobornyl DMMPS	Isobornyl TBDPS	Isobornyl TPS	Isoborneol	Isobornyl TBDMS	Isobornyl DMMPS	Isobornyl TBDPS	Isobornyl TPS
	**2a**	**2d**	**2e**	**2f**	**2g**	**2a**	**2d**	**2e**	**2f**	**2g**
H-2*exo*	3.612	3.508	3.582	3.704	3.868	3.299	3.487	3.576	3.804	3.966
H-3*endo*	1.709	1.603	1.605	1.404	1.518	1.565	1.559	1.527	1.437	1.521
H-3*exo*	1.737	1.698	1.760	1.751	1.845	1.774	1.759	1.836	1.871	1.962
H-4*exo*	1.702	1.648	1.654	1.607	1.655	1.587	1.629	1.608	1.558	1.575
H-5*endo*	0.980	0.994	0.941	0.838	0.888	0.874	0.915	0.844	0.708	0.739
H-5*exo*	1.665	1.634	1.632	1.542	1.598	1.545	1.609	1.565	1.489	1.506
H-6*endo*	0.975	0.943	0.894	0.736	0.788	0.794	0.874	0.790	0.664	0.738
H-6*exo*	1.488	1.470	1.460	1.357	1.404	1.369	1.442	1.396	1.297	1.322
H-8	0.814	0.791	0.801	0.783	0.815	0.762	0.808	0.796	0.766	0.771
H-9	1.011	1.002	1.051	1.147	1.211	1.132	1.166	1.230	1.301	1.356
H-10	0.898	0.814	0.839	0.772	0.801	0.803	0.928	0.960	0.912	0.929
H-11		0.003	0.318				0.031	0.326		
H-11							0.042			
H-13		0.871		1.082			0.973		1.213	
H-15*o*			7.574	7.654	7.626			7.622	7.762	7.774
H-16*m*			7.371	7.360	7.373			7.230	7.220	7.203
H-17*p*			7.360	7.416	7.432			7.248	7.211	7.203

Table S8. ^{13}C NMR chemical shifts of isoborneol **2a** and its derivatives **2d**-**2g** in $CDCl_3$ and C_6D_6

	$CDCl_3$					C_6D_6				
	Isoborneol	Isobornyl TBDMS	Isobornyl DMMPS	Isobornyl TBDPS	Isobornyl TPS	Isoborneol	Isobornyl TBDMS	Isobornyl DMMPS	Isobornyl TBDPS	Isobornyl TPS
	**2a**	**2d**	**2e**	**2f**	**2g**	**2a**	**2d**	**2e**	**2f**	**2g**
C-1	48.94	49.10	49.05	49.51	49.52	49.02	49.38	49.31	49.82	49.82
C-2	79.91	79.68	79.91	80.40	80.63	79.60	80.09	80.29	80.77	81.00
C-3	40.38	42.12	42.07	42.24	42.13	40.88	42.44	42.39	42.65	42.53
C-4	45.03	45.25	45.27	45.17	45.24	45.41	45.63	45.65	45.55	45.61
C-5	27.22	27.35	27.26	27.09	27.13	27.63	27.67	27.56	27.41	27.43
C-6	33.91	33.87	33.87	33.77	33.75	34.17	34.15	34.13	34.06	34.02
C-7	46.33	46.49	46.57	46.64	46.70	46.49	46.77	46.85	46.91	46.96
C-8	20.46	20.56	20.20	20.48	20.47	20.66	20.74	20.55	20.65	20.64
C-9	20.09	20.12	20.53	20.39	20.47	20.39	20.46	20.71	20.73	20.78
C-10	11.29	11.98	11.98	12.29	12.23	11.44	12.30	12.30	12.66	12.58
C-11		–4.61	–0.96				–4.44	–0.92		
C-11		–5.09	–1.03				–4.95	–0.95		
C-12		17.94		19.16			18.19		19.40	
C-13		25.80		27.09			25.93		27.36	
C-14*ipso*			139.06	134.61	135.06			139.01	134.90	135.52
C-14*ipso*				134.88					135.17	
C-15*o*			133.47	136.14	135.59			133.86	136.52	136.04
C-15*o*				136.15					136.55	
C-16*m*			127.65	127.36	127.70			127.91	127.91	128.15
C-17*p*			129.23	129.37	129.78			129.70	129.85	130.18
C-17*p*				129.40					129.86	

Table S9. Optimized coordinates, energies, and calculated NMR chemical shifts of borneol **1a**

Optimized coordinates in the gas phase

Calculation Method	RB3LYP
Basis Set	6-311+G(2d,p)
Charge	0
Spin	Singlet
E(RB3LYP)	-467.26130141 a.u.

Zero-point correction= 0.263666 (Hartree/Particle)
Thermal correction to Energy= 0.274813
Thermal correction to Enthalpy= 0.275757
Thermal correction to Gibbs Free Energy= 0.228800
Sum of electronic and zero-point Energies= -466.997635
Sum of electronic and thermal Energies= -466.986488
Sum of electronic and thermal Enthalpies= -466.985544
Sum of electronic and thermal Free Energies= -467.032501

Standard orientation:

Center Number	Atomic Number	Atomic Type	Coordinates (Angstroms) X	Y	Z
1	6	0	-0.462787	-1.407116	0.331034
2	6	0	0.842451	-1.178354	1.122401
3	6	0	1.275886	0.246297	0.697853
4	6	0	0.198747	0.659279	-0.348796
5	6	0	-0.059628	-1.645096	-1.139005
6	6	0	0.423527	-0.236423	-1.593994
7	6	0	-1.091593	0.019333	0.288168
8	6	0	-2.344789	0.124753	-0.594562
9	6	0	-1.481889	0.569267	1.671243
10	6	0	0.176033	2.149323	-0.649795
11	8	0	2.619878	0.215946	0.209400
12	1	0	-1.104489	-2.185323	0.749035
13	1	0	0.694610	-1.245733	2.201159
14	1	0	1.617596	-1.898188	0.855642
15	1	0	1.239350	0.935341	1.547279
16	1	0	-0.902856	-1.994938	-1.736962

17	1	0	0.724951	-2.399337	-1.220119
18	1	0	1.472779	-0.232723	-1.886786
19	1	0	-0.152264	0.134601	-2.444031
20	1	0	-3.147166	-0.492948	-0.180654
21	1	0	-2.712691	1.154139	-0.619593
22	1	0	-2.185645	-0.191255	-1.624029
23	1	0	-1.788951	1.616268	1.598736
24	1	0	-2.336844	0.011558	2.063916
25	1	0	-0.689723	0.510165	2.415894
26	1	0	-0.616826	2.403889	-1.357685
27	1	0	0.022277	2.744683	0.255009
28	1	0	1.118819	2.473721	-1.102572
29	1	0	2.932298	1.120937	0.103279

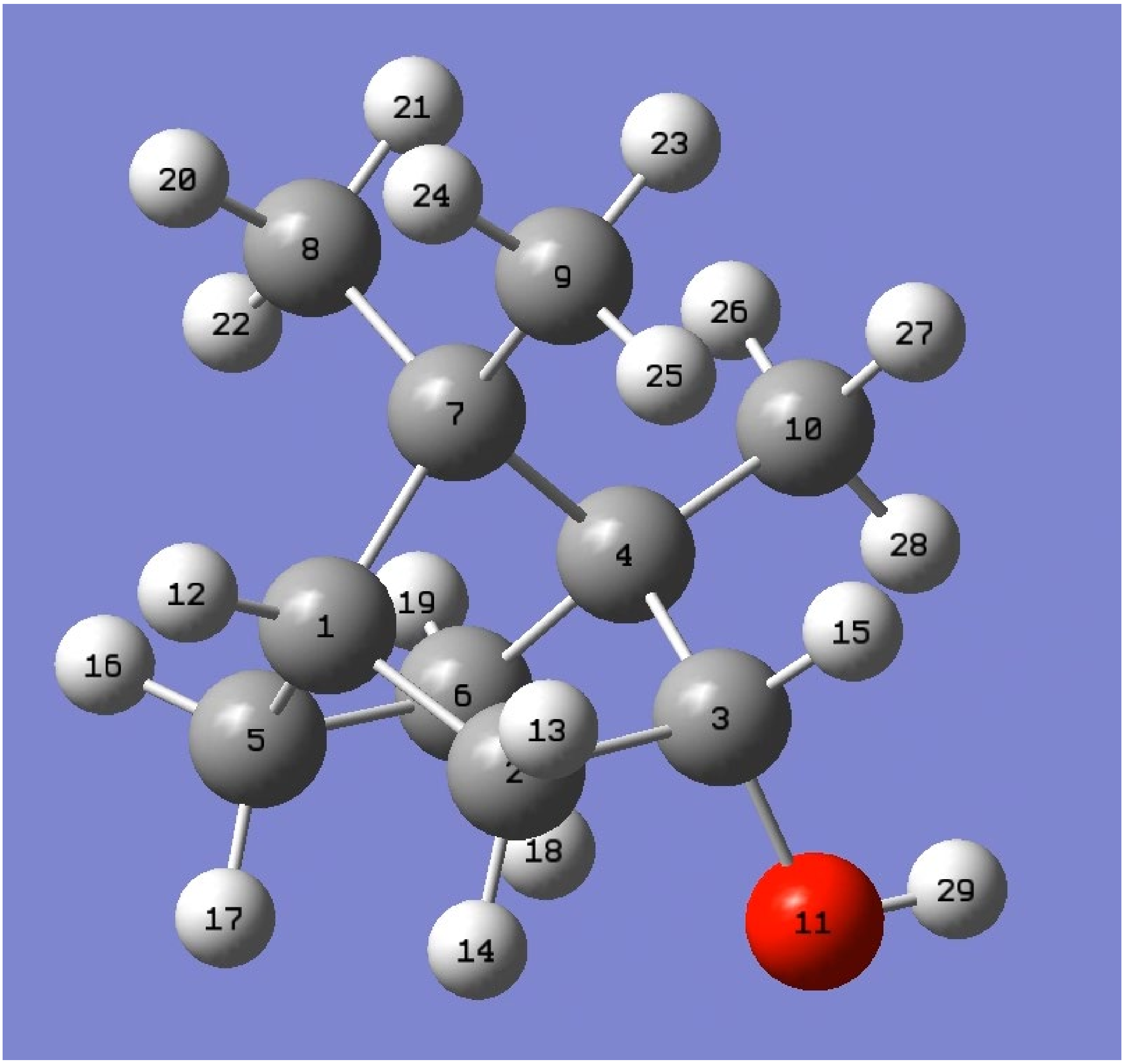

Compound borneol **1a**

	In the gas phase	In chloroform	In benzene	In methanol
**Energy**	E(RB3LYP) -467.26130141 a.u.	E(RB3LYP) -467.26447765 a.u.	E(RB3LYP) -467.26324182 a.u.	E(RB3LYP) -467.26582019 a.u.
	E(RmPW1PW91) -467.15784005 a.u.	E(RmPW1PW91) -467.16107530 a.u.	E(RmPW1PW91) -467.15981723 a.u.	E(RmPW1PW91) -467.16244061 a.u.
	E(RwB97XD) -467.13289377 a.u.	E(RwB97XD) -467.13619181 a.u.	E(RwB97XD) -467.13490887 a.u.	E(RwB97XD) -467.13758494 a.u.

Reference: TMS B3LYP/6-311+G(2d,p) GIAO

Reference shielding: 31.8821 ppm

^{1}H NMR chemical shifts (ppm), GIAO//6-311+G(2d,p)

Atom	Atom	In the gas phase			In chloroform			In benzene			In methanol		
label	Assignment	B3LYP	mPW1PW91	wB97XD	B3LYP	mPW1PW91	wB97XD	B3LYP	mPW1PW91	wB97XD	B3LYP	mPW1PW91	wB97XD
12-H	4-exo	1.4149	1.5388	1.4319	1.4496	1.5729	1.4666	1.4355	1.5592	1.4526	1.4659	1.5886	1.4827
13-H	3-exo	2.1933	2.2637	2.1467	2.2501	2.3208	2.2056	2.2264	2.2970	2.1810	2.2789	2.3497	2.2356
14-H	3-endo	1.0188	1.1259	1.0397	0.8963	1.0059	0.9171	0.9512	1.0597	0.9720	0.8257	0.9364	0.8462
15-H	2-exo	4.0815	4.1552	3.9977	4.1572	4.2322	4.0765	4.1242	4.1987	4.0422	4.1991	4.2745	4.1199
16-H	5-exo	1.6840	1.7646	1.6522	1.7115	1.7930	1.6810	1.6987	1.7799	1.6677	1.7290	1.8110	1.6993
17-H	5-endo	1.2044	1.3003	1.2090	1.1651	1.2632	1.1711	1.1855	1.2827	1.1909	1.1348	1.2337	1.1412
18-H	6-endo	2.1900	2.2713	2.1755	2.0817	2.1633	2.0654	2.1262	2.2077	2.1106	2.0295	2.1112	2.0123
19-H	6-exo	1.0966	1.1899	1.0900	1.1464	1.2403	1.1404	1.1254	1.2190	1.1190	1.1718	1.2662	1.1664
20-H	8-CH3	0.6419	0.7451	0.6905	0.6524	0.7582	0.7041	0.6487	0.7535	0.6993	0.6557	0.7623	0.7086
21-H	8-CH3	0.6816	0.7774	0.7125	0.7071	0.8052	0.7401	0.6964	0.7936	0.7286	0.7196	0.8187	0.7534
22-H	8-CH3	1.0821	1.1619	1.0634	1.0741	1.1544	1.0547	1.0772	1.1571	1.0579	1.0709	1.1517	1.0514
23-H	9-CH3	0.6770	0.7747	0.7050	0.7083	0.8083	0.7381	0.6950	0.7940	0.7241	0.7245	0.8256	0.7551
24-H	9-CH3	0.6962	0.8015	0.7327	0.7050	0.8123	0.7442	0.7025	0.8090	0.7407	0.7059	0.8139	0.7462
25-H	9-CH3	0.9790	1.0620	0.9535	0.9821	1.0655	0.9569	0.9799	1.0630	0.9544	0.9865	1.0703	0.9617
26-H	10-CH3	0.6765	0.7773	0.7152	0.6726	0.7745	0.7119	0.6757	0.7770	0.7148	0.6668	0.7692	0.7063
27-H	10-CH3	0.5870	0.6905	0.6259	0.6332	0.7379	0.6737	0.6123	0.7165	0.6521	0.6611	0.7663	0.7024
28-H	10-CH3	0.7964	0.8954	0.8259	0.8564	0.9586	0.8909	0.8324	0.9334	0.8649	0.8824	0.9859	0.9191
29-H	OH	0.1408	0.2329	0.1865	0.5452	0.6377	0.6006	0.3884	0.4808	0.4400	0.7150	0.8074	0.7746

Reference: TMS B3LYP/6-311+G(2d,p) GIAO

Reference shielding: 182.466 ppm

^{13}C NMR chemical shifts (ppm), GIAO//6-311+G(2d,p)

Atom	Atom	In the gas phase			In chloroform			In benzene			In methanol		
label	Assignment	B3LYP	mPW1PW91	wB97XD	B3LYP	mPW1PW91	wB97XD	B3LYP	mPW1PW91	wB97XD	B3LYP	mPW1PW91	wB97XD
1-C	4	50.0846	44.2924	42.6127	50.0029	44.1968	42.5202	50.0356	44.2349	42.5570	49.9670	44.1550	42.4802
2-C	5	39.9509	34.2421	33.4753	40.0867	34.3416	33.6043	40.0167	34.2871	33.5374	40.1952	34.4313	33.7094
3-C	1	83.3212	76.8064	75.5709	83.0870	76.5936	75.3743	83.1926	76.6899	75.4643	82.9493	76.4677	75.2551
4-C	2	55.2818	49.7080	48.7422	55.4390	49.8663	48.8902	55.3731	49.7998	48.8278	55.5174	49.9454	48.9646
5-C	6	30.7004	25.4675	24.5819	30.6107	25.3511	24.4744	30.6356	25.3869	24.5063	30.6030	25.3305	24.4588
6-C	7	28.5956	23.2801	22.4507	28.4537	23.1191	22.3015	28.5128	23.1859	22.3633	28.3839	23.0403	22.2287
7-C	3	55.2032	49.4133	48.0603	55.2701	49.4781	48.1175	55.2441	49.4531	48.0957	55.2978	49.5044	48.1397
8-C	8	20.8149	16.0936	15.5625	20.5210	15.7948	15.2623	20.6368	15.9120	15.3802	20.3950	15.6682	15.1347
9-C	9	18.9996	14.3367	13.8878	18.7134	14.0438	13.5954	18.8241	14.1568	13.7083	18.5974	13.9259	13.4774
10-C	10	13.4869	9.0661	8.6297	13.1540	8.7295	8.2956	13.2863	8.8627	8.4277	13.0080	8.5835	8.1508

Table S10. Optimized coordinates, energies, and calculated NMR chemical shifts of isoborneol **2a**

Optimized coordinates in the gas phase

Calculation Method	RB3LYP
Basis Set	6-311+G(2d,p)
Charge	0
Spin	Singlet
E(RB3LYP)	-467.26123664 a.u.

Zero-point correction=	0.263715 (Hartree/Particle)
Thermal correction to Energy=	0.274809
Thermal correction to Enthalpy=	0.275754
Thermal correction to Gibbs Free Energy=	0.228962
Sum of electronic and zero-point Energies=	-466.997522
Sum of electronic and thermal Energies=	-466.986427
Sum of electronic and thermal Enthalpies=	-466.985483
Sum of electronic and thermal Free Energies=	-467.032274

Standard orientation:

Center Number	Atomic Number	Atomic Type	Coordinates (Angstroms) X	Y	Z
1	6	0	-0.203351	0.205767	0.762611
2	6	0	0.697426	-0.840365	1.475214
3	6	0	1.381270	-1.609977	0.305936
4	6	0	0.788143	-0.921602	-0.942660
5	6	0	0.669640	0.566370	-0.496546
6	6	0	-1.360258	-0.649256	0.154643
7	6	0	-0.685504	-1.364166	-1.041305
8	6	0	-0.012006	1.477994	-1.530787
9	6	0	2.020962	1.219985	-0.160139
10	6	0	-0.663023	1.341057	1.661245
11	8	0	-2.492293	0.084265	-0.325342
12	1	0	0.108501	-1.496021	2.121275
13	1	0	1.430293	-0.346773	2.115550
14	1	0	2.467305	-1.515627	0.344813
15	1	0	1.154879	-2.677921	0.319242

16	1	0	1.359405	-1.090208	-1.857395
17	1	0	-1.713361	-1.364901	0.905798
18	1	0	-0.795566	-2.449022	-0.993660
19	1	0	-1.157217	-1.029287	-1.965042
20	1	0	0.603919	1.536007	-2.433402
21	1	0	-1.008836	1.153247	-1.813754
22	1	0	-0.102318	2.495340	-1.139763
23	1	0	1.871728	2.229748	0.231420
24	1	0	2.613808	0.669992	0.568920
25	1	0	2.623632	1.315354	-1.067822
26	1	0	-1.278719	2.063273	1.120336
27	1	0	-1.250120	0.962898	2.505425
28	1	0	0.188793	1.881146	2.081779
29	1	0	-2.977580	0.433804	0.429744

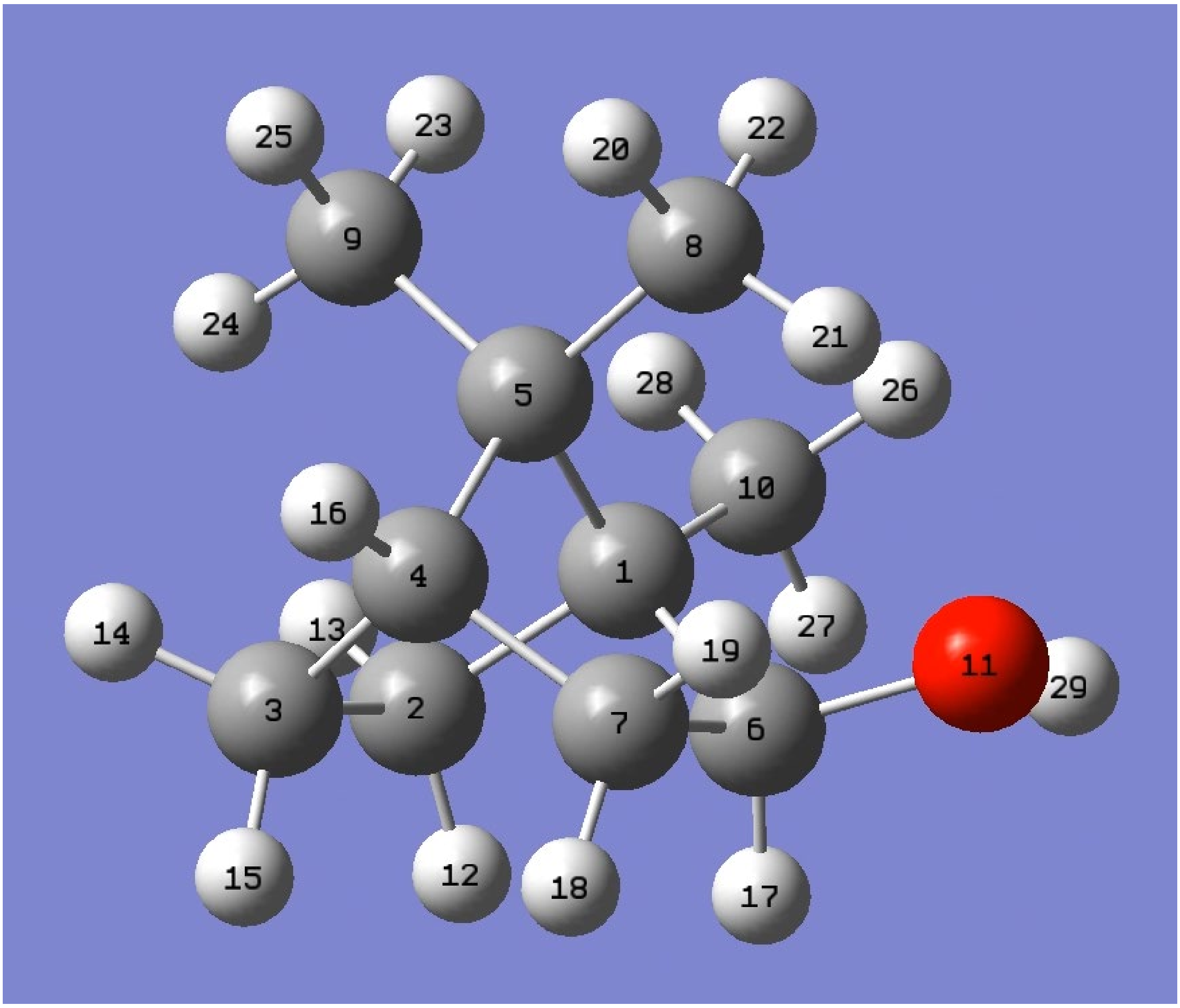

Compound isoborneol **2a**

	In the gas phase	In chloroform	In benzene	In methanol
**Energy**	E(RB3LYP) -467.26123664 a.u.	E(RB3LYP) -467.26413885 a.u.	E(RB3LYP) -467.26299884 a.u.	E(RB3LYP) -467.26539068 a.u.
	E(RmPW1PW91) -467.15779795 a.u.	E(RmPW1PW91) -467.16075587 a.u.	E(RmPW1PW91) -467.15959580 a.u.	E(RmPW1PW91) -467.16202557 a.u.
	E(RwB97XD) -467.13282062 a.u.	E(RwB97XD) -467.13583827 a.u.	E(RwB97XD) -467.13465407 a.u.	E(RwB97XD) -467.13713562 a.u.

Reference: TMS B3LYP/6-311+G(2d,p) GIAO

Reference shielding: 31.8821 ppm

^{1}H NMR chemical shifts (ppm), GIAO//6-311+G(2d,p)

Atom	Atom	In the gas phase			In chloroform			In benzene			In methanol		
label	Assignment	B3LYP	mPW1PW91	wB97XD	B3LYP	mPW1PW91	wB97XD	B3LYP	mPW1PW91	wB97XD	B3LYP	mPW1PW91	wB97XD
12-H	6-endo	0.7870	0.8786	0.8053	0.8346	0.9275	0.8550	0.8133	0.9058	0.8330	0.8623	0.9555	0.8836
13-H	6-exo	1.4597	1.5454	1.4292	1.4797	1.5671	1.4507	1.4720	1.5586	1.4424	1.4876	1.5758	1.4592
14-H	5-exo	1.6807	1.7589	1.6428	1.6854	1.7655	1.6499	1.6839	1.7632	1.6474	1.6860	1.7671	1.6517
15-H	5-endo	0.9212	1.0179	0.9391	0.9375	1.0363	0.9581	0.9305	1.0286	0.9500	0.9463	1.0459	0.9680
16-H	4-exo	1.4847	1.6155	1.5127	1.5121	1.6421	1.5391	1.5020	1.6325	1.5295	1.5218	1.6513	1.5482
17-H	2-endo	3.6473	3.7122	3.5922	3.7235	3.7900	3.6722	3.6904	3.7563	3.6376	3.7652	3.8321	3.7156
18-H	3-endo	1.5739	1.6693	1.5851	1.6204	1.7174	1.6351	1.6015	1.6979	1.6148	1.6423	1.7397	1.6585
19-H	3-exo	1.7840	1.8858	1.7755	1.6840	1.7867	1.6731	1.7285	1.8307	1.7186	1.6276	1.7312	1.6159
20-H	9-CH3	0.6328	0.7369	0.6700	0.6457	0.7521	0.6856	0.6396	0.7451	0.6783	0.6541	0.7614	0.6952
21-H	9-CH3	1.8730	1.9491	1.8717	1.7588	1.8346	1.7538	1.8069	1.8827	1.8034	1.7019	1.7778	1.6953
22-H	9-CH3	0.6240	0.7262	0.6581	0.6435	0.7477	0.6787	0.6355	0.7389	0.6703	0.6522	0.7573	0.6878
23-H	8-CH3	0.6982	0.7932	0.7298	0.7077	0.8051	0.7407	0.7037	0.8001	0.7362	0.7122	0.8107	0.7458
24-H	8-CH3	0.8743	0.9493	0.8592	0.9048	0.9804	0.8910	0.8915	0.9668	0.8771	0.9213	0.9973	0.9082
25-H	8-CH3	0.6922	0.7964	0.7447	0.6809	0.7869	0.7355	0.6861	0.7914	0.7399	0.6740	0.7808	0.7296
26-H	10-CH3	1.0115	1.0987	1.0199	0.9969	1.0862	1.0055	1.0054	1.0938	1.0139	0.9821	1.0725	0.9907
27-H	10-CH3	0.5633	0.6753	0.6096	0.6571	0.7703	0.7072	0.6162	0.7290	0.6648	0.7085	0.8220	0.7604
28-H	10-CH3	0.7053	0.8113	0.7409	0.7112	0.8187	0.7477	0.7100	0.8169	0.7462	0.7107	0.8189	0.7475
29-H	OH	0.6247	0.7057	0.6237	0.9922	1.0721	0.9998	0.8489	0.9294	0.8532	1.1476	1.2267	1.1587

Reference: TMS B3LYP/6-311+G(2d,p) GIAO

Reference shielding: 182.466 ppm

^{13}C NMR chemical shifts (ppm), GIAO//6-311+G(2d,p)

Atom	Atom	In the gas phase			In chloroform			In benzene			In methanol		
label	Assignment	B3LYP	mPW1PW91	wB97XD	B3LYP	mPW1PW91	wB97XD	B3LYP	mPW1PW91	wB97XD	B3LYP	mPW1PW91	wB97XD
1-C	1	54.7816	49.1983	48.2959	54.9910	49.4032	48.4845	54.9070	49.3208	48.4087	55.0847	49.4952	48.5690
2-C	6	36.8832	31.1871	30.2613	36.5395	30.8350	29.9050	36.6828	30.9807	30.0527	36.3705	30.6652	29.7321
3-C	5	30.2141	24.9485	24.0060	29.9115	24.6323	23.6928	30.0349	24.7604	23.8200	29.7703	24.4870	23.5481
4-C	4	50.0491	44.2936	42.6334	50.0020	44.2354	42.5774	50.0189	44.2568	42.5979	49.9868	44.2152	42.5579
5-C	7	52.7821	47.2374	46.1912	52.7949	47.2465	46.2006	52.7882	47.2416	46.1955	52.8053	47.2540	46.2084
6-C	2	86.6712	79.9810	78.6174	86.4082	79.7352	78.3809	86.5209	79.8404	78.4826	86.2721	79.6087	78.2576
7-C	3	41.6878	36.1152	35.2703	41.5641	35.9715	35.1528	41.6117	36.0272	35.1978	41.5143	35.9121	35.1058
8-C	9	20.4516	15.7307	15.2557	20.1889	15.4615	14.9886	20.2962	15.5709	15.0971	20.0676	15.3385	14.8666
9-C	8	21.0148	16.2693	15.8146	20.6823	15.9311	15.4748	20.8142	16.0649	15.6093	20.5387	15.7859	15.3284
10-C	10	11.2543	6.8739	6.4506	10.9349	6.5587	6.1405	11.0616	6.6829	6.2627	10.7942	6.4224	6.0064

Table S11. Optimized coordinates, energies, and calculated NMR chemical shifts of bornyl acetate **1b**

Optimized coordinates in the gas phase

Calculation Method	RB3LYP
Basis Set	6-311+G(2d,p)
Charge	0
Spin	Singlet
E(RB3LYP)	-619.97330510 a.u.

Zero-point correction= 0.301245 (Hartree/Particle)
Thermal correction to Energy= 0.315866
Thermal correction to Enthalpy= 0.316810
Thermal correction to Gibbs Free Energy= 0.260096
Sum of electronic and zero-point Energies= -619.672060
Sum of electronic and thermal Energies= -619.657439
Sum of electronic and thermal Enthalpies= -619.656495
Sum of electronic and thermal Free Energies= -619.713209

Standard orientation:

Center Number	Atomic Number	Atomic Type	Coordinates (Angstroms) X	Y	Z
1	6	0	0.695408	-0.343619	0.695244
2	6	0	0.785020	-1.730718	0.004112
3	6	0	1.280500	-1.406705	-1.436258
4	6	0	1.388548	0.132400	-1.421642
5	6	0	-0.499904	0.407241	0.065593
6	6	0	-0.052202	0.686233	-1.392831
7	6	0	1.895003	0.428833	0.023124
8	6	0	1.976059	1.927734	0.360137
9	6	0	3.275242	-0.167947	0.336961
10	6	0	0.649564	-0.404520	2.213469
11	1	0	-0.709957	1.330963	0.600132
12	8	0	-1.700080	-0.400997	0.156524
13	6	0	-2.878808	0.241983	0.022598
14	8	0	-2.981234	1.426546	-0.182441
15	6	0	-4.035080	-0.714506	0.164908
16	1	0	-0.178318	-2.238270	0.013822

17	1	0	1.487896	-2.372966	0.537370
18	1	0	2.242559	-1.876735	-1.646147
19	1	0	0.584479	-1.750374	-2.203729
20	1	0	2.003771	0.543933	-2.223936
21	1	0	-0.701165	0.175231	-2.105700
22	1	0	-0.101639	1.751049	-1.618932
23	1	0	2.213079	2.072981	1.417408
24	1	0	2.780433	2.393618	-0.215795
25	1	0	1.064693	2.484907	0.148963
26	1	0	3.551882	0.028110	1.376220
27	1	0	3.337892	-1.242979	0.178205
28	1	0	4.035926	0.302420	-0.292501
29	1	0	-0.231587	-0.956092	2.551316
30	1	0	1.528696	-0.912584	2.617394
31	1	0	0.602954	0.593904	2.656648
32	1	0	-4.004784	-1.193676	1.145293
33	1	0	-4.970129	-0.173243	0.045284
34	1	0	-3.962276	-1.503042	-0.586469

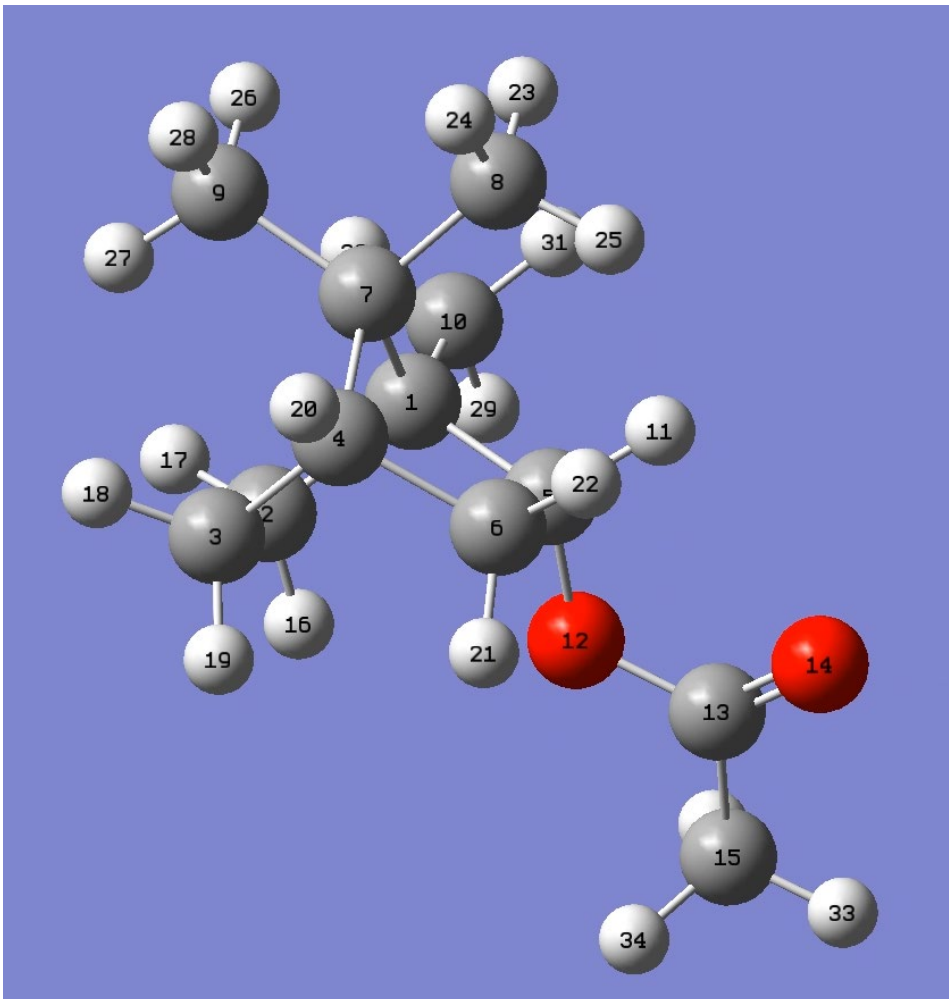

Compound bornyl acetate **1b**

	In the gas phase	In chloroform	In benzene	In methanol
**Energy**	E(RB3LYP) -619.97330510 a.u.	E(RB3LYP) -619.97747791 a.u.	E(RB3LYP) -619.97593774 a.u.	E(RB3LYP) -619.97904138 a.u.
	E(RmPW1PW91) -619.82943678 a.u.	E(RmPW1PW91) -619.83366049 a.u.	E(RmPW1PW91) -619.83210268 a.u.	E(RmPW1PW91) -619.83523953 a.u.
	E(RwB97XD) -619.79519947 a.u.	E(RwB97XD) -619.79949178 a.u.	E(RwB97XD) -619.79790802 a.u.	E(RwB97XD) -619.80109811 a.u.

Reference: TMS B3LYP/6-311+G(2d,p) GIAO

Reference shielding: 31.8821 ppm

^{1}H NMR chemical shifts (ppm), GIAO//6-311+G(2d,p)

Atom	Atom	In the gas phase			In chloroform			In benzene			In methanol		
label	Assignment	B3LYP	mPW1PW91	wB97XD	B3LYP	mPW1PW91	wB97XD	B3LYP	mPW1PW91	wB97XD	B3LYP	mPW1PW91	wB97XD
11-H	2-exo	4.9113	5.0141	4.8739	4.8926	4.9944	4.8528	4.9011	5.0033	4.8623	4.8814	4.9829	4.8406
16-H	6-endo	2.0310	2.1198	2.0221	1.9637	2.0531	1.9530	1.9890	2.0781	1.9790	1.9376	2.0272	1.9260
17-H	6-exo	1.2919	1.3839	1.2671	1.3517	1.4459	1.3284	1.3276	1.4208	1.3036	1.3787	1.4741	1.3564
18-H	5-exo	1.7361	1.8180	1.7137	1.7652	1.8496	1.7459	1.7536	1.8370	1.7330	1.7778	1.8636	1.7602
19-H	5-endo	1.1385	1.2355	1.1374	1.1505	1.2498	1.1518	1.1454	1.2439	1.1458	1.1571	1.2571	1.1592
20-H	4-exo	1.4682	1.5952	1.5082	1.5221	1.6493	1.5631	1.5009	1.6282	1.5416	1.5453	1.6724	1.5865
21-H	3-endo	0.7834	0.8979	0.8041	0.8042	0.9183	0.8244	0.7941	0.9085	0.8145	0.8191	0.9325	0.8388
22-H	3-exo	2.4492	2.5216	2.4052	2.4305	2.5046	2.3863	2.4433	2.5167	2.3993	2.4084	2.4835	2.3642
23-H	9-CH3	0.7406	0.8368	0.7695	0.7679	0.8668	0.7989	0.7571	0.8548	0.7871	0.7799	0.8801	0.8119
24-H	9-CH3	0.7310	0.8344	0.7730	0.7572	0.8635	0.8026	0.7472	0.8523	0.7912	0.7682	0.8756	0.8149
25-H	9-CH3	1.0090	1.0926	0.9993	0.9795	1.0628	0.9680	0.9938	1.0771	0.9830	0.9600	1.0433	0.9476
26-H	8-CH3	0.7232	0.8205	0.7603	0.7424	0.8421	0.7815	0.7352	0.8339	0.7734	0.7498	0.8508	0.7899
27-H	8-CH3	1.0650	1.1468	1.0664	1.0765	1.1592	1.0782	1.0706	1.1529	1.0721	1.0848	1.1682	1.0869
28-H	8-CH3	0.6733	0.7778	0.7305	0.6955	0.8029	0.7562	0.6874	0.7937	0.7468	0.7036	0.8121	0.7657
29-H	10-CH3	1.0150	1.1203	1.0521	0.9788	1.0852	1.0178	0.9930	1.0991	1.0313	0.9635	1.0700	1.0027
30-H	10-CH3	0.6022	0.7025	0.6448	0.6477	0.7508	0.6931	0.6291	0.7309	0.6732	0.6691	0.7738	0.7160
31-H	10-CH3	0.6493	0.7539	0.6842	0.6823	0.7879	0.7184	0.6703	0.7755	0.7059	0.6940	0.8001	0.7307
32-H	12-CH3	2.0619	2.1485	2.0644	2.2009	2.2922	2.2122	2.1481	2.2378	2.1563	2.2555	2.3484	2.2697
33-H	12-CH3	1.6669	1.7938	1.7281	1.6698	1.8015	1.7372	1.6727	1.8025	1.7376	1.6620	1.7959	1.7321
34-H	12-CH3	2.0057	2.0950	2.0220	2.1508	2.2446	2.1748	2.0946	2.1868	2.1159	2.2112	2.3065	2.2376

Reference: TMS B3LYP/6-311+G(2d,p) GIAO

Reference shielding: 182.466 ppm

^{13}C NMR chemical shifts (ppm), GIAO//6-311+G(2d,p)

Atom	Atom	In the gas phase			In chloroform			In benzene			In methanol		
label	Assignment	B3LYP	mPW1PW91	wB97XD	B3LYP	mPW1PW91	wB97XD	B3LYP	mPW1PW91	wB97XD	B3LYP	mPW1PW91	wB97XD
1-C	7	54.1014	48.6176	47.5698	54.2251	48.7387	47.6886	54.1762	48.6910	47.6418	54.2791	48.7913	47.7401
2-C	6	30.0982	24.6903	23.8801	29.9697	24.5458	23.7491	30.0217	24.6041	23.8021	29.9124	24.4819	23.6908
3-C	5	30.6021	25.3385	24.4984	30.3603	25.0834	24.2481	30.4577	25.1855	24.3484	30.2504	24.9698	24.1361
4-C	4	49.9324	44.1847	42.6088	49.8596	44.1040	42.5274	49.8878	44.1350	42.5586	49.8305	44.0722	42.4955
5-C	2	84.5261	78.3691	77.3173	84.8486	78.6752	77.6225	84.7338	78.5669	77.5143	84.9573	78.7768	77.7244
6-C	3	40.2592	34.5038	33.7604	39.9540	34.2019	33.4562	40.0822	34.3279	33.5829	39.7965	34.0486	33.3025
7-C	1	54.4612	48.7192	47.4207	54.5888	48.8491	47.5386	54.5395	48.7988	47.4930	54.6425	48.9035	47.5879
8-C	9	19.2004	14.5036	14.0499	18.9235	14.2204	13.7712	19.0317	14.3307	13.8797	18.8073	14.1023	13.6553
9-C	8	20.1631	15.4557	15.0189	19.8593	15.1472	14.7098	19.9792	15.2686	14.8316	19.7287	15.0154	14.5772
10-C	10	13.8904	9.4495	8.9747	13.6663	9.2157	8.7455	13.7513	9.3042	8.8321	13.5792	9.1249	8.6571
13-C	11	176.5177	171.3801	171.2573	179.1693	173.9973	173.9396	178.1877	173.0293	172.9479	180.1678	174.9812	174.9469
15-C	12	21.1795	17.0766	16.9970	21.6825	17.5487	17.4720	21.4805	17.3591	17.2811	21.9054	17.7581	17.6830

Table S12. Optimized coordinates, energies, and calculated NMR chemical shifts of bornyl benzoate **1c**

Optimized coordinates in the gas phase

Calculation Method	RB3LYP
Basis Set	6-311+G(2d,p)
Charge	0
Spin	Singlet
E(RB3LYP)	-811.76185551 a.u.
RMS Gradient Norm	0.00000270 a.u.

Zero-point correction= 0.354647 (Hartree/Particle)
Thermal correction to Energy= 0.372068
Thermal correction to Enthalpy= 0.373012
Thermal correction to Gibbs Free Energy= 0.309110
Sum of electronic and zero-point Energies= -811.407208
Sum of electronic and thermal Energies= -811.389788
Sum of electronic and thermal Enthalpies= -811.388843
Sum of electronic and thermal Free Energies= -811.452746

Standard orientation:

Center Number	Atomic Number	Atomic Type	Coordinates (Angstroms) X	Y	Z
1	6	0	1.909030	-0.469345	0.615494
2	6	0	1.754822	-1.691078	-0.329952
3	6	0	2.356956	-1.206067	-1.681642
4	6	0	2.756825	0.251464	-1.372340
5	6	0	3.258472	0.165737	0.101576
6	6	0	0.905894	0.607471	0.144579
7	6	0	1.448466	1.059780	-1.236228
8	6	0	3.614498	1.528992	0.719337
9	6	0	4.485583	-0.738177	0.294238
10	6	0	1.798708	-0.805522	2.093953
11	8	0	-0.432101	0.052971	0.087315
12	6	0	-1.448683	0.938053	0.062896
13	8	0	-1.290853	2.138029	0.062677
14	6	0	-2.785864	0.276571	0.037796
15	1	0	0.863467	1.438064	0.845434

16	6	0	-3.918148	1.093129	-0.006006
17	6	0	-5.185822	0.528386	-0.029527
18	6	0	-5.332028	-0.856272	-0.009163
19	6	0	-4.206889	-1.674337	0.034784
20	6	0	-2.936484	-1.112048	0.058183
21	1	0	0.711975	-1.991548	-0.419324
22	1	0	2.298649	-2.548581	0.069777
23	1	0	3.218601	-1.807043	-1.976519
24	1	0	1.637457	-1.257161	-2.500847
25	1	0	3.467800	0.678896	-2.081784
26	1	0	1.610842	2.136992	-1.256701
27	1	0	0.738499	0.826090	-2.030921
28	1	0	3.831468	1.424317	1.785652
29	1	0	4.516968	1.925228	0.245751
30	1	0	2.838114	2.285219	0.615128
31	1	0	5.344460	-0.317753	-0.236486
32	1	0	4.757984	-0.796542	1.351318
33	1	0	4.345243	-1.756195	-0.064790
34	1	0	2.547071	-1.544804	2.389886
35	1	0	1.932667	0.080357	2.720535
36	1	0	0.815372	-1.223977	2.323350
37	1	0	-3.782831	2.166732	-0.020853
38	1	0	-6.060693	1.166446	-0.063663
39	1	0	-6.321817	-1.297204	-0.027407
40	1	0	-4.319549	-2.751755	0.051225
41	1	0	-2.060121	-1.744193	0.093435

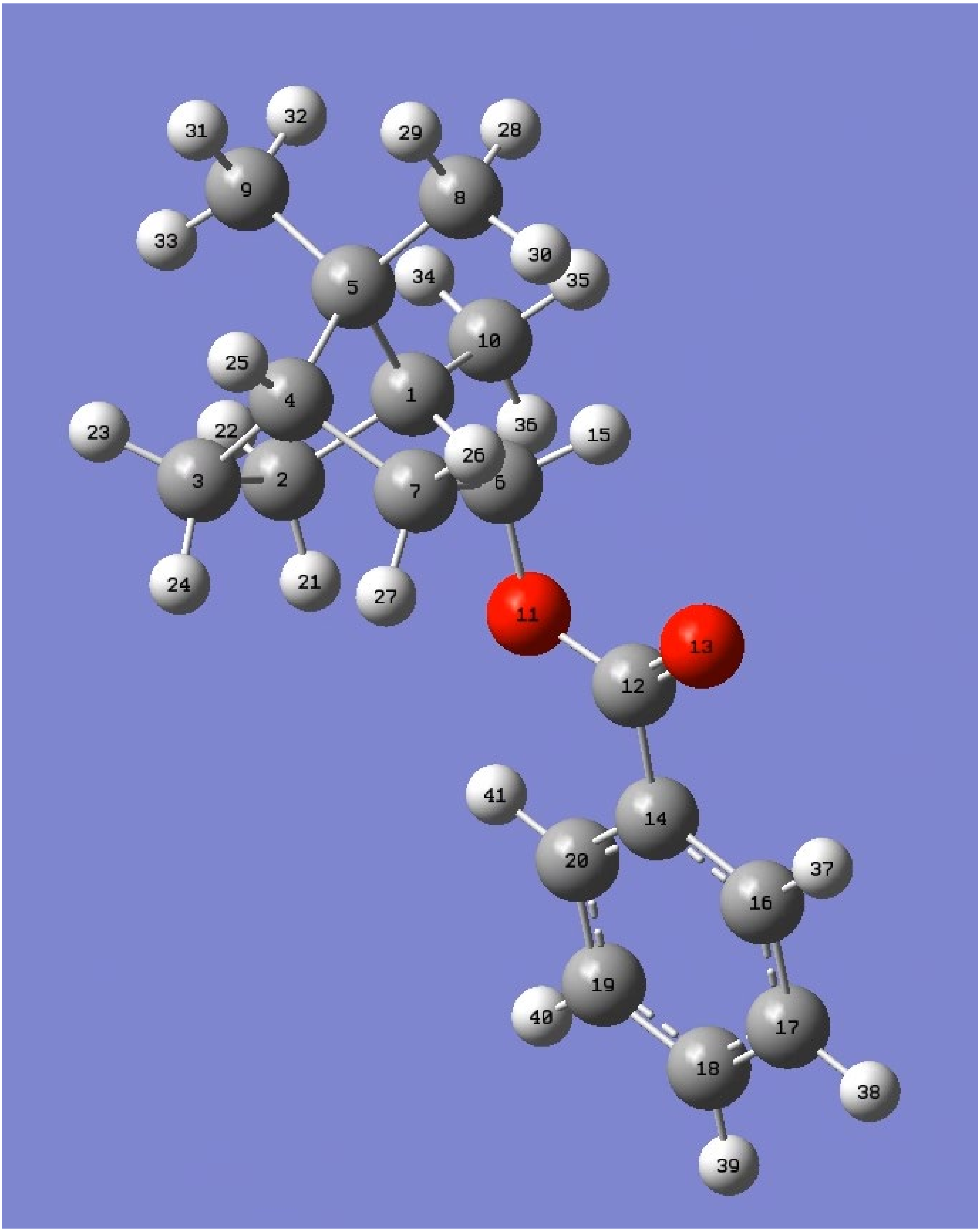
31
32
29
28
9
8
33
30
34
35
5
10
25
4
1
23
22
36
15
26
3
2
7
6
24
21
27
11
13
12
41
14
37
20
16
19
40
17
18
38
39

Compound bornyl benzoate **1c**

	In the gas phase	In chloroform	In benzene	In methanol
**Energy**	E(RB3LYP) -811.76185551 a.u.	E(RB3LYP) -811.76619934 a.u.	E(RB3LYP) -811.76455059 a.u.	E(RB3LYP) -811.76793468 a.u.
	E(RmPW1PW91) -811.57213985 a.u.	E(RmPW1PW91) -811.57659483 a.u.	E(RmPW1PW91) -811.57490015 a.u.	E(RmPW1PW91) -811.57838343 a.u.
	E(RwB97XD) -811.51392253 a.u.	E(RwB97XD) -811.51844405 a.u.	E(RwB97XD) -811.51672301 a.u.	E(RwB97XD) -811.52026156 a.u.

Reference: TMS B3LYP/6-311+G(2d,p) GIAO

Reference shielding: 31.8821 ppm

^{1}H NMR chemical shifts (ppm), GIAO//6-311+G(2d,p)

Atom	Atom	In the gas phase			In chloroform			In benzene			In methanol		
label	Assignment	B3LYP	mPW1PW91	wB97XD	B3LYP	mPW1PW91	wB97XD	B3LYP	mPW1PW91	wB97XD	B3LYP	mPW1PW91	wB97XD
15-H	2-exo	5.1443	5.1729	4.9157	5.1338	5.1625	4.9119	5.1392	5.1678	4.9146	5.1258	5.1548	4.9071
21-H	6-endo	2.2713	2.3224	2.2702	2.2460	2.2991	2.2444	2.2536	2.3057	2.2519	2.2407	2.2953	2.2403
22-H	6-exo	1.4353	1.4964	1.4103	1.4915	1.5544	1.4697	1.4680	1.5302	1.4449	1.5202	1.5837	1.4994
23-H	5-exo	1.8054	1.8583	1.7537	1.8359	1.8902	1.7859	1.8230	1.8768	1.7723	1.8513	1.9063	1.8024
24-H	5-endo	1.2359	1.2812	1.1885	1.2394	1.2865	1.1935	1.2392	1.2857	1.1929	1.2382	1.2858	1.1924
25-H	4-exo	1.5418	1.5854	1.5202	1.5973	1.6407	1.5758	1.5753	1.6189	1.5540	1.6220	1.6647	1.5996
26-H	3-exo	2.5929	2.6769	2.5701	2.5791	2.6613	2.5516	2.5891	2.6720	2.5636	2.5617	2.6429	2.5314
27-H	3-endo	0.9479	0.9879	0.9195	0.9551	0.9955	0.9227	0.9530	0.9934	0.9224	0.9564	0.9967	0.9219
28-H	9-CH3	0.8026	0.8715	0.8307	0.8298	0.9015	0.8612	0.8187	0.8892	0.8486	0.8428	0.9161	0.8763
29-H	9-CH3	0.7963	0.8642	0.8227	0.8252	0.8955	0.8523	0.8140	0.8834	0.8411	0.8375	0.9085	0.8640
30-H	9-CH3	1.1613	1.2710	1.1171	1.1294	1.2369	1.0845	1.1449	1.2531	1.1000	1.1077	1.2145	1.0630
31-H	8-CH3	0.7132	0.7801	0.7605	0.7373	0.8061	0.7871	0.7283	0.7965	0.7774	0.7461	0.8154	0.7963
32-H	8-CH3	0.7792	0.8453	0.8078	0.7972	0.8647	0.8279	0.7901	0.8569	0.8198	0.8053	0.8737	0.8371
33-H	8-CH3	1.1132	1.1966	1.1093	1.1277	1.2113	1.1240	1.1204	1.2038	1.1164	1.1384	1.2223	1.1354
34-H	10-CH3	0.6733	0.7371	0.7231	0.7219	0.7879	0.7741	0.7009	0.7661	0.7524	0.7483	0.8148	0.8005
35-H	10-CH3	0.7390	0.8021	0.7644	0.7697	0.8339	0.7989	0.7590	0.8229	0.7867	0.7792	0.8435	0.8100
36-H	10-CH3	1.1553	1.2283	1.2018	1.1145	1.1892	1.1618	1.1312	1.2051	1.1778	1.0951	1.1713	1.1442
37-H	13-o	8.5659	8.7493	8.6996	8.5276	8.7153	8.6673	8.5453	8.7311	8.6826	8.5049	8.6949	8.6473
38-H	14-m	7.6404	7.7942	7.7361	7.7683	7.9299	7.8725	7.7194	7.8780	7.8204	7.8194	7.9841	7.9268
39-H	15-p	7.7122	7.8792	7.8437	7.8850	8.0600	8.0248	7.8172	7.9891	7.9539	7.9585	8.1367	8.1015
40-H	14-m	7.5596	7.7204	7.6856	7.7147	7.8841	7.8508	7.6531	7.8191	7.7852	7.7827	7.9559	7.9231
41-H	13-o	8.4055	8.5714	8.5554	8.4954	8.6674	8.6502	8.4590	8.6284	8.6114	8.5371	8.7124	8.6953

Reference: TMS B3LYP/6-311+G(2d,p) GIAO

Reference shielding: 182.466 ppm

^{13}C NMR chemical shifts (ppm), GIAO//6-311+G(2d,p)

Atom	Atom	In the gas phase			In chloroform			In benzene			In methanol		
label	Assignment	B3LYP	mPW1PW91	wB97XD	B3LYP	mPW1PW91	wB97XD	B3LYP	mPW1PW91	wB97XD	B3LYP	mPW1PW91	wB97XD
1-C	1	54.3603	46.3221	45.8475	54.4880	46.4467	45.9691	54.4375	46.3977	45.9212	54.5448	46.5016	46.0227
2-C	6	30.5009	24.3739	23.8077	30.2982	24.1550	23.6010	30.3838	24.2473	23.6882	30.1968	24.0459	23.4979
3-C	5	30.6958	24.7194	24.0331	30.4875	24.4915	23.8106	30.5679	24.5792	23.8962	30.4031	24.3995	23.7207
4-C	4	50.1243	42.9485	41.6683	50.0491	42.8595	41.5833	50.0786	42.8943	41.6162	50.0182	42.8229	41.5494
5-C	7	54.7845	46.4794	45.6094	54.9211	46.6094	45.7241	54.8670	46.5581	45.6787	54.9823	46.6673	45.7748
6-C	2	84.8866	76.5650	75.7652	85.3346	76.9717	76.1299	85.1681	76.8203	75.9945	85.5071	77.1283	76.2689
7-C	3	40.4065	34.0222	33.8473	40.1789	33.7986	33.6128	40.2680	33.8854	33.7044	40.0779	33.7000	33.5083
8-C	9	19.4071	14.3494	14.1456	19.1283	14.0613	13.8625	19.2384	14.1744	13.9735	19.0080	13.9385	13.7429
9-C	8	20.1534	15.1628	14.9260	19.8531	14.8485	14.6097	19.9720	14.9727	14.7351	19.7234	14.7124	14.4723
10-C	10	14.0447	9.3275	9.1871	13.8209	9.0861	8.9427	13.9077	9.1796	9.0374	13.7281	8.9868	8.8414
12-C	11	171.7126	165.0167	164.8193	173.1030	166.3090	166.1800	172.5747	165.8177	165.6625	173.6623	166.8310	166.7303
14-C	13-ipso	136.3026	129.9085	130.4207	135.7698	129.3600	129.8528	135.9837	129.5807	130.0801	135.5275	129.1095	129.5964
16-C	14-o	135.8228	130.6631	130.7877	135.1293	129.9803	130.1169	135.4296	130.2755	130.4081	134.7629	129.6207	129.7605
17-C	15-m	132.0635	126.7926	126.9464	132.5169	127.2578	127.3980	132.3426	127.0789	127.2251	132.7002	127.4451	127.5769
18-C	16-p	136.6375	131.4309	131.6448	137.8434	132.6416	132.8393	137.3589	132.1551	132.3604	138.3875	133.1882	133.3749
19-C	15-m	131.4228	126.1306	126.3167	132.2347	126.9602	127.1358	131.8941	126.6126	126.7925	132.6378	127.3710	127.5413
20-C	14-o	133.7843	128.4338	128.7479	134.1037	128.7554	129.0492	133.9689	128.6186	128.9195	134.2666	128.9233	129.2119

Table S13. Optimized coordinates, energies, and calculated NMR chemical shifts of isobornyl acetate **2b**

Optimized coordinates in the gas phase

Calculation Method	RB3LYP
Basis Set	6-311+G(2d,p)
Charge	0
Spin	Singlet
E(RB3LYP)	-619.97224096 a.u.

Zero-point correction= 0.301278 (Hartree/Particle)
Thermal correction to Energy= 0.315864
Thermal correction to Enthalpy= 0.316808
Thermal correction to Gibbs Free Energy= 0.260081
Sum of electronic and zero-point Energies= -619.670963
Sum of electronic and thermal Energies= -619.656377
Sum of electronic and thermal Enthalpies= -619.655433
Sum of electronic and thermal Free Energies= -619.712160

Standard orientation:

Center Number	Atomic Number	Atomic Type	Coordinates (Angstroms) X	Y	Z
1	6	0	0.570225	-0.205992	0.706083
2	6	0	1.477741	-1.460567	0.856097
3	6	0	2.317388	-1.482648	-0.456057
4	6	0	1.788982	-0.246054	-1.214816
5	6	0	1.523816	0.777248	-0.071170
6	6	0	-0.452921	-0.637299	-0.383451
7	6	0	0.364536	-0.590865	-1.697164
8	6	0	0.873292	2.088710	-0.541357
9	6	0	2.788319	1.173513	0.709537
10	6	0	-0.043179	0.272361	2.010813
11	1	0	-0.853136	-1.623076	-0.161086
12	8	0	-1.595177	0.255161	-0.493764
13	6	0	-2.782718	-0.163073	-0.001612
14	8	0	-2.961389	-1.221151	0.548770
15	6	0	-3.852446	0.870284	-0.247946
16	1	0	0.882345	-2.365683	0.992303

17	1	0	2.112246	-1.367527	1.738608
18	1	0	3.385754	-1.405452	-0.250166
19	1	0	2.170513	-2.398531	-1.031751
20	1	0	2.447072	0.103851	-2.012180
21	1	0	-0.041067	0.175420	-2.357475
22	1	0	0.328293	-1.539089	-2.236537
23	1	0	1.571432	2.639522	-1.178735
24	1	0	-0.051740	1.951317	-1.094444
25	1	0	0.646909	2.729810	0.314908
26	1	0	2.532465	1.829091	1.545941
27	1	0	3.345450	0.329944	1.113365
28	1	0	3.466579	1.733499	0.059554
29	1	0	0.734768	0.528167	2.734362
30	1	0	-0.667324	1.156203	1.863954
31	1	0	-0.668612	-0.504567	2.457248
32	1	0	-4.779036	0.552974	0.223658
33	1	0	-3.540926	1.838603	0.146952
34	1	0	-4.007682	0.989734	-1.322261

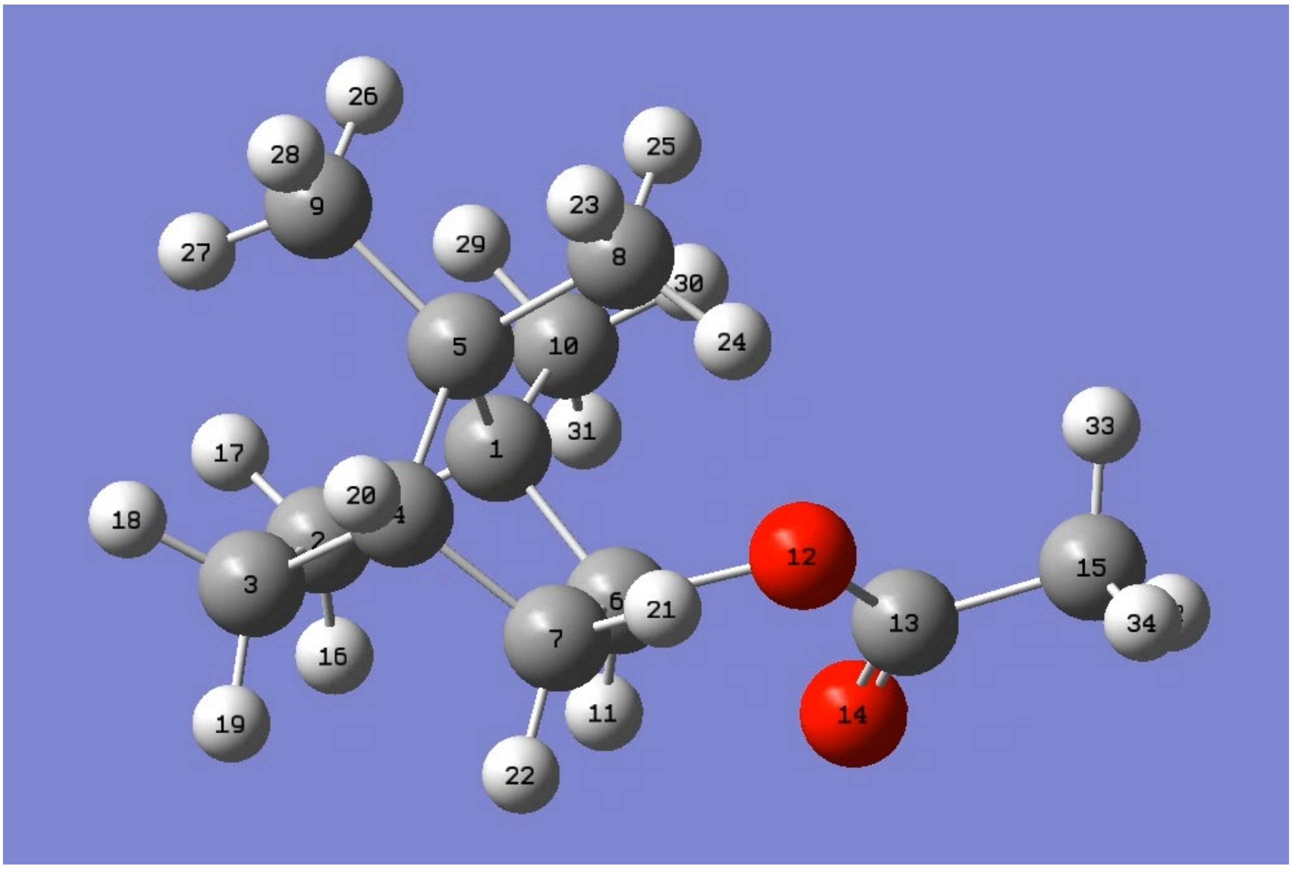

Compound isobornyl acetate **2b**

	In the gas phase	In chloroform	In benzene	In methanol
**Energy**	E(RB3LYP) -619.97224096 a.u.	E(RB3LYP) -619.97619065 a.u.	E(RB3LYP) -619.97473058 a.u.	E(RB3LYP) -619.97767299 a.u.
	E(RmPW1PW91) -619.82922334 a.u.	E(RmPW1PW91) -619.83314113 a.u.	E(RmPW1PW91) -619.83169213 a.u.	E(RmPW1PW91) -619.83461313 a.u.
	E(RwB97XD) -619.79542077 a.u.	E(RwB97XD) -619.79937765 a.u.	E(RwB97XD) -619.79790740 a.u.	E(RwB97XD) -619.80088086 a.u.

Reference: TMS B3LYP/6-311+G(2d,p) GIAO

Reference shielding: 31.8821 ppm

^{1}H NMR chemical shifts (ppm), GIAO//6-311+G(2d,p)

Atom	Atom	In the gas phase			In chloroform			In benzene			In methanol		
label	Assignment	B3LYP	mPW1PW91	wB97XD	B3LYP	mPW1PW91	wB97XD	B3LYP	mPW1PW91	wB97XD	B3LYP	mPW1PW91	wB97XD
11-H	2-endo	5.2267	5.2807	5.0514	5.1671	5.2189	4.9982	5.1915	5.2442	5.0202	5.1385	5.1893	4.9723
16-H	6-endo	1.1397	1.1925	1.1340	1.1248	1.1750	1.1166	1.1356	1.1869	1.1285	1.1057	1.1545	1.0961
17-H	6-exo	1.5099	1.5712	1.4612	1.5539	1.6156	1.5043	1.5361	1.5977	1.4871	1.5739	1.6358	1.5236
18-H	5-exo	1.6981	1.7621	1.6627	1.7179	1.7839	1.6847	1.7099	1.7751	1.6759	1.7269	1.7938	1.6943
19-H	5-endo	0.9975	1.0504	0.9802	1.0246	1.0790	1.0118	1.0145	1.0684	0.9998	1.0349	1.0899	1.0249
20-H	4-exo	1.5266	1.5770	1.4952	1.5780	1.6284	1.5470	1.5573	1.6078	1.5264	1.6014	1.6514	1.5700
21-H	3-exo	1.7809	1.8598	1.7873	1.7889	1.8698	1.7937	1.7849	1.8649	1.7906	1.7946	1.8767	1.7980
22-H	3-endo	1.5523	1.6019	1.5346	1.6138	1.6642	1.6025	1.5907	1.6409	1.5766	1.6376	1.6881	1.6298
23-H	9-CH3	0.6946	0.7567	0.7150	0.7278	0.7919	0.7503	0.7141	0.7776	0.7361	0.7433	0.8078	0.7660
24-H	9-CH3	1.4708	1.5590	1.5015	1.4102	1.5010	1.4421	1.4332	1.5228	1.4646	1.3863	1.4789	1.4188
25-H	9-CH3	0.7087	0.7668	0.7074	0.7242	0.7841	0.7254	0.7176	0.7766	0.7172	0.7323	0.7932	0.7359
26-H	8-CH3	0.7048	0.7614	0.7159	0.7096	0.7676	0.7202	0.7076	0.7650	0.7182	0.7120	0.7709	0.7230
27-H	8-CH3	0.8853	0.9694	0.8717	0.9122	0.9956	0.8992	0.9019	0.9855	0.8887	0.9230	1.0063	0.9099
28-H	8-CH3	0.7194	0.7796	0.7305	0.7352	0.7977	0.7499	0.7289	0.7906	0.7423	0.7422	0.8054	0.7581
29-H	10-CH3	0.4950	0.5449	0.4944	0.5628	0.6145	0.5636	0.5347	0.5858	0.5352	0.5957	0.6477	0.5967
30-H	10-CH3	0.7182	0.7486	0.6370	0.7479	0.7835	0.6838	0.7320	0.7655	0.6608	0.7721	0.8102	0.7161
31-H	10-CH3	0.8763	0.9612	1.0297	0.7687	0.8473	0.8949	0.8171	0.8979	0.9535	0.7069	0.7833	0.8223
32-H	12-CH3	1.6765	1.7604	1.7022	1.6787	1.7637	1.7070	1.6819	1.7666	1.7097	1.6707	1.7557	1.6987
33-H	12-CH3	1.9789	2.0204	1.9804	2.1205	2.1650	2.1320	2.0656	2.1088	2.0728	2.1796	2.2260	2.1966
34-H	12-CH3	2.0117	2.0583	1.9949	2.1510	2.2002	2.1344	2.0981	2.1464	2.0814	2.2059	2.2558	2.1897

Reference: TMS B3LYP/6-311+G(2d,p) GIAO

Reference shielding: 182.466 ppm

^{13}C NMR chemical shifts (ppm), GIAO//6-311+G(2d,p)

Atom label	Atom Assignment	In the gas phase			In chloroform			In benzene			In methanol		
		B3LYP	mPW1PW91	wB97XD	B3LYP	mPW1PW91	wB97XD	B3LYP	mPW1PW91	wB97XD	B3LYP	mPW1PW91	wB97XD
1-C	1	55.5593	47.4717	47.3053	55.6989	47.5953	47.4049	55.6462	47.5488	47.3672	55.7528	47.6427	47.4429
2-C	6	36.4894	30.2127	29.3246	36.3902	30.1023	29.2187	36.4169	30.1331	29.2471	36.3809	30.0890	29.2092
3-C	5	29.9511	23.9151	23.4060	29.7042	23.6502	23.1385	29.7990	23.7517	23.2414	29.6046	23.5440	23.0301
4-C	4	50.1861	42.9915	41.5338	50.1089	42.8984	41.4434	50.1396	42.9352	41.4789	50.0749	42.8584	41.4049
5-C	7	53.2611	45.1149	44.5077	53.3373	45.1903	44.5836	53.3049	45.1583	44.5514	53.3768	45.2290	44.6234
6-C	2	83.3964	74.9645	74.6232	83.9356	75.4446	75.0665	83.7264	75.2574	74.8920	84.1618	75.6481	75.2585
7-C	3	40.2497	34.0133	33.2699	40.0379	33.7851	33.0529	40.1154	33.8694	33.1331	39.9614	33.7005	32.9725
8-C	9	20.4678	15.4176	15.1969	20.1979	15.1435	14.9292	20.3044	15.2514	15.0347	20.0818	15.0261	14.8143
9-C	8	20.5440	15.5616	15.2890	20.2350	15.2390	14.9654	20.3577	15.3665	15.0934	20.0998	15.0993	14.8248
10-C	10	11.2030	6.4096	6.5502	11.1338	6.3372	6.4986	11.1505	6.3562	6.5110	11.1333	6.3324	6.4989
13-C	11	175.7237	169.0817	169.3051	178.2309	171.4664	171.7145	177.3014	170.5817	170.8174	179.1775	172.3686	172.6339
15-C	12	21.3079	16.5707	16.7475	21.8095	17.0164	17.1959	21.6082	16.8367	17.0146	22.0319	17.2160	17.3978

Table S14. Optimized coordinates, energies, and calculated NMR chemical shifts of isobornyl benzoate **2c**

Optimized coordinates in the gas phase

Calculation Method	RB3LYP	
Basis Set	6-311+G(2d,p)	
Charge	0	
Spin	Singlet	
E(RB3LYP)	-811.76071073	a.u.

Zero-point correction= 0.354769 (Hartree/Particle)
Thermal correction to Energy= 0.372117
Thermal correction to Enthalpy= 0.373062
Thermal correction to Gibbs Free Energy= 0.309299
Sum of electronic and zero-point Energies= -811.405942
Sum of electronic and thermal Energies= -811.388593
Sum of electronic and thermal Enthalpies= -811.387649
Sum of electronic and thermal Free Energies= -811.451412

Standard orientation:

Center Number	Atomic Number	Atomic Type	Coordinates (Angstroms) X	Y	Z
1	6	0	1.912559	-0.415990	0.614315
2	6	0	3.108088	-1.386244	0.390827
3	6	0	3.869594	-0.779675	-0.825457
4	6	0	3.019059	0.461944	-1.169987
5	6	0	2.558772	0.967687	0.229121
6	6	0	0.982295	-0.718346	-0.595667
7	6	0	1.704734	-0.053818	-1.792718
8	6	0	1.585016	2.156011	0.175811
9	6	0	3.717773	1.395488	1.145110
10	6	0	1.257523	-0.546377	1.978677
11	1	0	0.850494	-1.790217	-0.715454
12	8	0	-0.349841	-0.153020	-0.460577
13	6	0	-1.364591	-0.990309	-0.156429
14	8	0	-1.231606	-2.177450	0.034966
15	6	0	-2.678906	-0.285785	-0.086544
16	6	0	-3.808086	-1.042834	0.234361

17	6	0	-5.055249	-0.438731	0.312152
18	6	0	-5.184253	0.926526	0.070354
19	6	0	-4.062321	1.685438	-0.249506
20	6	0	-2.812457	1.083496	-0.328453
21	1	0	2.761281	-2.405141	0.206967
22	1	0	3.737187	-1.425814	1.281153
23	1	0	4.893474	-0.509303	-0.564048
24	1	0	3.929293	-1.470448	-1.668726
25	1	0	3.534616	1.199388	-1.787823
26	1	0	1.093783	0.755773	-2.191326
27	1	0	1.881241	-0.759132	-2.606727
28	1	0	1.227356	2.402674	1.178951
29	1	0	2.101841	3.040430	-0.208432
30	1	0	0.710758	1.981995	-0.445320
31	1	0	3.342554	1.671555	2.133945
32	1	0	4.479559	0.630716	1.286067
33	1	0	4.212329	2.278999	0.731697
34	1	0	0.870856	-1.556792	2.131565
35	1	0	1.977606	-0.342530	2.775019
36	1	0	0.422800	0.147636	2.095564
37	1	0	-3.686680	-2.102271	0.418492
38	1	0	-5.927495	-1.031025	0.561286
39	1	0	-6.157986	1.398327	0.131158
40	1	0	-4.161346	2.747718	-0.438087
41	1	0	-1.939248	1.670334	-0.576828

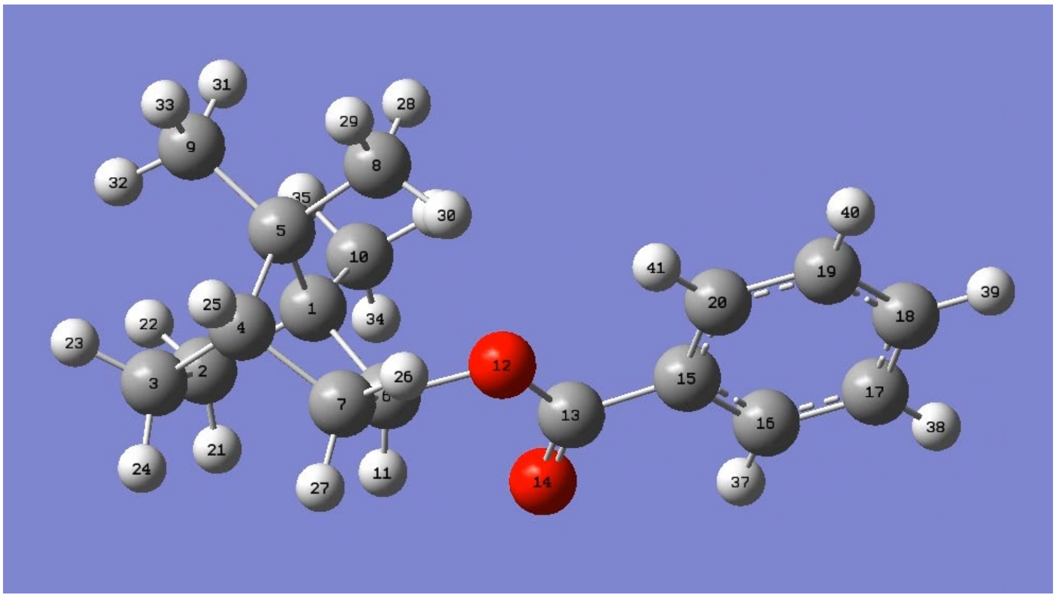

Compound isobornyl benzoate **2c**

	In the gas phase	In chloroform	In benzene	In methanol
**Energy**	E(RB3LYP) -811.76071073 a.u.	E(RB3LYP) -811.76486031 a.u.	E(RB3LYP) -811.76327665 a.u.	E(RB3LYP) -811.76653739 a.u.
	E(RmPW1PW91) -811.57101790 a.u.	E(RmPW1PW91) -811.57526232 a.u.	E(RmPW1PW91) -811.57363943 a.u.	E(RmPW1PW91) -811.57698347 a.u.
	E(RwB97XD) -811.51361318 a.u.	E(RwB97XD) -811.51791163 a.u.	E(RwB97XD) -811.51626965 a.u.	E(RwB97XD) -811.51965001 a.u.

Reference: TMS B3LYP/6-311+G(2d,p) GIAO

Reference shielding: 31.8821 ppm

^{1}H NMR chemical shifts (ppm), GIAO//6-311+G(2d,p)

Atom	Atom	In the gas phase			In chloroform			In benzene			In methanol		
label	Assignment	B3LYP	mPW1PW91	wB97XD	B3LYP	mPW1PW91	wB97XD	B3LYP	mPW1PW91	wB97XD	B3LYP	mPW1PW91	wB97XD
11-H	2-endo	5.5317	5.6042	5.3746	6.1967	5.5524	5.3294	5.5023	5.5738	5.3483	5.5317	5.5272	5.3068
21-H	6-endo	1.2651	1.3184	1.2475	1.9676	1.3043	1.2369	1.2620	1.3146	1.2457	1.2651	1.2860	1.2205
22-H	6-exo	1.5911	1.6581	1.5493	2.3538	1.7061	1.5966	1.6188	1.6863	1.5772	1.5911	1.7293	1.6191
23-H	5-exo	1.7445	1.8120	1.7355	2.4821	1.8360	1.7588	1.7574	1.8260	1.7492	1.7445	1.8477	1.7699
24-H	5-endo	1.0870	1.1429	1.0751	1.8316	1.1740	1.1084	1.1051	1.1622	1.0957	1.0870	1.1864	1.1220
25-H	4-exo	1.6285	1.6799	1.6179	2.3993	1.7357	1.6734	1.6608	1.7126	1.6505	1.6285	1.7631	1.7004
26-H	3-exo	2.0207	2.1078	2.0150	2.7562	2.1304	2.0329	2.0315	2.1201	2.0250	2.0207	2.1446	2.0436
27-H	3-endo	1.6593	1.7205	1.6551	2.4342	1.7814	1.7218	1.6968	1.7590	1.6970	1.6593	1.8038	1.7472
28-H	9-CH3	0.8584	0.9245	0.9004	1.5749	0.9266	0.9022	0.8601	0.9269	0.9022	0.8584	0.9240	0.9008
29-H	9-CH3	0.8272	0.8927	0.8677	1.5779	0.9306	0.9053	0.8469	0.9141	0.8890	0.8272	0.9513	0.9255
30-H	9-CH3	1.6934	1.7953	1.7631	2.3835	1.7717	1.7408	1.6759	1.7787	1.7476	1.6934	1.7677	1.7363
31-H	8-CH3	0.7599	0.8151	0.7669	1.4777	0.8187	0.7680	0.7617	0.8177	0.7680	0.7599	0.8192	0.7673
32-H	8-CH3	0.9389	1.0220	0.9417	1.6802	1.0472	0.9674	0.9548	1.0374	0.9575	0.9389	1.0577	0.9782
33-H	8-CH3	0.7604	0.8221	0.7783	1.4967	0.8455	0.8041	0.7722	0.8355	0.7932	0.7604	0.8576	0.8171
34-H	10-CH3	0.9504	1.0260	1.0476	1.5609	0.9174	0.9269	0.8926	0.9655	0.9794	0.9504	0.8565	0.8620
35-H	10-CH3	0.4975	0.5505	0.4920	1.2897	0.6300	0.5730	0.5416	0.5964	0.5390	0.4975	0.6709	0.6138
36-H	10-CH3	0.7513	0.7925	0.6715	1.4696	0.7962	0.6772	0.7521	0.7943	0.6742	0.7513	0.7988	0.6810
37-H	13-o	8.6036	8.7822	8.7021	9.2802	8.7481	8.6711	8.5827	8.7641	8.6860	8.6036	8.7274	8.6517
38-H	14-m	7.6123	7.7735	7.7297	8.4544	7.9078	7.8651	7.6904	7.8563	7.8132	7.6123	7.9620	7.9199
39-H	15-p	7.7058	7.8730	7.8342	8.5916	8.0511	8.0123	7.8090	7.9811	7.9422	7.7058	8.1275	8.0888
40-H	14-m	7.5529	7.7076	7.6649	8.4207	7.8686	7.8271	7.6443	7.8043	7.7623	7.5529	7.9405	7.8996
41-H	13-o	8.3114	8.4756	8.4791	9.1132	8.5671	8.5680	8.3623	8.5294	8.5314	8.3114	8.6115	8.6107

Reference: TMS B3LYP/6-311+G(2d,p) GIAO

Reference shielding: 182.466 ppm

^{13}C NMR chemical shifts (ppm), GIAO//6-311+G(2d,p)

Atom	Atom	In the gas phase			In chloroform			In benzene			In methanol		
label	Assignment	B3LYP	mPW1PW91	wB97XD	B3LYP	mPW1PW91	wB97XD	B3LYP	mPW1PW91	wB97XD	B3LYP	mPW1PW91	wB97XD
1-C	1	56.1196	48.0531	47.7044	56.2747	48.1960	47.8232	56.2170	48.1430	47.8232	56.3329	48.2491	47.8679
2-C	6	36.6968	30.4235	29.1695	36.6027	30.3140	29.0425	36.6281	30.3456	29.0425	36.5945	30.2984	29.0178
3-C	5	29.9167	23.8657	23.5367	29.6719	23.6021	23.2721	29.7661	23.7031	23.2721	29.5733	23.4966	23.1656
4-C	4	50.2464	43.0610	41.7031	50.1743	42.9741	41.6180	50.2038	43.0092	41.6180	50.1411	42.9349	41.5803
5-C	7	53.2826	45.2176	44.3918	53.3767	45.3110	44.4790	53.3372	45.2719	44.4790	53.4252	45.3583	44.5227
6-C	2	83.7279	75.3551	74.5035	84.3515	75.9332	75.0494	84.1080	75.7071	75.0494	84.6204	76.1831	75.2851
7-C	3	40.2450	34.0417	33.2097	40.0107	33.7896	32.9653	40.0995	33.8857	32.9653	39.9179	33.6881	32.8677
8-C	9	20.8146	15.8058	15.7618	20.5446	15.5293	15.5141	20.6496	15.6365	15.5141	20.4316	15.4149	15.4165
9-C	8	20.4959	15.5251	15.2222	20.1996	15.2149	14.9125	20.3172	15.3372	14.9125	20.0699	15.0811	14.7778
10-C	10	11.4197	6.6038	6.5718	11.3674	6.5470	6.5258	11.3762	6.5589	6.5258	11.3789	6.5529	6.5304
13-C	11	171.0660	164.3462	164.3543	172.3422	165.5263	165.5906	171.8546	165.0759	165.5906	172.8609	166.0044	166.0902
15-C	12-ipso	135.9412	129.5834	130.3434	135.4598	129.0858	129.8185	135.6559	129.2890	129.8185	135.2319	128.8496	129.5700
16-C	13-o	136.3816	131.1656	131.1478	135.7046	130.4980	130.4955	136.0012	130.7905	130.4955	135.3369	130.1350	130.1380
17-C	14-m	132.0114	126.7463	126.9431	132.4535	127.2020	127.3962	132.2814	127.0248	127.3962	132.6382	127.3921	127.5843
18-C	15-p	136.5821	131.3963	131.7201	137.7468	132.5662	132.8775	137.2753	132.0929	132.8775	138.2824	133.1033	133.4085
19-C	14-m	131.4079	126.1124	126.2587	132.2041	126.9259	127.0652	131.8658	126.5805	127.0652	132.6122	127.3420	127.4787
20-C	13-o	133.4890	128.1776	128.7181	133.7787	128.4681	128.9998	133.6556	128.3435	128.9998	133.9289	128.6214	129.1531

Table S15. Optimized coordinates, energies, and calculated NMR chemical shifts of bornyl TBDMS **1d**

Optimized coordinates in the gas phase

Calculation Method	RB3LYP
Basis Set	6-311+G(2d,p)
Charge	0
Spin	Singlet
E(RB3LYP)	-994.00809602 a.u.

Zero-point correction= 0.448989 (Hartree/Particle)
Thermal correction to Energy= 0.471953
Thermal correction to Enthalpy= 0.472898
Thermal correction to Gibbs Free Energy= 0.397821
Sum of electronic and zero-point Energies= -993.559107
Sum of electronic and thermal Energies= -993.536143
Sum of electronic and thermal Enthalpies= -993.535198
Sum of electronic and thermal Free Energies= -993.610275

Standard orientation:

Center Number	Atomic Number	Atomic Type	Coordinates (Angstroms) X	Y	Z
1	6	0	1.857380	-0.199674	0.719244
2	6	0	1.803929	-1.707946	0.362915
3	6	0	2.435123	-1.785064	-1.058231
4	6	0	2.739047	-0.306087	-1.375765
5	6	0	3.185254	0.257391	0.007706
6	6	0	0.789826	0.502780	-0.165477
7	6	0	1.381727	0.392321	-1.602293
8	6	0	3.441288	1.774521	0.009166
9	6	0	4.454947	-0.400555	0.569899
10	6	0	1.707219	0.094082	2.202323
11	8	0	-0.493743	-0.100361	-0.031382
12	1	0	0.716964	1.551336	0.139435
13	14	0	-1.953467	0.701389	-0.098692
14	6	0	-2.048421	1.989105	1.273543
15	6	0	-2.142122	1.586420	-1.751273
16	6	0	-3.261369	-0.669273	0.123701

17	6	0	-3.059540	-1.369623	1.481769
18	6	0	-4.677699	-0.063054	0.074009
19	6	0	-3.119150	-1.712743	-1.001752
20	1	0	0.777808	-2.071072	0.385652
21	1	0	2.370188	-2.293306	1.089696
22	1	0	1.754712	-2.218961	-1.793280
23	1	0	3.341582	-2.392773	-1.065193
24	1	0	3.453587	-0.162671	-2.189037
25	1	0	1.492713	1.373815	-2.066939
26	1	0	0.720641	-0.197190	-2.238757
27	1	0	4.340457	1.999855	-0.571301
28	1	0	2.629928	2.365596	-0.412855
29	1	0	3.617376	2.135590	1.026038
30	1	0	4.383370	-1.482270	0.668338
31	1	0	5.307696	-0.184580	-0.080383
32	1	0	4.692718	0.004147	1.557330
33	1	0	2.480898	-0.407234	2.789582
34	1	0	1.767900	1.166066	2.411221
35	1	0	0.738764	-0.258105	2.566193
36	1	0	-3.008695	2.511575	1.256409
37	1	0	-1.926267	1.540459	2.261786
38	1	0	-1.268846	2.746809	1.156022
39	1	0	-2.094589	0.893621	-2.594275
40	1	0	-1.347695	2.325842	-1.886719
41	1	0	-3.094163	2.121072	-1.807463
42	1	0	-3.795770	-2.172753	1.605864
43	1	0	-2.065424	-1.814487	1.561190
44	1	0	-3.187270	-0.680463	2.320838
45	1	0	-5.429816	-0.850485	0.201177
46	1	0	-4.882216	0.427679	-0.881486
47	1	0	-4.840049	0.669813	0.869133
48	1	0	-2.126555	-2.167971	-1.004640
49	1	0	-3.854730	-2.514992	-0.868501
50	1	0	-3.291375	-1.276139	-1.989227

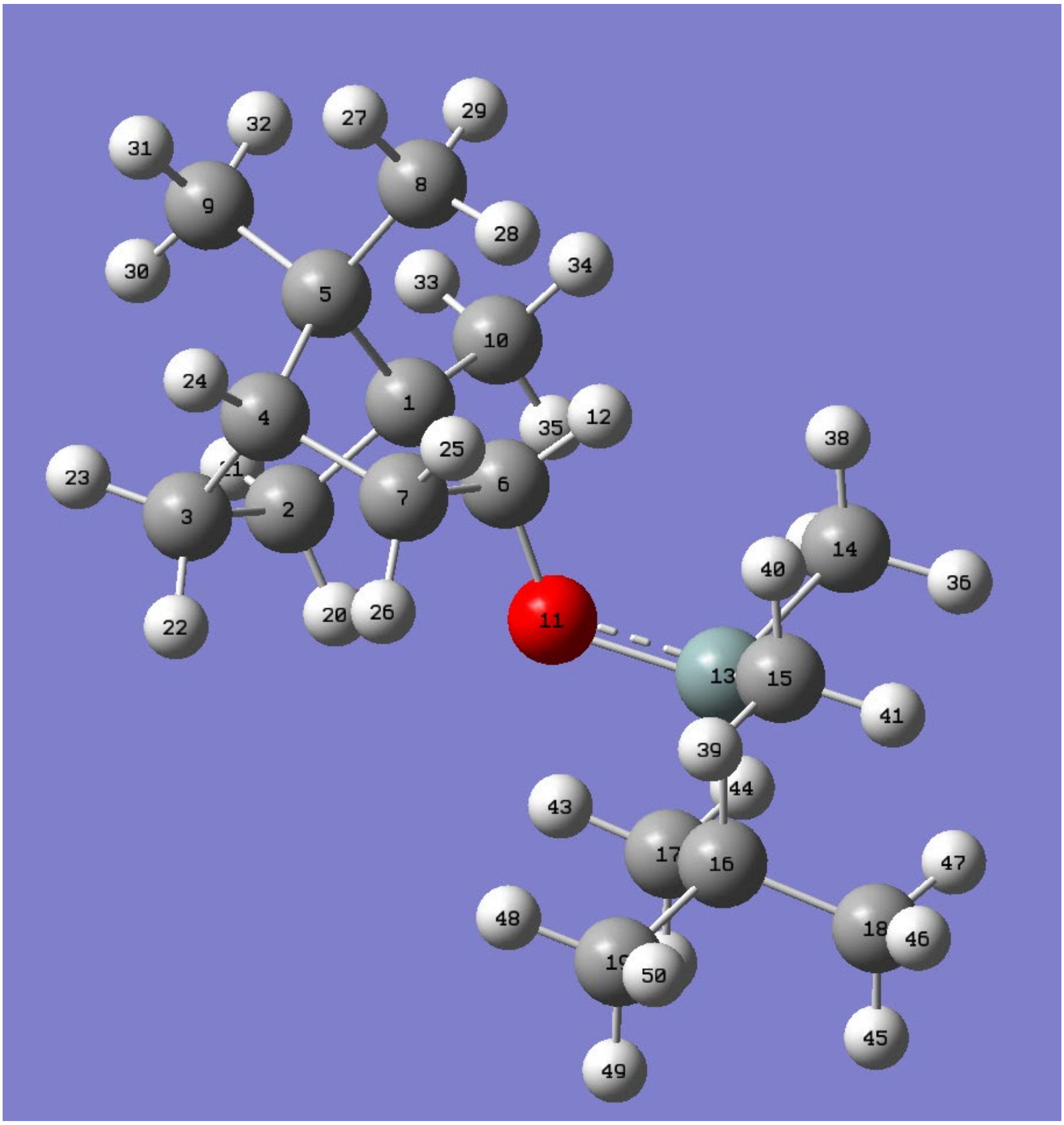

1
2
3
4
5
6
7
8
9
10
11
12
13
14
15
16
17
18
20
22
23
24
25
26
27
28
29
30
31
32
33
34
35
36
38
39
40
41
43
44
45
46
47
48
49
50

Compound bornyl TBDMS **1d**

	In the gas phase	In chloroform	In benzene
**Energy**	E(RB3LYP) -994.00809602 a.u.	E(RB3LYP) -994.00935579 a.u.	E(RB3LYP) -994.00882853 a.u.
	E(RmPW1PW91) -993.83663257 a.u.	E(RmPW1PW91) -993.83703982 a.u.	E(RmPW1PW91) -993.83645671 a.u.
	E(RwB97XD) -993.79573697 a.u.	E(RwB97XD) -993.79580668 a.u.	E(RwB97XD) -993.79521911 a.u.

Reference: TMS B3LYP/6-311+G(2d,p) GIAO

Reference shielding: 31.8821 ppm

^{1}H NMR chemical shifts (ppm), GIAO//6-311+G(2d,p)

Atom	Atom	In the gas phase			In chloroform			In benzene		
label	Assignment	B3LYP	mPW1PW91	wB97XD	B3LYP	mPW1PW91	wB97XD	B3LYP	mPW1PW91	wB97XD
12-H	2-exo	3.9975	4.0443	3.8549	4.0590	4.1717	4.0050	4.0308	4.1440	3.9773
20-H	6-endo	2.1834	2.2489	2.1735	2.1072	2.1894	2.0866	2.1413	2.2238	2.1233
21-H	6-exo	1.1363	1.1882	1.0886	1.1560	1.2478	1.1659	1.1473	1.2380	1.1554
22-H	5-endo	1.1890	1.2384	1.1576	1.1723	1.2708	1.1928	1.1815	1.2791	1.2013
23-H	5-exo	1.6745	1.7307	1.6676	1.6865	1.7695	1.6821	1.6804	1.7625	1.6744
24-H	4-exo	1.4381	1.4797	1.4178	1.4607	1.5840	1.4790	1.4515	1.5745	1.4686
25-H	3-exo	2.1503	2.1978	2.1012	2.1995	2.2671	2.1742	2.1769	2.2429	2.1488
26-H	3-endo	0.8659	0.9153	0.8729	0.8355	0.9463	0.8475	0.8521	0.9632	0.8655
27-H	9-CH3	0.6636	0.7277	0.7278	0.6707	0.7783	0.7501	0.6687	0.7750	0.7463
28-H	9-CH3	0.9854	1.0717	0.9364	1.0121	1.0897	0.9553	0.9992	1.0768	0.9425
29-H	9-CH3	0.7022	0.7754	0.7482	0.7094	0.8118	0.7586	0.7062	0.8074	0.7545
30-H	8-CH3	1.0786	1.1664	1.0862	1.0743	1.1553	1.0697	1.0765	1.1573	1.0721
31-H	8-CH3	0.6002	0.6580	0.6621	0.6139	0.7201	0.6942	0.6085	0.7134	0.6870
32-H	8-CH3	0.6903	0.7533	0.7121	0.6972	0.7961	0.7414	0.6941	0.7920	0.7372
33-H	10-CH3	0.5509	0.5987	0.6028	0.5645	0.6644	0.6116	0.5583	0.6565	0.6031
34-H	10-CH3	0.5508	0.6013	0.5430	0.5885	0.6906	0.6188	0.5702	0.6715	0.5995
35-H	10-CH3	1.0424	1.1006	1.0097	0.9901	1.0919	1.0127	1.0149	1.1174	1.0393
36-H	11-CH3	-0.2486	-0.1808	-0.2530	-0.2196	-0.1005	-0.1593	-0.2331	-0.1161	-0.1759
37-H	11-CH3	-0.1501	-0.0590	-0.0516	-0.1657	-0.0329	-0.1175	-0.1564	-0.0250	-0.1086
38-H	11-CH3	0.0770	0.1808	0.0603	0.1164	0.2509	0.1649	0.0979	0.2313	0.1449
39-H	11-CH3	0.0352	0.1205	0.0229	0.0238	0.1506	0.0683	0.0313	0.1560	0.0745
40-H	11-CH3	-0.0993	0.0046	-0.0153	-0.0615	0.0823	0.0023	-0.0798	0.0635	-0.0171
41-H	11-CH3	-0.1700	-0.1022	-0.1871	-0.1456	-0.0333	-0.1090	-0.1565	-0.0460	-0.1226
42-H	13-CH3-17	0.5053	0.5840	0.5705	0.5008	0.6223	0.5768	0.5011	0.6200	0.5736
43-H	13-CH3-17	1.3130	1.4015	1.3851	1.2606	1.3587	1.2682	1.2861	1.3848	1.2962
44-H	13-CH3-17	0.9221	0.9964	0.9342	0.9582	1.0632	0.9759	0.9422	1.0469	0.9593
45-H	13-CH3-18	0.5671	0.6508	0.6509	0.5480	0.6680	0.6273	0.5561	0.6738	0.6330
46-H	13-CH3-18	0.8135	0.8811	0.8144	0.8419	0.9401	0.8502	0.8295	0.9277	0.8376
47-H	13-CH3-18	0.8192	0.8855	0.8104	0.8464	0.9425	0.8532	0.8341	0.9301	0.8407
48-H	13-CH3-19	1.3471	1.4341	1.3639	1.2871	1.3860	1.2953	1.3157	1.4154	1.3267
49-H	13-CH3-19	0.4976	0.5768	0.5595	0.4950	0.6172	0.5698	0.4944	0.6140	0.5657
50-H	13-CH3-19	0.9345	1.0070	0.9264	0.9736	1.0796	0.9879	0.9562	1.0620	0.9699

Reference: TMS B3LYP/6-311+G(2d,p) GIAO

Reference shielding: 182.466 ppm

^{13}C NMR chemical shifts (ppm), GIAO//6-311+G(2d,p)

Atom	Atom	In the gas phase			In chloroform			In benzene		
label	Assignment	B3LYP	mPW1PW91	wB97XD	B3LYP	mPW1PW91	wB97XD	B3LYP	mPW1PW91	wB97XD
1-C	1	55.5518	47.5203	47.0455	55.5426	50.0751	49.2692	55.5473	50.0791	49.2731
2-C	6	28.9862	22.7922	22.1237	28.8576	23.4163	22.3869	28.9090	23.4748	22.4387
3-C	5	30.9005	24.9331	24.1289	30.7232	25.4375	24.5924	30.7869	25.5081	24.6577
4-C	4	50.6401	43.4737	42.0639	50.5290	44.7102	42.8573	50.5750	44.7628	42.9079
5-C	7	54.3263	45.9836	45.3218	54.3663	48.5919	47.2642	54.3500	48.5720	47.2473
6-C	2	81.5199	73.4521	72.1028	81.3562	75.2897	74.0298	81.4328	75.3564	74.0961
7-C	3	42.7481	36.2559	35.5438	42.6682	36.7051	36.1003	42.6870	36.7255	36.1097
8-C	9-CH3	19.2207	14.1679	13.9569	18.9474	14.2436	13.7297	19.0536	14.3537	13.8389
9-C	8-CH3	20.5568	15.5446	15.4407	20.2832	15.5656	15.0353	20.3908	15.6752	15.1435
10-C	10-CH3	13.7944	8.9853	9.4552	13.5749	9.1057	8.6708	13.6598	9.1950	8.7552
14-C	11-DMS	-7.4228	-11.5917	-11.2810	-7.7201	-11.3158	-11.9177	-7.6027	-11.1947	-11.7960
15-C	11-DMS	-4.8392	-9.1701	-10.6859	-5.1914	-8.9296	-9.6780	-5.0485	-8.7874	-9.5353
16-C	12-TB	24.4301	16.2708	14.7386	24.3460	18.6314	17.1186	24.3809	18.6660	17.1511
17-C	13-TB	25.8511	20.7182	20.3079	25.6457	20.7591	20.2054	25.7236	20.8453	20.2863
18-C	13-TB	25.9508	20.8028	20.0906	25.6011	20.6833	20.0228	25.7434	20.8299	20.1713
19-C	13-TB	25.7144	20.5980	20.0910	25.5116	20.6327	20.0820	25.5885	20.7179	20.1610

Table S16. Optimized coordinates, energies, and calculated NMR chemical shifts of bornyl DMMPS **1e**

Optimized coordinates in the gas phase

Calculation Method	RB3LYP
Basis Set	6-311+G(2d,p)
Charge	0
Spin	Singlet
E(RB3LYP)	-1067.82585748 a.u.

Zero-point correction= 0.418777 (Hartree/Particle)
Thermal correction to Energy= 0.440619
Thermal correction to Enthalpy= 0.441563
Thermal correction to Gibbs Free Energy= 0.367469
Sum of electronic and zero-point Energies= -1067.407080
Sum of electronic and thermal Energies= -1067.385239
Sum of electronic and thermal Enthalpies= -1067.384295
Sum of electronic and thermal Free Energies= -1067.458389

Standard orientation:

Center Number	Atomic Number	Atomic Type	Coordinates (Angstroms) X	Y	Z
1	6	0	-1.963369	-0.440191	0.672930
2	6	0	-2.752667	0.752365	1.272481
3	6	0	-3.561217	1.316907	0.067774
4	6	0	-3.109677	0.412445	-1.097927
5	6	0	-2.964065	-0.981370	-0.415372
6	6	0	-0.851291	0.168710	-0.225386
7	6	0	-1.646558	0.787298	-1.414480
8	6	0	-2.398380	-2.072546	-1.340603
9	6	0	-4.276970	-1.533288	0.161373
10	6	0	-1.442190	-1.421460	1.709207
11	8	0	-0.055366	1.110439	0.491486
12	1	0	-0.195667	-0.633890	-0.574377
13	14	0	1.510555	1.539213	0.130639
14	6	0	1.605620	2.513591	-1.474232
15	6	0	2.046416	2.604693	1.572838
16	6	0	2.583545	-0.009212	0.001941

17	6	0	2.639053	-0.916873	1.070350
18	6	0	3.433104	-2.056635	1.014824
19	6	0	4.196761	-2.317870	-0.119450
20	6	0	4.158795	-1.434495	-1.192113
21	6	0	3.360068	-0.295535	-1.128186
22	1	0	-2.071419	1.485593	1.701320
23	1	0	-3.408665	0.411937	2.075879
24	1	0	-4.637411	1.249469	0.236080
25	1	0	-3.334984	2.366225	-0.130928
26	1	0	-3.772445	0.439094	-1.965476
27	1	0	-1.312779	0.382770	-2.371945
28	1	0	-1.503769	1.868099	-1.448438
29	1	0	-3.134674	-2.326744	-2.108466
30	1	0	-1.480344	-1.791907	-1.854369
31	1	0	-2.192836	-2.986987	-0.777740
32	1	0	-4.104828	-2.489706	0.662313
33	1	0	-4.755570	-0.869443	0.879202
34	1	0	-4.992248	-1.716865	-0.645730
35	1	0	-2.257243	-1.847996	2.299720
36	1	0	-0.891597	-2.245422	1.247074
37	1	0	-0.763059	-0.919172	2.402651
38	1	0	2.626834	2.850934	-1.672203
39	1	0	1.271701	1.931516	-2.336502
40	1	0	0.972344	3.402085	-1.408999
41	1	0	3.067780	2.967658	1.431859
42	1	0	2.015606	2.046176	2.511045
43	1	0	1.389170	3.471667	1.677640
44	1	0	2.048614	-0.735471	1.962459
45	1	0	3.457193	-2.741641	1.854689
46	1	0	4.817134	-3.205422	-0.165825
47	1	0	4.749779	-1.631788	-2.079258
48	1	0	3.346672	0.377527	-1.978316

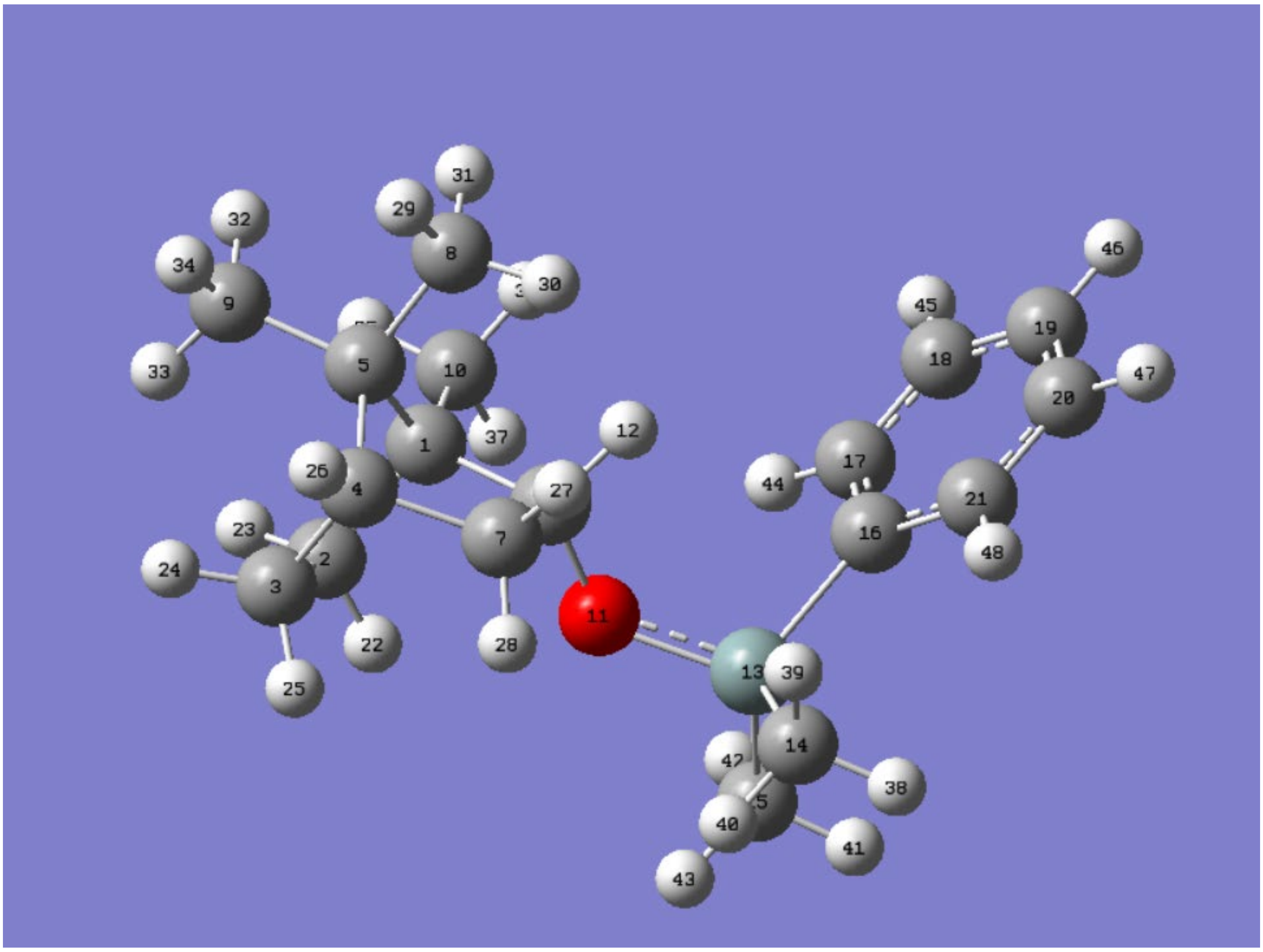
31
29
32
46
34
8
30
45
19
9
5
10
18
47
33
20
37
12
1
17
26
44
4
27
21
23
7
16
48
24
2
3
11
22
28
13
39
25
14
42
38
40
41
43

Compound bornyl DMMPS **1e**

	In the gas phase	In chloroform	In benzene
**Energy**	E(RB3LYP) -1067.82585748 a.u.	E(RB3LYP) -1067.82855572 a.u.	E(RB3LYP) -1067.82747019 a.u.
	E(RmPW1PW91) -1067.63486295 a.u.	E(RmPW1PW91) -1067.63696944 a.u.	E(RmPW1PW91) -1067.63574844 a.u.
	E(RwB97XD) -1067.57397471 a.u.	E(RwB97XD) -1067.57536350 a.u.	E(RwB97XD) -1067.57412829 a.u.

Reference: TMS B3LYP/6-311+G(2d,p) GIAO

Reference shielding: 31.8821 ppm

^{1}H NMR chemical shifts (ppm), GIAO//6-311+G(2d,p)

Atom	Atom	In the gas phase			In chloroform			In benzene		
label	Assignment	B3LYP	mPW1PW91	wB97XD	B3LYP	mPW1PW91	wB97XD	B3LYP	mPW1PW91	wB97XD
12-H	2-exo	3.7504	3.7914	3.6218	3.7547	3.8677	3.6935	3.7545	3.8701	3.6965
22-H	6-endo	2.2560	2.3193	2.2389	2.1465	2.2309	2.1281	2.1943	2.2791	2.1779
23-H	6-exo	1.1333	1.1826	1.1247	1.1675	1.2642	1.1733	1.1518	1.2472	1.1561
24-H	5-exo	1.6914	1.7488	1.6538	1.7066	1.7912	1.6939	1.6989	1.7824	1.6846
25-H	5-endo	1.1896	1.2234	1.1463	1.1819	1.2789	1.1877	1.1862	1.2821	1.1908
26-H	4-exo	1.3989	1.4354	1.3640	1.4278	1.5528	1.4632	1.4149	1.5395	1.4490
27-H	3-exo	2.0392	2.0781	1.9711	2.0939	2.1689	2.0719	2.0695	2.1430	2.0454
28-H	3-endo	0.8981	0.9394	0.8307	0.8988	1.0094	0.9022	0.9001	1.0097	0.9032
29-H	9-CH3	0.4975	0.5441	0.5077	0.5125	0.6142	0.5657	0.5051	0.6052	0.5560
30-H	9-CH3	0.6336	0.7047	0.5405	0.6389	0.7156	0.5980	0.6378	0.7154	0.5981
31-H	9-CH3	0.4741	0.5142	0.4342	0.4766	0.5748	0.5072	0.4764	0.5736	0.5061
32-H	8-CH3	0.6249	0.6810	0.6324	0.6331	0.7323	0.6729	0.6296	0.7277	0.6684
33-H	8-CH3	1.0629	1.1424	1.0412	1.0596	1.1388	1.0328	1.0607	1.1394	1.0337
34-H	8-CH3	0.5428	0.6029	0.5891	0.5609	0.6704	0.6374	0.5529	0.6611	0.6276
35-H	10-CH3	0.5401	0.5861	0.6151	0.5660	0.6639	0.6048	0.5526	0.6485	0.5884
36-H	10-CH3	0.4343	0.4832	0.4630	0.4448	0.5464	0.4705	0.4419	0.5441	0.4684
37-H	10-CH3	1.1484	1.2067	1.2350	1.1210	1.2244	1.1676	1.1337	1.2362	1.1801
38-H	11-CH3-14	0.2847	0.3655	0.3740	0.3341	0.4543	0.3744	0.3135	0.4311	0.3500
39-H	11-CH3-14	0.4566	0.5665	0.4057	0.4932	0.6327	0.5403	0.4752	0.6130	0.5208
40-H	11-CH3-14	0.2264	0.3354	0.2680	0.2451	0.3978	0.3250	0.2404	0.3901	0.3175
41-H	11-CH3-15	-0.2254	-0.1366	-0.1847	-0.1820	-0.0440	-0.1446	-0.2006	-0.0636	-0.1651
42-H	11-CH3-15	0.3705	0.4686	0.4025	0.3610	0.5028	0.4267	0.3671	0.5062	0.4311
43-H	11-CH3-15	0.3059	0.4057	0.3475	0.3064	0.4534	0.3733	0.3079	0.4515	0.3717
44-H	13-o-17	7.8683	8.0444	8.0932	7.9349	8.1594	8.1177	7.9099	8.1325	8.0915
45-H	14-m-18	7.5822	7.7386	7.6859	7.6793	7.8895	7.8396	7.6416	7.8472	7.7968
46-H	15-p	7.6004	7.7613	7.7025	7.7041	7.9204	7.8579	7.6631	7.8746	7.8109
47-H	14-m-20	7.5340	7.6961	7.6409	7.6380	7.8505	7.7924	7.5960	7.8037	7.7445
48-H	13-o-21	7.6719	7.8535	7.8043	7.7650	7.9859	7.9532	7.7255	7.9438	7.9107

Reference: TMS B3LYP/6-311+G(2d,p) GIAO

Reference shielding: 182.466 ppm

^{13}C NMR chemical shifts (ppm), GIAO//6-311+G(2d,p)

Atom	Atom	In the gas phase			In chloroform			In benzene		
label	Assignment	B3LYP	mPW1PW91	wB97XD	B3LYP	mPW1PW91	wB97XD	B3LYP	mPW1PW91	wB97XD
1-C	1	55.9709	47.9731	47.1502	55.9847	50.4810	49.3907	55.9794	50.4733	49.3830
2-C	6	29.0169	22.8306	22.3577	28.9220	23.4889	22.7153	28.9593	23.5345	22.7535
3-C	5	31.0565	25.0863	24.4706	30.8367	25.5376	24.7464	30.9210	25.6280	24.8322
4-C	4	50.5799	43.4788	42.3436	50.4530	44.6417	43.1004	50.5056	44.6993	43.1569
5-C	7	53.9050	45.6726	44.8396	53.9455	48.2095	46.9087	53.9285	48.1884	46.8907
6-C	2	81.8118	73.8050	72.3463	81.9185	75.9259	74.6466	81.8621	75.8590	74.5742
7-C	3	42.5450	36.1310	35.8206	42.4177	36.5230	35.7859	42.4578	36.5638	35.8192
8-C	9	18.9885	13.9675	13.6619	18.6901	13.9929	13.4573	18.8116	14.1182	13.5826
9-C	8	20.5559	15.5776	15.3570	20.2739	15.5396	15.0507	20.3861	15.6538	15.1642
10-C	10	14.1297	9.3620	9.9339	13.9395	9.4140	8.8525	14.0035	9.4828	8.9146
14-C	11-DM	-2.1839	-6.4280	-7.0178	-2.4816	-6.4151	-7.0803	-2.3707	-6.3112	-6.9721
15-C	11-DM	-0.6381	-5.0682	-5.7147	-1.0531	-4.9242	-5.7177	-0.8888	-4.7622	-5.5571
16-C	12-ipso	143.2982	136.4149	136.6192	143.6397	137.9342	137.8593	143.5248	137.8345	137.7665
17-C	13-o	138.8767	133.5659	134.2045	138.9099	134.4493	134.5402	138.9203	134.4662	134.5656
18-C	14-m	131.5457	126.3267	126.7070	131.5962	127.1717	127.5744	131.5823	127.1323	127.5274
19-C	15-p	133.5354	128.4990	128.7321	133.7149	129.4555	129.7577	133.6357	129.3479	129.6395
20-C	14-m	131.2886	126.1014	126.5079	131.4192	127.0531	127.4508	131.3507	126.9590	127.3453
21-C	13-o	138.7462	133.6079	133.4421	139.0431	134.6543	134.7384	138.8919	134.5064	134.5845

Table S17. Optimized coordinates, energies, and calculated NMR chemical shifts of bornyl TBDPS **1f**

Optimized coordinates in the gas phase

Calculation Method	RB3LYP
Basis Set	6-311+G(2d,p)
Charge	0
Spin	Singlet
E(RB3LYP)	-1377.56838797 a.u.

Zero-point correction= 0.557788 (Hartree/Particle)
Thermal correction to Energy= 0.586737
Thermal correction to Enthalpy= 0.587682
Thermal correction to Gibbs Free Energy= 0.498136
Sum of electronic and zero-point Energies= -1377.010600
Sum of electronic and thermal Energies= -1376.981651
Sum of electronic and thermal Enthalpies= -1376.980706
Sum of electronic and thermal Free Energies= -1377.070252

Standard orientation:

Center Number	Atomic Number	Atomic Type	Coordinates (Angstroms) X	Y	Z
1	6	0	2.894635	-0.071937	0.641100
2	6	0	2.997763	-1.553856	1.085165
3	6	0	3.255754	-2.329543	-0.240227
4	6	0	3.232790	-1.208918	-1.300972
5	6	0	3.913176	-0.019664	-0.559227
6	6	0	1.544293	0.076776	-0.112921
7	6	0	1.769678	-0.735504	-1.423092
8	6	0	3.915907	1.294701	-1.359099
9	6	0	5.371001	-0.288925	-0.154953
10	6	0	3.083328	0.931330	1.766537
11	8	0	0.443615	-0.376759	0.678643
12	1	0	1.376483	1.130898	-0.341446
13	14	0	-1.156462	0.061679	0.565836
14	6	0	-1.314783	1.729746	-0.322922
15	6	0	-1.821885	0.016971	2.364822
16	6	0	-2.079766	-1.228612	-0.464791

17	6	0	-1.398926	2.964491	0.335977
18	6	0	-1.466158	4.164100	-0.366381
19	6	0	-1.445753	4.160688	-1.756502
20	6	0	-1.360484	2.950556	-2.436643
21	6	0	-1.299150	1.755730	-1.726931
22	6	0	-1.576408	-2.529561	-0.602113
23	6	0	-2.272480	-3.507548	-1.305904
24	6	0	-3.498810	-3.205846	-1.889097
25	6	0	-4.021354	-1.922708	-1.764093
26	6	0	-3.317558	-0.949358	-1.061356
27	6	0	-3.279654	0.513741	2.420944
28	6	0	-1.779965	-1.446738	2.856987
29	6	0	-0.949142	0.856134	3.319013
30	1	0	2.083545	-1.869361	1.585644
31	1	0	3.815572	-1.685371	1.796099
32	1	0	4.215444	-2.848900	-0.223553
33	1	0	2.490678	-3.082410	-0.439529
34	1	0	3.690274	-1.487419	-2.252655
35	1	0	1.608058	-0.113492	-2.305349
36	1	0	1.074922	-1.572734	-1.486250
37	1	0	4.588232	1.205138	-2.217195
38	1	0	2.940056	1.587777	-1.742746
39	1	0	4.288166	2.118875	-0.744735
40	1	0	5.780258	0.560576	0.398276
41	1	0	5.500076	-1.175180	0.463859
42	1	0	5.988454	-0.418413	-1.048642
43	1	0	4.059165	0.814279	2.245045
44	1	0	3.005145	1.961205	1.407023
45	1	0	2.322106	0.794419	2.537738
46	1	0	-1.414621	3.004296	1.417275
47	1	0	-1.534862	5.101430	0.173749
48	1	0	-1.497818	5.093599	-2.305742
49	1	0	-1.345914	2.935787	-3.520482
50	1	0	-1.244904	0.825404	-2.281165
51	1	0	-0.624730	-2.783511	-0.149643
52	1	0	-1.858035	-4.505035	-1.398796
53	1	0	-4.043251	-3.965175	-2.438465
54	1	0	-4.975414	-1.678328	-2.217202
55	1	0	-3.740649	0.046779	-0.988146
56	1	0	-3.375766	1.557573	2.112973

57	1	0	-3.937986	-0.084150	1.785602
58	1	0	-3.660977	0.437054	3.445950
59	1	0	-0.765821	-1.852888	2.833503
60	1	0	-2.419695	-2.099466	2.260151
61	1	0	-2.131367	-1.496529	3.894242
62	1	0	0.089308	0.521749	3.306970
63	1	0	-0.958532	1.921357	3.081296
64	1	0	-1.320351	0.753264	4.345567

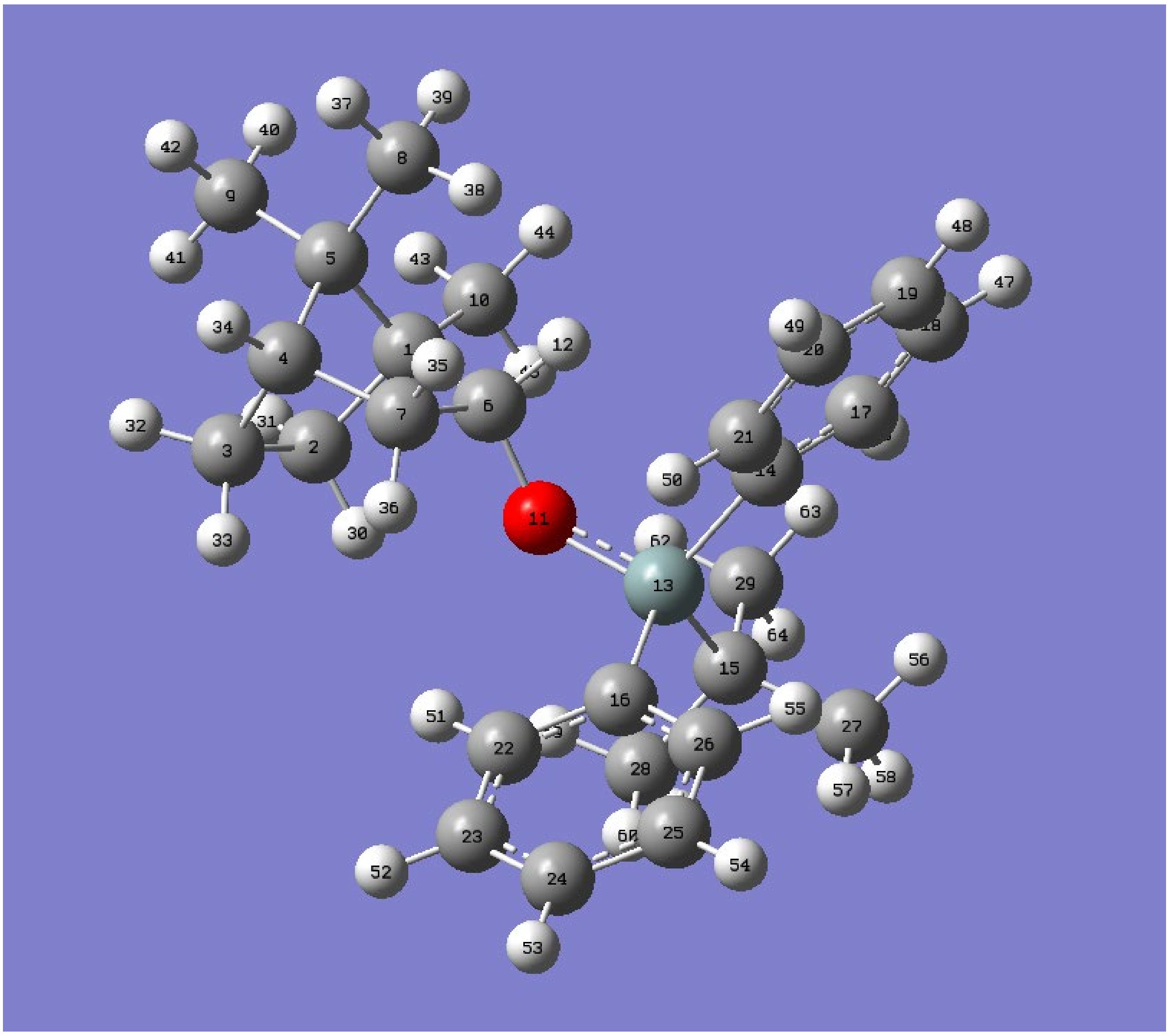

Compound bornyl TBDPS **1f**

	In the gas phase	In chloroform	In benzene
**Energy**	E(RB3LYP) -1377.56831236 a.u.	E(RB3LYP) -1377.57128366 a.u.	E(RB3LYP) -1377.57009743 a.u.
	E(RmPW1PW91) -1377.30452511 a.u.	E(RmPW1PW91) -1377.30668571 a.u.	E(RmPW1PW91) -1377.30531203 a.u.
	E(RwB97XD) -1377.22521783 a.u.	E(RwB97XD) -1377.22611116 a.u.	E(RwB97XD) -1377.22472466 a.u.

Reference: TMS B3LYP/6-311+G(2d,p) GIAO

Reference shielding: 31.8821 ppm

^{1}H NMR chemical shifts (ppm), GIAO//6-311+G(2d,p)

Atom	Atom	In the gas phase			In chloroform			In benzene		
label	Assignment	B3LYP	mPW1PW91	wB97XD	B3LYP	mPW1PW91	wB97XD	B3LYP	mPW1PW91	wB97XD
12-H	2-exo	3.6514	3.6782	3.4878	3.6529	3.7667	3.5797	3.6546	3.7708	3.5840
30-H	6-endo	2.4087	2.4838	2.4783	2.3483	2.4361	2.3221	2.3744	2.4621	2.3506
31-H	6-exo	1.2662	1.3136	1.3089	1.2912	1.3876	1.3140	1.2795	1.3745	1.3002
32-H	5-exo	1.7692	1.8071	1.7410	1.7785	1.8584	1.7732	1.7739	1.8525	1.7664
33-H	5-endo	1.3771	1.4267	1.4074	1.3904	1.4913	1.3911	1.3841	1.4833	1.3836
34-H	4-exo	1.3553	1.3721	1.2899	1.3832	1.5124	1.4124	1.3712	1.4998	1.3987
35-H	3-exo	1.8026	1.8115	1.5328	1.8359	1.9201	1.8193	1.8226	1.9062	1.8050
36-H	3-endo	1.2056	1.2445	1.1918	1.1845	1.2784	1.1838	1.1935	1.2878	1.1940
37-H	9-CH3	0.4320	0.4732	0.4355	0.4483	0.5500	0.4950	0.4403	0.5402	0.4843
38-H	9-CH3	0.4447	0.4845	0.3124	0.4361	0.5080	0.3776	0.4423	0.5158	0.3862
39-H	9-CH3	0.3912	0.4430	0.4633	0.3999	0.4985	0.4402	0.3964	0.4936	0.4354
40-H	8-CH3	0.6142	0.6771	0.6692	0.6222	0.7223	0.6732	0.6190	0.7178	0.6687
41-H	8-CH3	1.0518	1.1312	1.0585	1.0544	1.1354	1.0670	1.0525	1.1329	1.0648
42-H	8-CH3	0.5211	0.5748	0.5837	0.5370	0.6429	0.6132	0.5300	0.6345	0.6041
43-H	10-CH3	0.5449	0.5957	0.6360	0.5680	0.6655	0.6226	0.5563	0.6517	0.6078
44-H	10-CH3	0.4306	0.4998	0.5584	0.4487	0.5541	0.4750	0.4419	0.5474	0.4680
45-H	10-CH3	1.2427	1.3047	1.3161	1.2036	1.2981	1.2283	1.2205	1.3147	1.2459
46-H	14-o-17	8.3200	8.5017	8.4651	8.3764	8.5866	8.5280	8.3521	8.5603	8.5022
47-H	15-m-18	7.6237	7.7891	7.7282	7.7258	7.9431	7.9008	7.6849	7.8973	7.8536
48-H	16-p-19	7.6194	7.7918	7.7298	7.7302	7.9553	7.9170	7.6865	7.9065	7.8667
49-H	15-m-20	7.4968	7.6603	7.5807	7.5947	7.8066	7.7651	7.5566	7.7642	7.7215
50-H	14-o-21	7.6491	7.8730	7.8331	7.6732	7.8820	7.8235	7.6658	7.8749	7.8165
51-H	14-o-22	8.2538	8.4499	8.5389	8.3026	8.5138	8.4737	8.2833	8.4938	8.4550
52-H	15-m-23	7.6382	7.8032	7.7893	7.7300	7.9473	7.9089	7.6927	7.9051	7.8658
53-H	16-p-24	7.5902	7.7592	7.7252	7.6913	7.9160	7.8933	7.6505	7.8702	7.8463
54-H	15-m-25	7.4447	7.6168	7.6020	7.5402	7.7556	7.7221	7.5020	7.7130	7.6782
55-H	14-o-26	7.4444	7.6293	7.6601	7.4987	7.6969	7.6380	7.4791	7.6775	7.6186
56-H	12-TB-27	1.2156	1.2850	1.2817	1.2367	1.3387	1.2718	1.2272	1.3295	1.2629
57-H	12-TB-27	0.8512	0.9117	0.9205	0.8266	0.9283	0.8201	0.8395	0.9427	0.8345
58-H	12-TB-27	0.4996	0.5699	0.6164	0.5174	0.6434	0.6122	0.5074	0.6295	0.5975
59-H	12-TB-28	1.4049	1.4861	1.3857	1.3865	1.4924	1.3991	1.3946	1.4996	1.4081
60-H	12-TB-28	0.5810	0.6546	0.4685	0.5368	0.6437	0.5401	0.5585	0.6682	0.5646
61-H	12-TB-28	0.3352	0.4108	0.3866	0.3639	0.4921	0.4474	0.3487	0.4728	0.4269
62-H	12-TB-29	1.7928	1.8934	1.8271	1.7600	1.8651	1.7687	1.7760	1.8820	1.7869
63-H	12-TB-29	1.6166	1.6861	1.6076	1.6716	1.7706	1.6913	1.6482	1.7469	1.6674
64-H	12-TB-29	0.7891	0.8735	0.8603	0.8014	0.9296	0.8812	0.7935	0.9177	0.8682

Reference: TMS B3LYP/6-311+G(2d,p) GIAO

Reference shielding: 182.466 ppm

^{13}C NMR chemical shifts (ppm), GIAO//6-311+G(2d,p)

Atom	Atom	In the gas phase			In chloroform			In benzene		
label	Assignment	B3LYP	mPW1PW91	wB97XD	B3LYP	mPW1PW91	wB97XD	B3LYP	mPW1PW91	wB97XD
1-C	1	56.0438	48.0251	47.4623	56.0661	50.5588	49.6044	56.0561	50.5474	49.5933
2-C	6	29.3927	23.2232	22.9284	29.2693	23.8349	23.1479	29.3186	23.8908	23.1967
3-C	5	30.9945	24.9891	23.9594	30.7340	25.5100	24.6217	30.8411	25.6235	24.7304
4-C	4	50.5362	43.3822	42.2274	50.4238	44.6359	42.8434	50.4704	44.6878	42.8936
5-C	7	53.8051	45.6046	44.6027	53.8692	48.1486	46.7344	53.8406	48.1165	46.7054
6-C	2	84.1755	76.0880	74.7080	84.2498	78.2072	76.9137	84.2093	78.1574	76.8594
7-C	3	40.6666	34.3033	33.3641	40.5632	35.0000	34.5085	40.6044	35.0446	34.5485
8-C	9-CH3	18.9324	13.8507	13.7085	18.6516	13.9185	13.4247	18.7641	14.0346	13.5398
9-C	8-CH3	20.5107	15.5015	15.2468	20.2169	15.4915	15.0882	20.3347	15.6119	15.2078
10-C	10-CH3	14.2823	9.4387	9.4008	14.0777	9.5652	9.1391	14.1529	9.6444	9.2137
14-C	13-ipso-14	140.0473	133.1073	132.4670	140.0367	134.3701	134.0639	140.0519	134.4070	134.1077
15-C	11-TB	27.0923	18.7250	16.2120	27.0026	21.2063	19.2390	27.0370	21.2373	19.2701
16-C	13-ipso-16	141.9430	135.1355	135.7925	142.2994	136.6038	136.5706	142.1691	136.4941	136.4662
17-C	14-o-14	140.5620	135.3018	135.3697	140.6773	136.2149	136.2639	140.6225	136.1636	136.2173
18-C	15-m-14	131.4988	126.2664	126.2149	131.6574	127.2854	127.4745	131.5800	127.1816	127.3629
19-C	16-p-14	133.8941	128.9595	129.2030	134.2141	130.0234	130.2367	134.0792	129.8587	130.0633
20-C	15-m-14	131.5984	126.3838	126.5491	131.7444	127.3795	127.6681	131.6841	127.2988	127.5802
21-C	14-o-14	142.4534	137.3966	138.2889	142.5532	137.9753	138.1495	142.5258	137.9572	138.1369
22-C	14-o-16	141.8981	136.6717	137.3096	142.0023	137.3212	137.4794	141.9665	137.2897	137.4572
23-C	15-m-16	130.9637	125.7909	126.4023	130.9814	126.6410	126.9397	130.9580	126.5936	126.8858
24-C	16-p-16	133.0473	128.0859	128.5622	133.1875	129.0229	129.3890	133.1160	128.9234	129.2773
25-C	15-m-16	130.8193	125.6175	126.0592	130.8722	126.5586	126.8020	130.8385	126.5046	126.7345
26-C	14-o-16	140.3584	135.1540	134.8238	140.5432	135.9056	135.9901	140.4831	135.8563	135.9412
27-C	12-TB	25.3872	20.1692	19.4733	25.1323	20.2431	19.6610	25.2354	20.3509	19.7684
28-C	12-TB	28.3425	23.3070	22.9732	28.1785	23.1629	22.5926	28.2366	23.2259	22.6491
29-C	12-TB	27.8504	22.5787	21.7148	27.4908	22.6260	21.9187	27.6386	22.7839	22.0731

Table S18. Optimized coordinates, energies, and calculated NMR chemical shifts of bornyl TPS **1d**

Optimized coordinates in the gas phase

Calculation Method	RB3LYP
Basis Set	6-311+G(2d,p)
Charge	0
Spin	Singlet
E(RB3LYP)	-1451.38949639 a.u.

Zero-point correction= 0.527161 (Hartree/Particle)
Thermal correction to Energy= 0.555207
Thermal correction to Enthalpy= 0.556152
Thermal correction to Gibbs Free Energy= 0.466091
Sum of electronic and zero-point Energies= -1450.862336
Sum of electronic and thermal Energies= -1450.834289
Sum of electronic and thermal Enthalpies= -1450.833345
Sum of electronic and thermal Free Energies= -1450.923405

Standard orientation:

Center Number	Atomic Number	Atomic Type	Coordinates (Angstroms) X	Y	Z
1	6	0	2.902049	-0.588341	-0.756979
2	6	0	2.948655	0.235216	-2.070341
3	6	0	3.502387	1.622170	-1.628651
4	6	0	3.667292	1.452385	-0.103443
5	6	0	4.145775	-0.024748	0.027649
6	6	0	1.737271	-0.011764	0.093183
7	6	0	2.246972	1.403442	0.497153
8	6	0	4.277652	-0.511370	1.481410
9	6	0	5.494044	-0.305934	-0.653142
10	6	0	2.821024	-2.091529	-0.964828
11	8	0	0.506245	-0.005307	-0.633660
12	1	0	1.599177	-0.637636	0.976811
13	14	0	-1.034311	-0.013212	-0.018642
14	6	0	-1.676828	1.749797	0.159377
15	6	0	-2.063723	-0.930896	-1.296276
16	6	0	-1.089622	-0.894728	1.648154

17	6	0	-2.637613	2.108865	1.114092
18	6	0	-3.150903	3.401774	1.173133
19	6	0	-2.709908	4.367349	0.275025
20	6	0	-1.756927	4.033084	-0.682713
21	6	0	-1.250380	2.739393	-0.738146
22	6	0	-1.447983	-2.247207	1.734344
23	6	0	-1.441236	-2.921556	2.951717
24	6	0	-1.074344	-2.254367	4.115462
25	6	0	-0.716585	-0.910953	4.055818
26	6	0	-0.726796	-0.242182	2.836213
27	6	0	-3.463359	-0.858119	-1.274270
28	6	0	-4.236833	-1.549099	-2.200191
29	6	0	-3.622469	-2.326576	-3.177730
30	6	0	-2.234891	-2.406536	-3.222623
31	6	0	-1.466029	-1.715629	-2.290067
32	1	0	1.957513	0.302250	-2.516517
33	1	0	3.600010	-0.246124	-2.802122
34	1	0	4.453010	1.850428	-2.113561
35	1	0	2.819228	2.439503	-1.867343
36	1	0	4.319628	2.195916	0.359166
37	1	0	2.250336	1.529057	1.581525
38	1	0	1.602411	2.179208	0.083893
39	1	0	5.116402	-0.004674	1.967283
40	1	0	3.395120	-0.335854	2.094324
41	1	0	4.489435	-1.583489	1.511747
42	1	0	5.754753	-1.364131	-0.567998
43	1	0	5.515070	-0.047635	-1.710407
44	1	0	6.287961	0.261875	-0.159222
45	1	0	3.673884	-2.461462	-1.539834
46	1	0	2.795699	-2.630187	-0.013613
47	1	0	1.915294	-2.357845	-1.514865
48	1	0	-2.990755	1.373875	1.829169
49	1	0	-3.892171	3.655192	1.922460
50	1	0	-3.106124	5.375198	0.320531
51	1	0	-1.410153	4.780700	-1.387004
52	1	0	-0.511939	2.494013	-1.493440
53	1	0	-1.742374	-2.782748	0.838673
54	1	0	-1.725593	-3.966813	2.991690
55	1	0	-1.070005	-2.777049	5.064991
56	1	0	-0.432810	-0.383794	4.959548

57	1	0	-0.453201	0.807413	2.815209
58	1	0	-3.962775	-0.246767	-0.529774
59	1	0	-5.317799	-1.477098	-2.163426
60	1	0	-4.223095	-2.863154	-3.903151
61	1	0	-1.750193	-3.005124	-3.985556
62	1	0	-0.385689	-1.777276	-2.338881

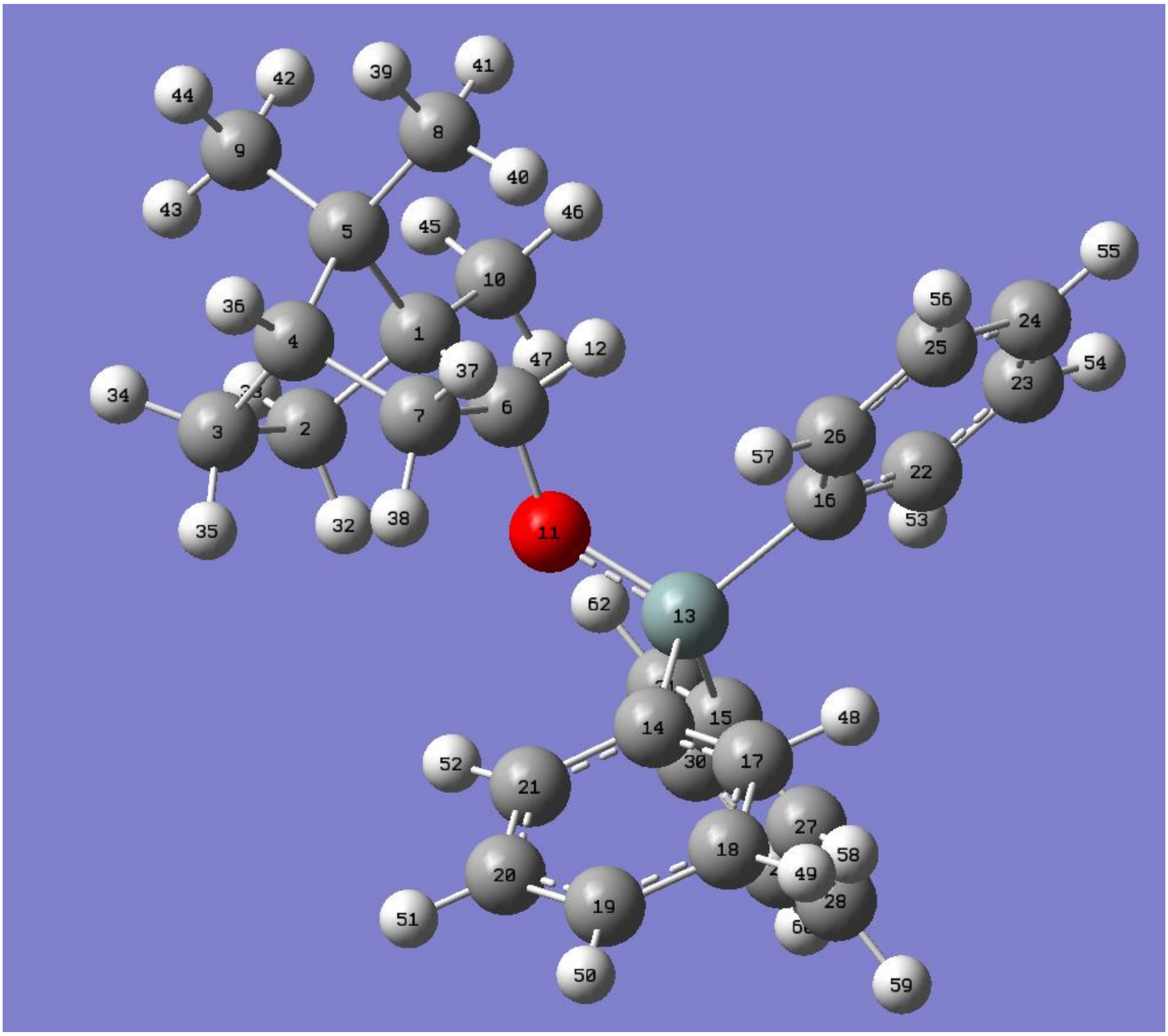

Compound bornyl TPS **1g**

	In the gas phase	In chloroform	In benzene
**Energy**	E(RB3LYP) -1451.38949609 a.u.	E(RB3LYP) -1451.39379974 a.u.	E(RB3LYP) -1451.39206519 a.u.
	E(RmPW1PW91) -1451.10467103 a.u.	E(RmPW1PW91) -1451.10961547 a.u.	E(RmPW1PW91) -1451.10762126 a.u.
	E(RwB97XD) -1451.00104380 a.u.	E(RwB97XD) -1451.00603047 a.u.	E(RwB97XD) -1451.00401649 a.u.

Reference: TMS B3LYP/6-311+G(2d,p) GIAO

Reference shielding: 31.8821 ppm

^{1}H NMR chemical shifts (ppm), GIAO//6-311+G(2d,p)

Atom	Atom	In the gas phase			In chloroform			In benzene		
label	Assignment	B3LYP	mPW1PW91	wB97XD	B3LYP	mPW1PW91	wB97XD	B3LYP	mPW1PW91	wB97XD
12-H	2-exo	4.0185	4.1427	3.9694	4.0253	4.1458	3.9714	4.0239	4.1462	3.9723
32-H	6-endo	2.5412	2.6291	2.5172	2.4647	2.5522	2.4363	2.4986	2.5863	2.4722
33-H	6-exo	1.3463	1.4381	1.351	1.3825	1.4776	1.3915	1.3657	1.4591	1.3725
34-H	5-exo	1.7806	1.8607	1.7816	1.7953	1.8782	1.8007	1.7884	1.87	1.7917
35-H	5-endo	1.3815	1.4788	1.376	1.3843	1.4852	1.3817	1.3835	1.4829	1.3798
36-H	4-exo	1.4084	1.5307	1.4399	1.4386	1.5627	1.4741	1.4255	1.5488	1.4592
37-H	3-exo	1.9724	2.0573	1.9731	2.0233	2.1111	2.0278	2.0011	2.0877	2.004
38-H	3-endo	1.237	1.336	1.2338	1.2026	1.3005	1.1965	1.2182	1.3165	1.2133
39-H	9-CH3	0.5655	0.664	0.5896	0.5836	0.6863	0.6137	0.5749	0.6758	0.6024
40-H	9-CH3	0.6383	0.7203	0.612	0.635	0.7146	0.605	0.6369	0.7176	0.6086
41-H	9-CH3	0.4931	0.5872	0.5133	0.5007	0.5978	0.5237	0.4988	0.5946	0.5206
42-H	8-CH3	0.6513	0.7494	0.6929	0.6619	0.7629	0.7061	0.6576	0.7573	0.7006
43-H	8-CH3	1.0959	1.1747	1.0925	1.0979	1.1777	1.0948	1.0967	1.1759	1.0933
44-H	8-CH3	0.593	0.6981	0.665	0.6108	0.7196	0.6877	0.6028	0.7101	0.6776
45-H	10-CH3	0.5765	0.6705	0.6347	0.6053	0.7035	0.6696	0.5908	0.6868	0.652
46-H	10-CH3	0.3552	0.4574	0.3796	0.3634	0.4652	0.3874	0.363	0.4654	0.3876
47-H	10-CH3	1.2474	1.3478	1.2793	1.2224	1.3245	1.2541	1.2323	1.3338	1.2642
48-H	12-o-14	7.7786	7.9807	7.9255	7.8356	8.0388	7.9839	7.8126	8.0154	7.9604
49-H	13-m-14	7.5383	7.7439	7.7065	7.6451	7.8626	7.8282	7.6009	7.8135	7.7778
50-H	14-p-14	7.6097	7.8248	7.7887	7.7231	7.9514	7.9186	7.6769	7.8998	7.8655
51-H	13-m-14	7.6059	7.8081	7.7517	7.7067	7.921	7.8669	7.6664	7.8758	7.8207
52-H	12-o-14	8.1761	8.3931	8.349	8.2204	8.4403	8.3941	8.2062	8.425	8.3799
53-H	12-o-16	7.3535	7.5696	7.516	7.3421	7.5577	7.5034	7.3498	7.5659	7.5118
54-H	13-m-16	7.4955	7.6926	7.6296	7.5877	7.7965	7.7356	7.5521	7.7561	7.6943
55-H	14-p-16	7.6164	7.8316	7.7967	7.7345	7.9632	7.9315	7.6873	7.9103	7.8773
56-H	13-m-16	7.6014	7.8093	7.765	7.7185	7.9388	7.8976	7.671	7.8861	7.8436
57-H	12-o-16	7.959	8.1654	8.1131	8.0294	8.2379	8.1851	8.001	8.2088	8.1562
58-H	12-o-15	7.371	7.5773	7.5098	7.4271	7.6339	7.5664	7.4064	7.6134	7.5459
59-H	13-m-15	7.3947	7.5971	7.5381	7.5012	7.7156	7.6601	7.4577	7.6673	7.6103
60-H	14-p-15	7.5643	7.778	7.746	7.6806	7.9082	7.8791	7.6322	7.854	7.8237
61-H	13-m-15	7.6579	7.8595	7.8262	7.7651	7.9793	7.949	7.7204	7.9294	7.8977
62-H	12-o-15	8.4645	8.6758	8.6351	8.5121	8.7263	8.6835	8.4927	8.7056	8.6638

Reference: TMS B3LYP/6-311+G(2d,p) GIAO

Reference shielding: 182.466 ppm

^{13}C NMR chemical shifts (ppm), GIAO//6-311+G(2d,p)

Atom	Atom	In the gas phase			In chloroform			In benzene		
label	Assignment	B3LYP	mPW1PW91	wB97XD	B3LYP	mPW1PW91	wB97XD	B3LYP	mPW1PW91	wB97XD
1-C	1	56.1436	50.6568	49.6003	56.1760	50.6944	49.6353	56.1624	50.6787	49.6207
2-C	6	29.6393	24.1638	23.3100	29.5180	24.0259	23.1890	29.5669	24.0815	23.2376
3-C	5	31.1579	25.8734	25.0377	30.9252	25.6237	24.7992	31.0188	25.7238	24.8944
4-C	4	50.5120	44.7250	43.0715	50.3909	44.5905	42.9410	50.4419	44.6461	42.9950
5-C	7	53.9359	48.2353	46.9770	53.9962	48.3036	47.0362	53.9699	48.2737	47.0102
6-C	2	83.4463	77.4507	76.0887	83.6139	77.6418	76.2852	83.5320	77.5489	76.1893
7-C	3	40.8608	35.2311	34.6490	40.7544	35.1151	34.5436	40.7951	35.1595	34.5833
8-C	9	19.0570	14.3497	13.8862	18.7665	14.0496	13.5879	18.8855	14.1721	13.7099
9-C	8	20.6010	15.8807	15.4665	20.3117	15.5845	15.1711	20.4264	15.7015	15.2881
10-C	10	14.0648	9.5870	9.1600	13.8319	9.3435	8.9263	13.9224	9.4380	9.0166
14-C	11-ipso-14	142.4758	136.7623	136.6129	142.5887	136.8061	136.6489	142.5532	136.8027	136.6486
15-C	11-ipso-15	141.2426	135.4138	135.4634	141.6474	135.7543	135.8132	141.5149	135.6515	135.7074
16-C	11-ipso-16	139.5934	133.9182	133.7783	139.5691	133.8371	133.6856	139.6007	133.8967	133.7501
17-C	12-o-14	140.1765	135.6252	135.6535	140.4730	135.9150	135.9428	140.3453	135.7904	135.8186
18-C	13-m-14	131.0669	126.6728	126.8910	131.2744	126.9383	127.1818	131.1596	126.7975	127.0296
19-C	14-p-14	133.5565	129.3226	129.5679	133.8796	129.7248	129.9948	133.7308	129.5412	129.8008
20-C	13-m-14	131.9253	127.4595	127.6068	132.0318	127.6276	127.7943	131.9811	127.5498	127.7082
21-C	12-o-14	141.0095	136.4469	136.5858	140.9228	136.3415	136.4575	140.9989	136.4272	136.5546
22-C	12-o-16	142.2516	137.9431	138.0724	141.8678	137.5233	137.6350	142.0439	137.7169	137.8364
23-C	13-m-16	132.4493	128.0301	128.1363	132.5034	128.1410	128.2673	132.4827	128.0947	128.2128
24-C	14-p-16	134.0701	129.8500	130.1029	134.4614	130.3153	130.5904	134.2889	130.1091	130.3748
25-C	13-m-16	130.6966	126.2866	126.5294	131.0692	126.7197	126.9833	130.8964	126.5195	126.7745
26-C	12-o-16	140.4181	135.7936	136.0970	140.8207	136.1903	136.4809	140.6487	136.0212	136.3176
27-C	12-o-15	140.0214	135.6039	135.6481	139.9718	135.5139	135.5508	140.0238	135.5865	135.6267
28-C	13-m-15	131.4836	127.0448	127.1453	131.7307	127.3518	127.4802	131.6106	127.2061	127.3218
29-C	14-p-15	133.4490	129.2184	129.4871	133.7630	129.6141	129.8941	133.6026	129.4175	129.6919
30-C	13-m-15	131.2854	126.8739	127.1031	131.5056	127.1647	127.4161	131.3837	127.0105	127.2521
31-C	12-o-15	139.8403	135.4237	135.7030	139.8366	135.4210	135.6752	139.8431	135.4265	135.6928

Table S19. Optimized coordinates, energies, and calculated NMR chemical shifts of isobornyl TBDMS **2d**

Optimized coordinates in the gas phase

Calculation Method	RB3LYP
Basis Set	6-311+G(2d,p)
Charge	0
Spin	Singlet
E(RB3LYP)	-994.00716090 a.u.

Zero-point correction=	0.449065 (Hartree/Particle)
Thermal correction to Energy=	0.471953
Thermal correction to Enthalpy=	0.472897
Thermal correction to Gibbs Free Energy=	0.398409
Sum of electronic and zero-point Energies=	-993.558096
Sum of electronic and thermal Energies=	-993.535208
Sum of electronic and thermal Enthalpies=	-993.534264
Sum of electronic and thermal Free Energies=	-993.608752

Standard orientation:

Center Number	Atomic Number	Atomic Type	Coordinates (Angstroms) X	Y	Z
1	6	0	1.929171	-0.072979	0.780497
2	6	0	3.001939	-1.185814	0.947519
3	6	0	3.645796	-1.313037	-0.465094
4	6	0	2.861472	-0.266931	-1.285580
5	6	0	2.631153	0.880595	-0.257733
6	6	0	0.809097	-0.751420	-0.067630
7	6	0	1.440329	-0.829263	-1.488649
8	6	0	1.756730	2.028508	-0.787587
9	6	0	3.932469	1.521248	0.253774
10	6	0	1.453245	0.526286	2.091597
11	8	0	-0.419579	-0.023983	-0.065283
12	1	0	0.625072	-1.752825	0.336617
13	14	0	-1.931916	-0.711627	0.083034
14	6	0	-2.185194	-2.017069	-1.251850
15	6	0	-2.102481	-1.544641	1.764210
16	6	0	-3.149107	0.744789	-0.110305

17	6	0	-2.983778	1.385199	-1.502248
18	6	0	-4.598609	0.243285	0.044589
19	6	0	-2.866153	1.810964	0.966032
20	1	0	2.553061	-2.121033	1.290914
21	1	0	3.737798	-0.898681	1.700266
22	1	0	4.713920	-1.092419	-0.440687
23	1	0	3.538459	-2.314659	-0.886441
24	1	0	3.352438	0.030364	-2.214383
25	1	0	1.453444	-1.847034	-1.884720
26	1	0	0.852330	-0.218917	-2.173601
27	1	0	2.271060	2.537481	-1.608622
28	1	0	0.780990	1.706736	-1.139458
29	1	0	1.588891	2.771835	-0.003474
30	1	0	3.716405	2.260797	1.029224
31	1	0	4.646343	0.811187	0.668225
32	1	0	4.432431	2.050241	-0.562729
33	1	0	0.690020	1.288137	1.929649
34	1	0	1.018865	-0.242392	2.737963
35	1	0	2.283570	0.980417	2.638586
36	1	0	-2.080426	-1.599000	-2.255414
37	1	0	-1.451280	-2.821693	-1.151267
38	1	0	-3.175994	-2.473321	-1.177886
39	1	0	-1.969859	-0.835154	2.583859
40	1	0	-1.355428	-2.333836	1.886445
41	1	0	-3.084740	-2.011382	1.876435
42	1	0	-3.668612	2.234619	-1.611194
43	1	0	-1.968443	1.756417	-1.656835
44	1	0	-3.210647	0.680813	-2.306977
45	1	0	-5.300540	1.077627	-0.069543
46	1	0	-4.778398	-0.197942	1.028679
47	1	0	-4.859134	-0.503396	-0.710639
48	1	0	-1.848489	2.199083	0.888949
49	1	0	-3.555602	2.655914	0.851090
50	1	0	-3.000532	1.418334	1.977506

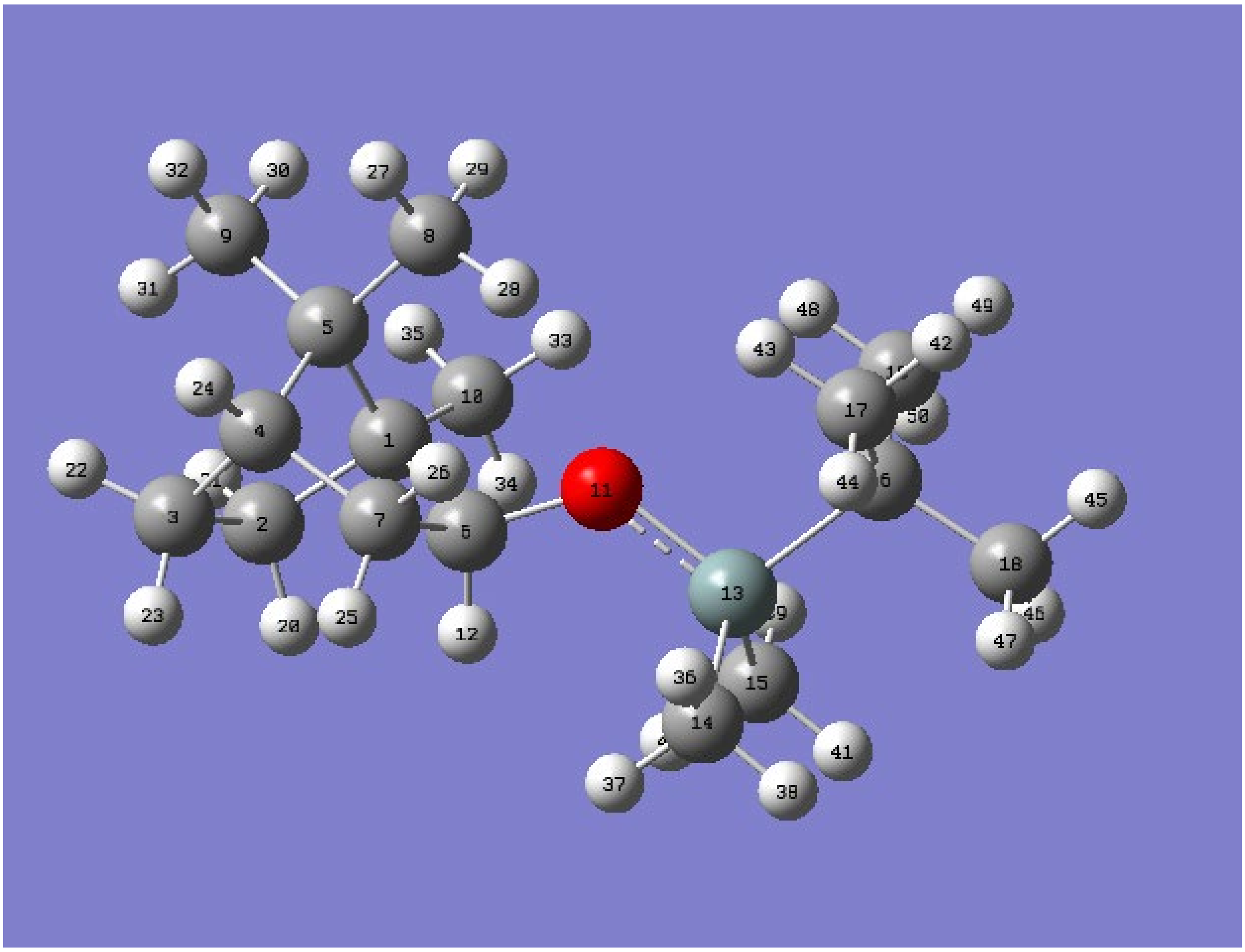
32
30
27
29
9
8
31
28
48
49
5
35
33
43
42
24
10
17
50
1
22
4
26
34
11
44
45
3
2
7
6
18
13
23
20
25
12
47
36
15
14
41
37
38

Compound isobornyl TBDMS **2d**

	In the gas phase	In chloroform	In benzene
**Energy**	E(RB3LYP) -994.00716090 a.u.	E(RB3LYP) -994.00828409 a.u.	E(RB3LYP) -994.00781689 a.u.
	E(RmPW1PW91) -993.83570584 a.u.	E(RmPW1PW91) -993.83596435 a.u.	E(RmPW1PW91) -993.83544523 a.u.
	E(RwB97XD) -993.79573498 a.u.	E(RwB97XD) -993.79531587 a.u.	E(RwB97XD) -993.79479435 a.u.

Reference: TMS B3LYP/6-311+G(2d,p) GIAO

Reference shielding: 31.8821 ppm

^{1}H NMR chemical shifts (ppm), GIAO//6-311+G(2d,p)

Atom	Atom	In the gas phase			In chloroform			In benzene		
label	Assignment	B3LYP	mPW1PW91	wB97XD	B3LYP	mPW1PW91	wB97XD	B3LYP	mPW1PW91	wB97XD
12-H	2-endo	3.5522	3.5796	3.4469	3.6251	3.7331	3.5848	3.5921	3.7006	3.5522
20-H	6-endo	0.8263	0.8634	0.7893	0.8681	0.9600	0.8742	0.8484	0.9397	0.8539
21-H	6-exo	1.4905	1.5528	1.4522	1.4956	1.5835	1.4892	1.4945	1.5815	1.4873
22-H	5-exo	1.6937	1.7522	1.6595	1.6925	1.7709	1.6918	1.6938	1.7712	1.6918
23-H	5-endo	0.9027	0.9490	0.8710	0.9230	1.0166	0.9273	0.9135	1.0061	0.9163
24-H	4-exo	1.5137	1.5649	1.5176	1.5291	1.6612	1.5792	1.5236	1.6557	1.5731
25-H	3-endo	1.5472	1.5939	1.5408	1.5931	1.6933	1.6179	1.5729	1.6717	1.5955
26-H	3-exo	1.7155	1.7914	1.7079	1.6833	1.7873	1.6575	1.6993	1.8036	1.6753
27-H	9-CH3	0.6747	0.7292	0.6784	0.6759	0.7798	0.7213	0.6739	0.7763	0.7172
28-H	9-CH3	1.7512	1.8704	1.8562	1.7263	1.7961	1.7063	1.7384	1.8093	1.7213
29-H	9-CH3	0.7041	0.7595	0.7224	0.6886	0.7952	0.7309	0.6960	0.8013	0.7377
30-H	8-CH3	0.7220	0.7783	0.7271	0.7137	0.8087	0.7562	0.7178	0.8117	0.7596
31-H	8-CH3	0.8527	0.9310	0.8642	0.8821	0.9584	0.8918	0.8692	0.9452	0.8784
32-H	8-CH3	0.6864	0.7492	0.7383	0.6791	0.7866	0.7674	0.6821	0.7886	0.7690
33-H	10-CH3	1.1645	1.2226	1.1885	1.1178	1.2088	1.1269	1.1395	1.2309	1.1510
34-H	10-CH3	0.5746	0.6271	0.6211	0.5871	0.7031	0.6457	0.5806	0.6966	0.6387
35-H	10-CH3	0.5331	0.5921	0.6043	0.5446	0.6507	0.6156	0.5393	0.6437	0.6078
36-H	11-CH3-14	-0.0401	0.0468	-0.0337	-0.0550	0.0750	-0.0154	-0.0462	0.0824	-0.0072
37-H	11-CH3-14	-0.0129	0.1009	0.0541	0.0214	0.1638	0.0829	0.0049	0.1465	0.0649
38-H	11-CH3-14	-0.2074	-0.1353	-0.2164	-0.1813	-0.0655	-0.1293	-0.1931	-0.0791	-0.1437
39-H	11-CH3-15	-0.0889	0.0080	0.0242	-0.1067	0.0248	-0.0515	-0.0964	0.0337	-0.0414
40-H	11-CH3-15	0.0484	0.1484	0.0585	0.0869	0.2242	0.1403	0.0682	0.2044	0.1200
41-H	11-CH3-15	-0.2715	-0.2033	-0.2347	-0.2450	-0.1260	-0.1931	-0.2567	-0.1396	-0.2076
42-H	13-CH3-17	0.4941	0.5738	0.5696	0.4853	0.6074	0.5642	0.4877	0.6073	0.5633
43-H	13-CH3-17	1.2345	1.3149	1.2711	1.1910	1.2877	1.1984	1.2120	1.3095	1.2220
44-H	13-CH3-17	0.9071	0.9789	0.9074	0.9409	1.0449	0.9635	0.9256	1.0294	0.9476
45-H	13-CH3-18	0.5604	0.6440	0.6288	0.5393	0.6580	0.6142	0.5483	0.6646	0.6207
46-H	13-CH3-18	0.8093	0.8751	0.8058	0.8355	0.9314	0.8451	0.8243	0.9202	0.8338
47-H	13-CH3-18	0.7994	0.8644	0.8015	0.8282	0.9239	0.8399	0.8153	0.9109	0.8266
48-H	13-CH3-19	1.2850	1.3729	1.3906	1.2338	1.3315	1.2422	1.2589	1.3576	1.2704
49-H	13-CH3-19	0.4862	0.5640	0.5562	0.4793	0.6016	0.5606	0.4804	0.6000	0.5582
50-H	13-CH3-19	0.9327	1.0101	0.9564	0.9655	1.0707	0.9837	0.9511	1.0561	0.9687

Reference: TMS B3LYP/6-311+G(2d,p) GIAO

Reference shielding: 182.466 ppm

^{13}C NMR chemical shifts (ppm), GIAO//6-311+G(2d,p)

Atom	Atom	In the gas phase			In chloroform			In benzene		
label	Assignment	B3LYP	mPW1PW91	wB97XD	B3LYP	mPW1PW91	wB97XD	B3LYP	mPW1PW91	wB97XD
1-C	1	55.5763	47.4804	46.7459	55.5317	50.0212	49.0005	55.5523	50.0411	49.0222
2-C	6	37.2812	31.0395	29.8509	36.9643	31.2782	30.3952	37.0997	31.4159	30.5342
3-C	5	30.2107	24.1925	23.8580	29.9436	24.6520	23.9409	30.0547	24.7692	24.0590
4-C	4	50.5853	43.4495	42.1493	50.5168	44.7541	43.2149	50.5437	44.7866	43.2477
5-C	7	53.1469	45.0582	44.3308	53.1843	47.6303	46.4850	53.1662	47.6128	46.4698
6-C	2	85.0938	76.9897	75.1617	84.8549	78.6222	77.2201	84.9624	78.7224	77.3216
7-C	3	45.2113	38.8727	37.9807	45.0421	39.3168	38.5922	45.1099	39.3882	38.6588
8-C	9-CH3	20.9725	15.9575	15.8979	20.7058	15.9497	15.4152	20.8157	16.0626	15.5276
9-C	8-CH3	20.9068	15.8999	15.8944	20.6320	15.8935	15.4922	20.7402	16.0056	15.6031
10-C	10-CH3	12.5659	7.6684	8.3061	12.3296	7.7982	7.3520	12.4266	7.8985	7.4513
14-C	11-DM	-5.6824	-10.0232	-11.1524	-6.0057	-9.6888	-10.3862	-5.8767	-9.5588	-10.2557
15-C	11-DM	-6.8776	-11.0236	-10.3509	-7.1622	-10.7686	-11.4461	-7.0512	-10.6552	-11.3325
16-C	12-TB	24.6098	16.4240	14.9877	24.5235	18.7771	17.2806	24.5602	18.8141	17.3150
17-C	13-TB	25.8464	20.7309	20.3025	25.6386	20.7494	20.2232	25.7191	20.8378	20.3069
18-C	13-TB	25.9441	20.7817	19.8990	25.6065	20.6763	20.0528	25.7438	20.8186	20.1969
19-C	13-TB	25.7373	20.5955	20.4345	25.5295	20.6482	20.1109	25.6079	20.7339	20.1904

Table S20. Optimized coordinates, energies, and calculated NMR chemical shifts of isobornyl DMMPS **2e**

Optimized coordinates in the gas phase

Calculation Method	RB3LYP
Basis Set	6-311+G(2d,p)
Charge	0
Spin	Singlet
E(RB3LYP)	-1067.82505135 a.u.

Zero-point correction= 0.418796 (Hartree/Particle)
Thermal correction to Energy= 0.440594
Thermal correction to Enthalpy= 0.441538
Thermal correction to Gibbs Free Energy= 0.367736
Sum of electronic and zero-point Energies= -1067.406255
Sum of electronic and thermal Energies= -1067.384457
Sum of electronic and thermal Enthalpies= -1067.383513
Sum of electronic and thermal Free Energies= -1067.457315

Standard orientation:

Center Number	Atomic Number	Atomic Type	Coordinates (Angstroms) X	Y	Z
1	6	0	-1.867623	-0.807989	-0.430959
2	6	0	-2.191768	-2.039925	0.460333
3	6	0	-3.027565	-1.452129	1.636063
4	6	0	-3.079357	0.051666	1.291077
5	6	0	-3.177246	0.049806	-0.263269
6	6	0	-0.814492	-0.010633	0.397690
7	6	0	-1.667063	0.617883	1.540055
8	6	0	-3.128598	1.450416	-0.895606
9	6	0	-4.449045	-0.628759	-0.799682
10	6	0	-1.424225	-1.164479	-1.838464
11	8	0	-0.130287	0.970939	-0.384030
12	1	0	-0.071287	-0.710950	0.791831
13	14	0	1.417634	1.514760	-0.107196
14	6	0	1.800730	2.604506	-1.579507
15	6	0	1.521363	2.504188	1.488016
16	6	0	2.605316	0.048956	-0.028010

17	6	0	2.684376	-0.849950	-1.102453
18	6	0	3.560829	-1.928903	-1.083831
19	6	0	4.385646	-2.136286	0.018324
20	6	0	4.326107	-1.260235	1.095945
21	6	0	3.444942	-0.182149	1.069062
22	1	0	-1.278653	-2.534747	0.798869
23	1	0	-2.758797	-2.782030	-0.103911
24	1	0	-4.024582	-1.892854	1.679914
25	1	0	-2.561747	-1.623903	2.608590
26	1	0	-3.869186	0.600669	1.807815
27	1	0	-1.290728	0.359191	2.532348
28	1	0	-1.643041	1.703859	1.453088
29	1	0	-4.010393	2.024090	-0.593651
30	1	0	-2.240940	2.018038	-0.632121
31	1	0	-3.151206	1.376005	-1.986244
32	1	0	-4.426738	-0.675853	-1.891564
33	1	0	-4.603137	-1.641696	-0.431303
34	1	0	-5.329218	-0.041413	-0.522429
35	1	0	-1.180994	-0.272931	-2.417714
36	1	0	-0.532907	-1.798396	-1.816399
37	1	0	-2.205561	-1.717564	-2.366299
38	1	0	2.799332	3.040496	-1.492774
39	1	0	1.762362	2.037718	-2.512433
40	1	0	1.078635	3.421508	-1.654316
41	1	0	2.525673	2.909369	1.639611
42	1	0	1.265094	1.906377	2.366063
43	1	0	0.828071	3.348317	1.448777
44	1	0	2.047998	-0.709511	-1.970059
45	1	0	3.601944	-2.608307	-1.927596
46	1	0	5.070261	-2.976373	0.035838
47	1	0	4.964566	-1.415782	1.958140
48	1	0	3.416625	0.486617	1.922199

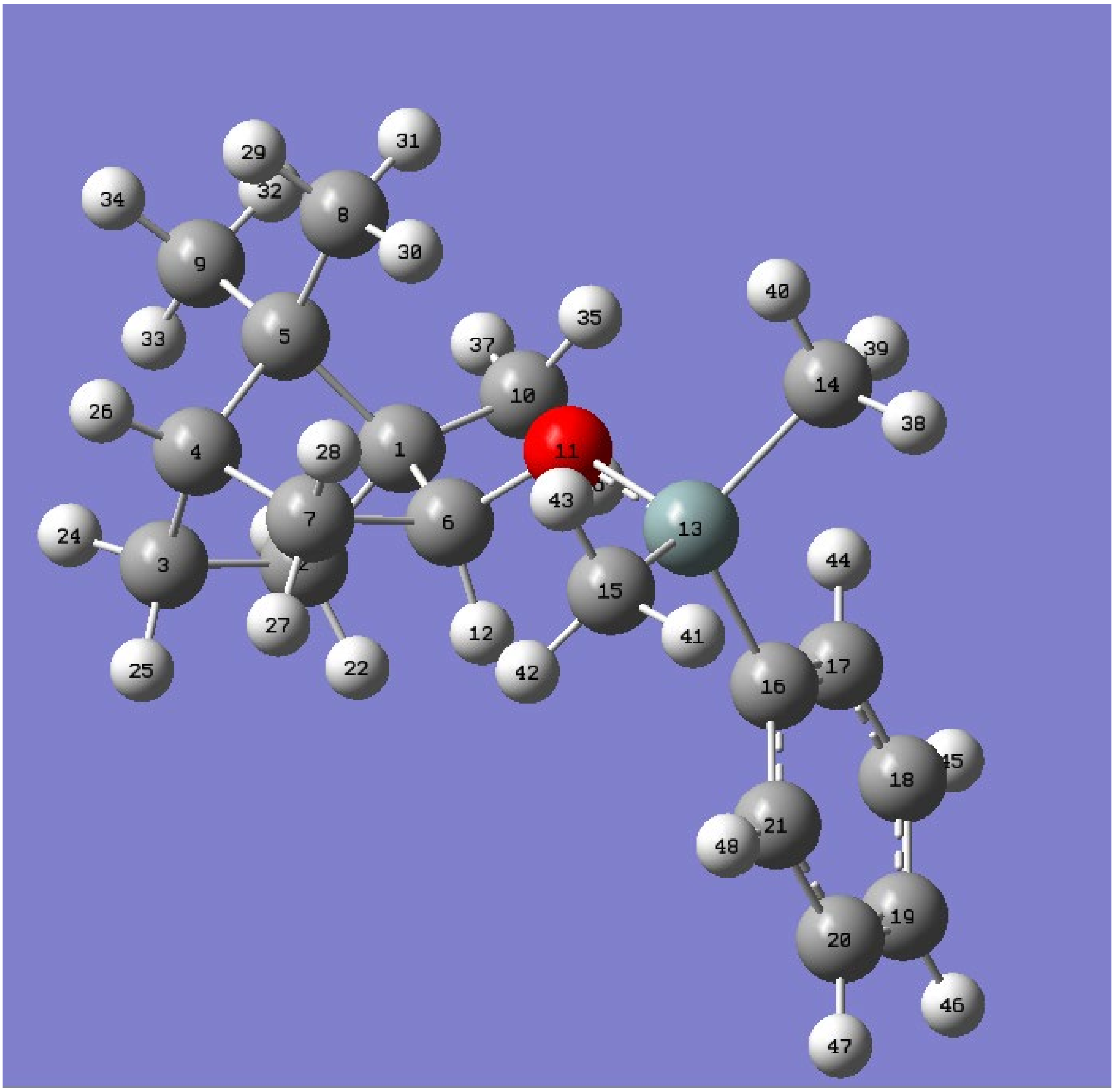
29
31
34
32
8
30
9
40
5
35
37
33
39
14
10
26
38
11
4
28
1
43
13
24
7
6
3
2
44
15
27
12
41
25
22
42
17
16
45
18
21
48
19
20
46
47

Compound isobornyl DMMPS **2e**

	In the gas phase	In chloroform	In benzene
**Energy**	E(RB3LYP) -1067.82505135 a.u.	E(RB3LYP) -1067.82756285 a.u.	E(RB3LYP) -1067.82654820 a.u.
	E(RmPW1PW91) -1067.63402773 a.u.	E(RmPW1PW91) -1067.63593841 a.u.	E(RmPW1PW91) -1067.63479321 a.u.
	E(RwB97XD) -1067.57318138 a.u.	E(RwB97XD) -1067.57442458 a.u.	E(RwB97XD) -1067.57326706 a.u.

Reference: TMS B3LYP/6-311+G(2d,p) GIAO

Reference shielding: 31.8821 ppm

^{1}H NMR chemical shifts (ppm), GIAO//6-311+G(2d,p)

Atom	Atom	In the gas phase			In chloroform			In benzene		
label	Assignment	B3LYP	mPW1PW91	wB97XD	B3LYP	mPW1PW91	wB97XD	B3LYP	mPW1PW91	wB97XD
12-H	2-endo	3.3573	3.3692	3.1830	3.3621	3.4675	3.3047	3.3617	3.4700	3.3076
22-H	6-endo	0.5426	0.5891	0.5225	0.5490	0.6351	0.5357	0.5493	0.6361	0.5371
23-H	6-exo	1.3546	1.3979	1.3439	1.3701	1.4586	1.3508	1.3628	1.4499	1.3421
24-H	5-exo	1.5517	1.6019	1.5471	1.5549	1.6340	1.5540	1.5532	1.6310	1.5506
25-H	5-endo	0.7692	0.8031	0.7512	0.7910	0.8830	0.7882	0.7815	0.8725	0.7772
26-H	4-exo	1.4402	1.4868	1.4624	1.4633	1.5960	1.5070	1.4534	1.5858	1.4962
27-H	3-endo	1.5018	1.5478	1.4890	1.5625	1.6619	1.5782	1.5363	1.6341	1.5500
28-H	3-exo	1.7582	1.8305	1.6957	1.7559	1.8622	1.7239	1.7571	1.8626	1.7251
29-H	9-CH3	0.6534	0.7089	0.6760	0.6690	0.7771	0.7314	0.6597	0.7661	0.7198
30-H	9-CH3	1.8258	1.9417	1.8756	1.7811	1.8566	1.7578	1.8011	1.8780	1.7805
31-H	9-CH3	0.7223	0.7825	0.7246	0.7058	0.8167	0.7587	0.7140	0.8234	0.7660
32-H	8-CH3	0.7116	0.7661	0.7114	0.7054	0.8044	0.7459	0.7081	0.8057	0.7476
33-H	8-CH3	0.8260	0.9015	0.7970	0.8538	0.9335	0.8597	0.8420	0.9215	0.8474
34-H	8-CH3	0.6850	0.7239	0.6919	0.6844	0.7936	0.7549	0.6838	0.7919	0.7526
35-H	10-CH3	1.3185	1.3685	1.2455	1.2671	1.3580	1.2995	1.2903	1.3807	1.3234
36-H	10-CH3	0.6911	0.7467	0.6975	0.6964	0.8090	0.7369	0.6947	0.8078	0.7358
37-H	10-CH3	0.5442	0.6157	0.5390	0.5717	0.6794	0.6343	0.5577	0.6634	0.6175
38-H	11-CH3-14	-0.2741	-0.1758	-0.1818	-0.2360	-0.0954	-0.1662	-0.2524	-0.1128	-0.1844
39-H	11-CH3-14	0.4164	0.5173	0.3926	0.4045	0.5409	0.4507	0.4120	0.5457	0.4565
40-H	11-CH3-14	0.2548	0.3465	0.2932	0.2552	0.4015	0.3191	0.2566	0.3996	0.3175
41-H	11-CH3-15	0.2729	0.3530	0.3111	0.3182	0.4409	0.3645	0.2997	0.4200	0.3423
42-H	11-CH3-15	0.4930	0.5408	0.4687	0.5277	0.6690	0.5762	0.5108	0.6504	0.5577
43-H	11-CH3-15	0.1977	0.3063	0.2263	0.2159	0.3737	0.3036	0.2105	0.3655	0.2957
44-H	13-o-17	7.9178	8.0859	8.1180	7.9792	8.1998	8.1486	7.9563	8.1753	8.1248
45-H	14-m-18	7.5825	7.7506	7.6989	7.6784	7.8913	7.8499	7.6411	7.8493	7.8071
46-H	15-p-19	7.6038	7.7686	7.7075	7.7059	7.9224	7.8608	7.6656	7.8772	7.8147
47-H	14-m-20	7.5282	7.6878	7.6294	7.6309	7.8426	7.7921	7.5895	7.7965	7.7450
48-H	13-o-21	7.7115	7.8891	7.8222	7.8040	8.0220	7.9651	7.7650	7.9804	7.9232

Reference: TMS B3LYP/6-311+G(2d,p) GIAO

Reference shielding: 182.466 ppm

^{13}C NMR chemical shifts (ppm), GIAO//6-311+G(2d,p)

Atom	Atom	In the gas phase			In chloroform			In benzene		
label	Assignment	B3LYP	mPW1PW91	wB97XD	B3LYP	mPW1PW91	wB97XD	B3LYP	mPW1PW91	wB97XD
1-C	1	55.2840	47.1945	46.4581	55.2616	49.8194	48.8198	55.2710	49.8265	48.8293
2-C	6	37.0530	30.7314	29.8699	36.8476	31.1168	30.3464	36.9216	31.1930	30.4199
3-C	5	29.8920	23.8814	23.4227	29.6270	24.3406	23.5001	29.7342	24.4529	23.6119
4-C	4	50.4546	43.3315	42.2500	50.3597	44.5978	43.0502	50.3988	44.6420	43.0946
5-C	7	53.1926	45.0488	44.6391	53.2114	47.6186	46.4483	53.2015	47.6092	46.4406
6-C	2	84.8526	76.7394	75.0194	84.9423	78.8029	77.3891	84.8907	78.7437	77.3263
7-C	3	45.3510	39.1454	38.7392	45.1367	39.4344	38.6529	45.2221	39.5206	38.7360
8-C	9	20.9556	15.9040	15.6413	20.6568	15.9203	15.4002	20.7817	16.0478	15.5259
9-C	8	20.9140	15.9506	15.8103	20.6329	15.8711	15.3573	20.7443	15.9865	15.4719
10-C	10	12.4752	7.6016	8.0942	12.3190	7.8011	7.3846	12.3713	7.8562	7.4339
14-C	11-DM-14	-0.8060	-5.3137	-6.2638	-1.2218	-5.0739	-5.8654	-1.0550	-4.9093	-5.7018
15-C	11-DM-15	-2.2570	-6.6044	-7.7847	-2.5596	-6.4751	-7.1227	-2.4452	-6.3676	-7.0110
16-C	12-ipso	143.2511	136.3373	136.7235	143.6306	137.9360	137.9390	143.5023	137.8242	137.8312
17-C	13-o-17	138.9322	133.6678	134.5331	138.9984	134.5445	134.6144	138.9918	134.5433	134.6232
18-C	14-m-18	131.5507	126.2961	126.6578	131.5895	127.1678	127.5745	131.5768	127.1294	127.5297
19-C	15-p	133.4805	128.4363	128.8068	133.6382	129.4011	129.7428	133.5668	129.3008	129.6320
20-C	14-m-20	131.3324	126.0725	126.3239	131.4424	127.0782	127.4550	131.3839	126.9949	127.3607
21-C	13-o-21	138.8868	133.7565	133.6004	139.1805	134.8045	134.9047	139.0349	134.6620	134.7579

Table S21. Optimized coordinates, energies, and calculated NMR chemical shifts of isobornyl TBDPS **2f**

Optimized coordinates in the gas phase

Calculation Method	RB3LYP
Basis Set	6-311+G(2d,p)
Charge	0
Spin	Singlet
E(RB3LYP)	-1377.56756673 a.u.

Zero-point correction=	0.557811 (Hartree/Particle)
Thermal correction to Energy=	0.586708
Thermal correction to Enthalpy=	0.587652
Thermal correction to Gibbs Free Energy=	0.498340
Sum of electronic and zero-point Energies=	-1377.009756
Sum of electronic and thermal Energies=	-1376.980859
Sum of electronic and thermal Enthalpies=	-1376.979915
Sum of electronic and thermal Free Energies=	-1377.069227

Standard orientation:

Center Number	Atomic Number	Atomic Type	Coordinates (Angstroms) X	Y	Z
1	6	0	-2.720068	0.435666	-0.248724
2	6	0	-3.340163	0.624163	-1.662440
3	6	0	-3.896015	-0.783904	-2.028213
4	6	0	-3.525426	-1.622507	-0.786547
5	6	0	-3.707781	-0.613542	0.384926
6	6	0	-1.431490	-0.407027	-0.517953
7	6	0	-1.998281	-1.824747	-0.819250
8	6	0	-3.283980	-1.163376	1.756719
9	6	0	-5.151502	-0.107360	0.543066
10	6	0	-2.493514	1.737155	0.498900
11	8	0	-0.512416	-0.431434	0.580430
12	1	0	-0.918797	0.006385	-1.390143
13	14	0	1.087136	0.028406	0.567385
14	6	0	2.116128	-1.220909	-0.414158
15	6	0	1.596202	-0.025510	2.415284
16	6	0	1.296144	1.710038	-0.286881

17	6	0	3.401016	-0.905008	-0.880600
18	6	0	4.184870	-1.843862	-1.543595
19	6	0	3.697203	-3.128398	-1.762727
20	6	0	2.425684	-3.465596	-1.311750
21	6	0	1.650203	-2.522395	-0.643766
22	6	0	1.441138	1.763451	-1.682395
23	6	0	1.534675	2.973681	-2.362877
24	6	0	1.495483	4.172436	-1.659748
25	6	0	1.359171	4.149174	-0.275941
26	6	0	1.257533	2.934853	0.395236
27	6	0	3.020958	0.530616	2.605620
28	6	0	0.609189	0.757573	3.304533
29	6	0	1.577460	-1.499385	2.877356
30	1	0	-2.596027	0.982708	-2.377118
31	1	0	-4.132336	1.373951	-1.634567
32	1	0	-4.973922	-0.759705	-2.194844
33	1	0	-3.443921	-1.190855	-2.935054
34	1	0	-4.087943	-2.553679	-0.692936
35	1	0	-1.652791	-2.216431	-1.778058
36	1	0	-1.668012	-2.514996	-0.043436
37	1	0	-3.936517	-1.994530	2.041509
38	1	0	-2.254545	-1.508563	1.790370
39	1	0	-3.392456	-0.392576	2.524597
40	1	0	-5.206668	0.658126	1.321429
41	1	0	-5.576519	0.317555	-0.364867
42	1	0	-5.801573	-0.929650	0.855868
43	1	0	-2.053238	1.564284	1.481650
44	1	0	-1.817955	2.395405	-0.053324
45	1	0	-3.435725	2.274129	0.636671
46	1	0	3.798985	0.093266	-0.734009
47	1	0	5.174419	-1.571701	-1.892815
48	1	0	4.304061	-3.860754	-2.282521
49	1	0	2.037326	-4.463922	-1.478706
50	1	0	0.665300	-2.807177	-0.294433
51	1	0	1.491940	0.843632	-2.253805
52	1	0	1.642955	2.979020	-3.441494
53	1	0	1.572648	5.117192	-2.185283
54	1	0	1.330413	5.077642	0.282823
55	1	0	1.144781	2.954448	1.471376
56	1	0	3.315002	0.449283	3.658571

57	1	0	3.757136	-0.026870	2.020771
58	1	0	3.098255	1.583238	2.323949
59	1	0	-0.410384	0.385354	3.193270
60	1	0	0.599345	1.827555	3.089098
61	1	0	0.893388	0.645116	4.357548
62	1	0	0.586710	-1.945935	2.765828
63	1	0	2.289541	-2.113262	2.322509
64	1	0	1.847528	-1.555580	3.938320

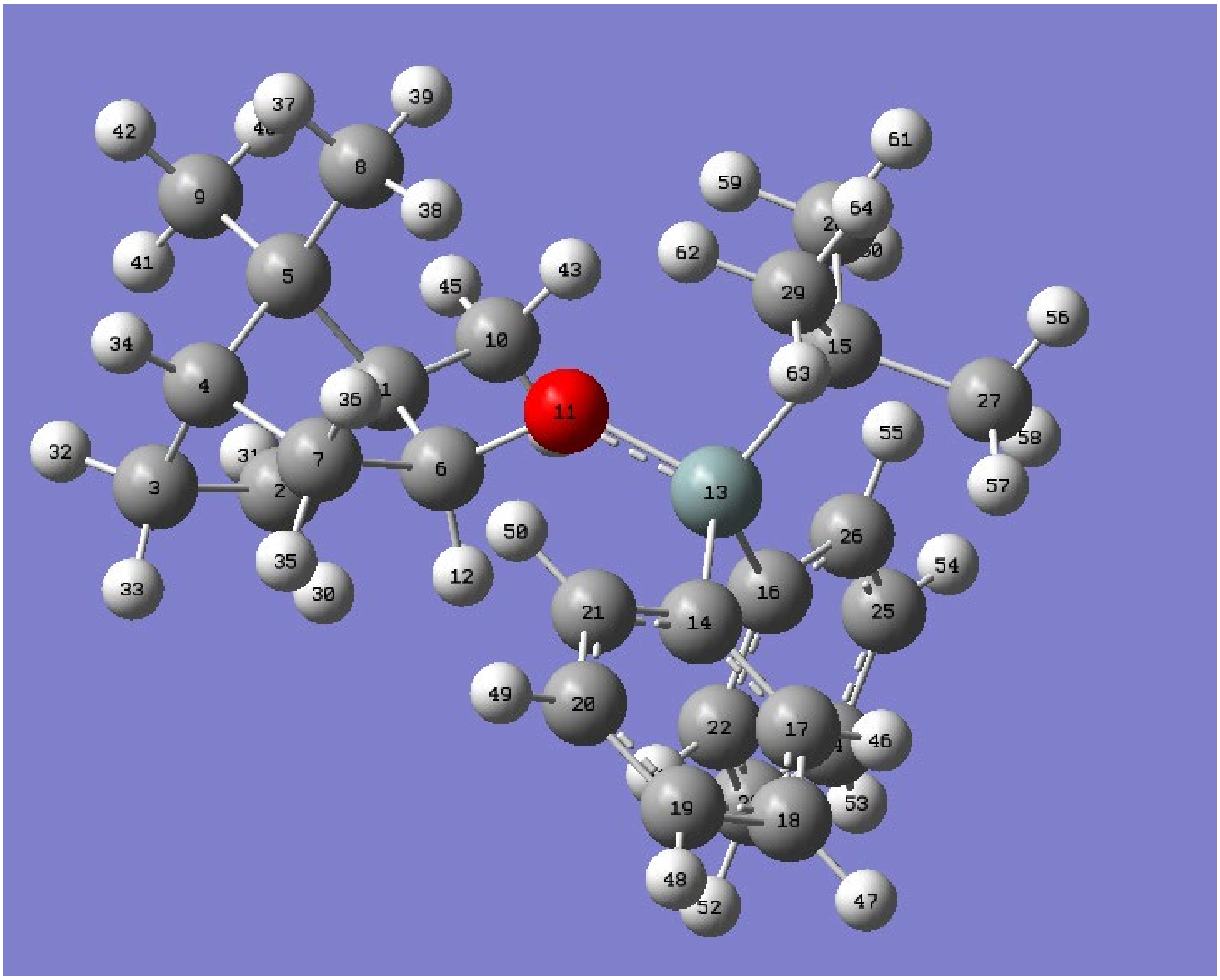

Compound isobornyl TBDPS **2f**

	In the gas phase	In chloroform	In benzene
**Energy**	E(RB3LYP) -1377.56755984 a.u.	E(RB3LYP) -1377.57045458 a.u.	E(RB3LYP) -1377.56930309 a.u.
	E(RmPW1PW91) -1377.30259987 a.u.	(RmPW1PW91) -1377.30594231 a.u.	E(RmPW1PW91) -1377.30461014 a.u.
	E(RwB97XD) -1377.22352061 a.u.	E(RwB97XD) -1377.22688630 a.u.	E(RwB97XD) -1377.22554278 a.u.

Reference: TMS B3LYP/6-311+G(2d,p) GIAO

Reference shielding: 31.8821 ppm

^{1}H NMR chemical shifts (ppm), GIAO//6-311+G(2d,p)

Atom	Atom	In the gas phase			In chloroform			In benzene		
label	Assignment	B3LYP	mPW1PW91	wB97XD	B3LYP	mPW1PW91	wB97XD	B3LYP	mPW1PW91	wB97XD
12-H	2-endo	3.7173	3.8374	3.6690	3.7468	3.8632	3.6940	3.7359	3.8542	3.6854
30-H	6-endo	0.4498	0.5349	0.4357	0.4580	0.5426	0.4421	0.4574	0.5427	0.4428
31-H	6-exo	1.1687	1.2561	1.1608	1.1815	1.2715	1.1770	1.1758	1.2645	1.1696
32-H	5-exo	1.5547	1.6295	1.5277	1.5603	1.6382	1.5375	1.5576	1.6340	1.5328
33-H	5-endo	0.7887	0.8787	0.7862	0.8111	0.9043	0.8132	0.8016	0.8936	0.8019
34-H	4-exo	1.5353	1.6700	1.5963	1.5627	1.6990	1.6269	1.5511	1.6867	1.6139
35-H	3-endo	1.7657	1.8648	1.7761	1.7975	1.8992	1.8116	1.7848	1.8857	1.7975
36-H	3-exo	2.1479	2.2464	2.1273	2.1529	2.2549	2.1327	2.1510	2.2513	2.1305
37-H	9-CH3	0.7450	0.8490	0.8028	0.7534	0.8615	0.8173	0.7485	0.8549	0.8098
38-H	9-CH3	1.9256	1.9990	1.9100	1.8990	1.9707	1.8775	1.9108	1.9832	1.8919
39-H	9-CH3	0.7727	0.8789	0.8064	0.7636	0.8725	0.7985	0.7672	0.8748	0.8015
40-H	8-CH3	0.6522	0.7465	0.6952	0.6453	0.7420	0.6899	0.6482	0.7437	0.6920
41-H	8-CH3	0.7661	0.8424	0.7548	0.7918	0.8687	0.7817	0.7810	0.8576	0.7703
42-H	8-CH3	0.6720	0.7771	0.7593	0.6739	0.7827	0.7660	0.6725	0.7797	0.7626
43-H	10-CH3	0.9670	1.0571	0.9972	0.9579	1.0487	0.9852	0.9592	1.0495	0.9876
44-H	10-CH3	-0.2117	-0.0928	-0.1995	-0.2742	-0.1628	-0.2711	-0.2437	-0.1286	-0.2361
45-H	10-CH3	0.2282	0.3163	0.2730	0.2530	0.3465	0.3058	0.2405	0.3314	0.2896
46-H	14-o-14	7.4653	7.6613	7.6123	7.5186	7.7148	7.6653	7.4999	7.6963	7.6469
47-H	15-m-14	7.4594	7.6652	7.6338	7.5550	7.7719	7.7439	7.5174	7.7299	7.7004
48-H	16-p-14	7.6102	7.8244	7.7890	7.7115	7.9381	7.9062	7.6712	7.8927	7.8594
49-H	15-m-14	7.6389	7.8445	7.7978	7.7326	7.9495	7.9053	7.6948	7.9069	7.8618
50-H	14-o-14	8.1827	8.3988	8.3533	8.2293	8.4470	8.3991	8.2100	8.4269	8.3801
51-H	14-o-16	7.6235	7.8353	7.7625	7.6560	7.8672	7.7948	7.6439	7.8556	7.7831
52-H	15-m-16	7.5005	7.7000	7.6472	7.5955	7.8056	7.7563	7.5584	7.7643	7.7135
53-H	16-p-16	7.6091	7.8194	7.7753	7.7165	7.9389	7.8987	7.6745	7.8920	7.8501
54-H	15-m-16	7.6329	7.8389	7.7770	7.7317	7.9490	7.8895	7.6926	7.9051	7.8446
55-H	14-o-16	8.2930	8.5016	8.4717	8.3465	8.5594	8.5282	8.3230	8.5340	8.5033
56-H	12-CH3-27	0.5137	0.6304	0.6026	0.5308	0.6563	0.6295	0.5215	0.6431	0.6158
57-H	12-CH3-27	0.8575	0.9635	0.8564	0.8407	0.9437	0.8370	0.8503	0.9548	0.8479
58-H	12-CH3-27	1.2232	1.3226	1.2723	1.2381	1.3371	1.2867	1.2314	1.3306	1.2802
59-H	12-CH3-28	1.8230	1.9342	1.8471	1.7828	1.8915	1.8008	1.8002	1.9096	1.8207
60-H	12-CH3-28	1.6542	1.7535	1.6785	1.7074	1.8081	1.7332	1.6852	1.7854	1.7105
61-H	12-CH3-28	0.7874	0.9049	0.8637	0.7977	0.9238	0.8847	0.7915	0.9139	0.8737
62-H	12-CH3-29	1.3235	1.4272	1.3456	1.3109	1.4153	1.3301	1.3156	1.4195	1.3360
63-H	12-CH3-29	0.5184	0.6307	0.5298	0.4752	0.5812	0.4808	0.4966	0.6056	0.5049
64-H	12-CH3-29	0.3264	0.4445	0.3876	0.3514	0.4790	0.4240	0.3383	0.4619	0.4059

Reference: TMS B3LYP/6-311+G(2d,p) GIAO

Reference shielding: 182.466 ppm

^{13}C NMR chemical shifts (ppm), GIAO//6-311+G(2d,p)

Atom	Atom	In the gas phase			In chloroform			In benzene		
label	Assignment	B3LYP	mPW1PW91	wB97XD	B3LYP	mPW1PW91	wB97XD	B3LYP	mPW1PW91	wB97XD
1-C	1	55.5334	50.0569	49.0765	55.5103	50.0293	49.0449	55.5200	50.0410	49.0581
2-C	6	36.7676	31.0957	30.3876	36.5747	30.8943	30.1921	36.6438	30.9656	30.2605
3-C	5	29.8389	24.5670	23.7454	29.5867	24.2999	23.4787	29.6866	24.4046	23.5834
4-C	4	50.5155	44.7874	43.3465	50.4386	44.6968	43.2528	50.4695	44.7328	43.2902
5-C	7	53.1207	47.5564	46.5413	53.1536	47.5912	46.5714	53.1378	47.5746	46.5568
6-C	2	85.1116	78.9932	77.6596	85.0719	78.9668	77.6380	85.0828	78.9714	77.6401
7-C	3	45.5067	39.7678	38.9901	45.3473	39.5959	38.8285	45.4050	39.6581	38.8860
8-C	9	21.0298	16.2527	15.7446	20.7794	15.9940	15.4889	20.8804	16.0978	15.5917
9-C	8	20.8434	16.1022	15.6641	20.5650	15.8139	15.3767	20.6754	15.9281	15.4908
10-C	10	12.3589	7.7509	7.3312	12.2736	7.6672	7.2558	12.2959	7.6877	7.2726
14-C	13-ipso-14	140.9537	135.3265	135.4089	141.3361	135.6750	135.7395	141.1916	135.5477	135.6194
15-C	11	26.5291	20.7472	18.8441	26.4315	20.6554	18.7541	26.4686	20.6895	18.7881
16-C	13-ipso-16	141.4716	135.8874	135.4651	141.5168	135.8936	135.4580	141.4958	135.8916	135.4615
17-C	14-o-14	140.9886	136.3463	136.4481	141.1705	136.5106	136.6058	141.1162	136.4654	136.5634
18-C	15-m-14	130.9900	126.6205	126.8426	131.0731	126.7538	127.0052	131.0338	126.6932	126.9323
19-C	16-p-14	133.2737	129.0335	129.3015	133.4303	129.2511	129.5397	133.3567	129.1506	129.4307
20-C	15-m-14	131.1044	126.7013	126.9516	131.1484	126.7995	127.0691	131.1152	126.7418	127.0036
21-C	14-o-14	141.5628	137.0404	137.1032	141.5888	137.0434	137.0833	141.5763	137.0403	137.0906
22-C	14-o-16	142.7631	138.1525	138.4154	142.8951	138.2620	138.5185	142.8465	138.2249	138.4842
23-C	15-m-16	131.3179	126.9272	127.2138	131.4221	127.0759	127.3820	131.3791	127.0140	127.3122
24-C	16-p-16	133.7651	129.5246	129.7448	134.0363	129.8611	130.1023	133.9271	129.7229	129.9553
25-C	15-m-16	131.6334	127.2224	127.3739	131.7482	127.3944	127.5631	131.6982	127.3179	127.4793
26-C	14-o-16	140.8528	136.2462	136.2679	140.9703	136.3589	136.3640	140.9161	136.3065	136.3187
27-C	12-TB-15	25.4454	20.5810	20.0088	25.1921	20.3142	19.7458	25.2934	20.4208	19.8515
28-C	12-TB-15	27.9130	23.0648	22.3423	27.5883	22.7207	22.0070	27.7228	22.8635	22.1466
29-C	12-TB-15	28.4554	23.4665	22.9069	28.2857	23.2850	22.7395	28.3470	23.3506	22.7992

Table S22. Optimized coordinates, energies, and calculated NMR chemical shifts of isobornyl TPS **2g**

Optimized coordinates in the gas phase

Calculation Method	RB3LYP	
Basis Set	6-311+G(2d,p)	
Charge	0	
Spin	Singlet	
E(RB3LYP)	-1451.38842830	a.u.

Zero-point correction= 0.527202 (Hartree/Particle)
Thermal correction to Energy= 0.555114
Thermal correction to Enthalpy= 0.556058
Thermal correction to Gibbs Free Energy= 0.466278
Sum of electronic and zero-point Energies= -1450.861227
Sum of electronic and thermal Energies= -1450.833314
Sum of electronic and thermal Enthalpies= -1450.832370
Sum of electronic and thermal Free Energies= -1450.922150

Standard orientation:

Center Number	Atomic Number	Atomic Type	Coordinates (Angstroms) X	Y	Z
1	6	0	-2.881912	-0.272073	0.428414
2	6	0	-3.788427	0.754250	1.165785
3	6	0	-4.448273	1.576804	0.019666
4	6	0	-3.835745	0.939008	-1.245171
5	6	0	-3.733830	-0.566819	-0.862594
6	6	0	-1.704983	0.596871	-0.117203
7	6	0	-2.357721	1.373634	-1.296448
8	6	0	-3.036892	-1.437253	-1.920890
9	6	0	-5.094405	-1.226584	-0.581605
10	6	0	-2.459669	-1.450240	1.288493
11	8	0	-0.577819	-0.169037	-0.557977
12	1	0	-1.376011	1.281067	0.669744
13	14	0	0.990754	-0.008964	-0.037865
14	6	0	1.665123	1.699414	-0.463673
15	6	0	1.145815	-0.296818	1.821790
16	6	0	1.916696	-1.337463	-0.989462

17	6	0	2.626595	2.344597	0.326297
18	6	0	3.163824	3.573585	-0.047044
19	6	0	2.746423	4.187749	-1.222501
20	6	0	1.793959	3.565220	-2.024005
21	6	0	1.264617	2.335792	-1.647741
22	6	0	3.311445	-1.287266	-1.116161
23	6	0	4.010013	-2.276782	-1.799600
24	6	0	3.323232	-3.340653	-2.376769
25	6	0	1.938190	-3.406627	-2.267836
26	6	0	1.244281	-2.414553	-1.581043
27	6	0	1.563274	-1.538494	2.320599
28	6	0	1.626553	-1.785935	3.688585
29	6	0	1.270720	-0.791985	4.593687
30	6	0	0.856652	0.450575	4.123537
31	6	0	0.798968	0.692140	2.754940
32	1	0	-3.207562	1.377411	1.849785
33	1	0	-4.534467	0.238382	1.772126
34	1	0	-5.535378	1.486115	0.034320
35	1	0	-4.218010	2.642288	0.081814
36	1	0	-4.386731	1.151385	-2.163488
37	1	0	-2.239808	2.454491	-1.197866
38	1	0	-1.879760	1.074848	-2.228809
39	1	0	-3.646156	-1.476910	-2.829076
40	1	0	-2.043945	-1.088971	-2.191040
41	1	0	-2.934324	-2.463527	-1.557707
42	1	0	-4.957163	-2.256041	-0.240884
43	1	0	-5.693851	-0.711220	0.166997
44	1	0	-5.684643	-1.269988	-1.501603
45	1	0	-1.831509	-2.148274	0.733387
46	1	0	-1.887054	-1.116298	2.157412
47	1	0	-3.331609	-1.995335	1.659598
48	1	0	2.962410	1.886218	1.249629
49	1	0	3.906107	4.051910	0.581773
50	1	0	3.161277	5.145867	-1.513599
51	1	0	1.465810	4.037086	-2.943115
52	1	0	0.528827	1.863069	-2.288491
53	1	0	3.865298	-0.459979	-0.684523
54	1	0	5.088760	-2.215333	-1.886510
55	1	0	3.865094	-4.111629	-2.912404
56	1	0	1.396789	-4.229705	-2.720293

57	1	0	0.164798	-2.471446	-1.510775
58	1	0	1.847122	-2.325854	1.631535
59	1	0	1.954930	-2.754767	4.047243
60	1	0	1.318720	-0.982460	5.659630
61	1	0	0.581983	1.231985	4.822904
62	1	0	0.484911	1.672844	2.413912

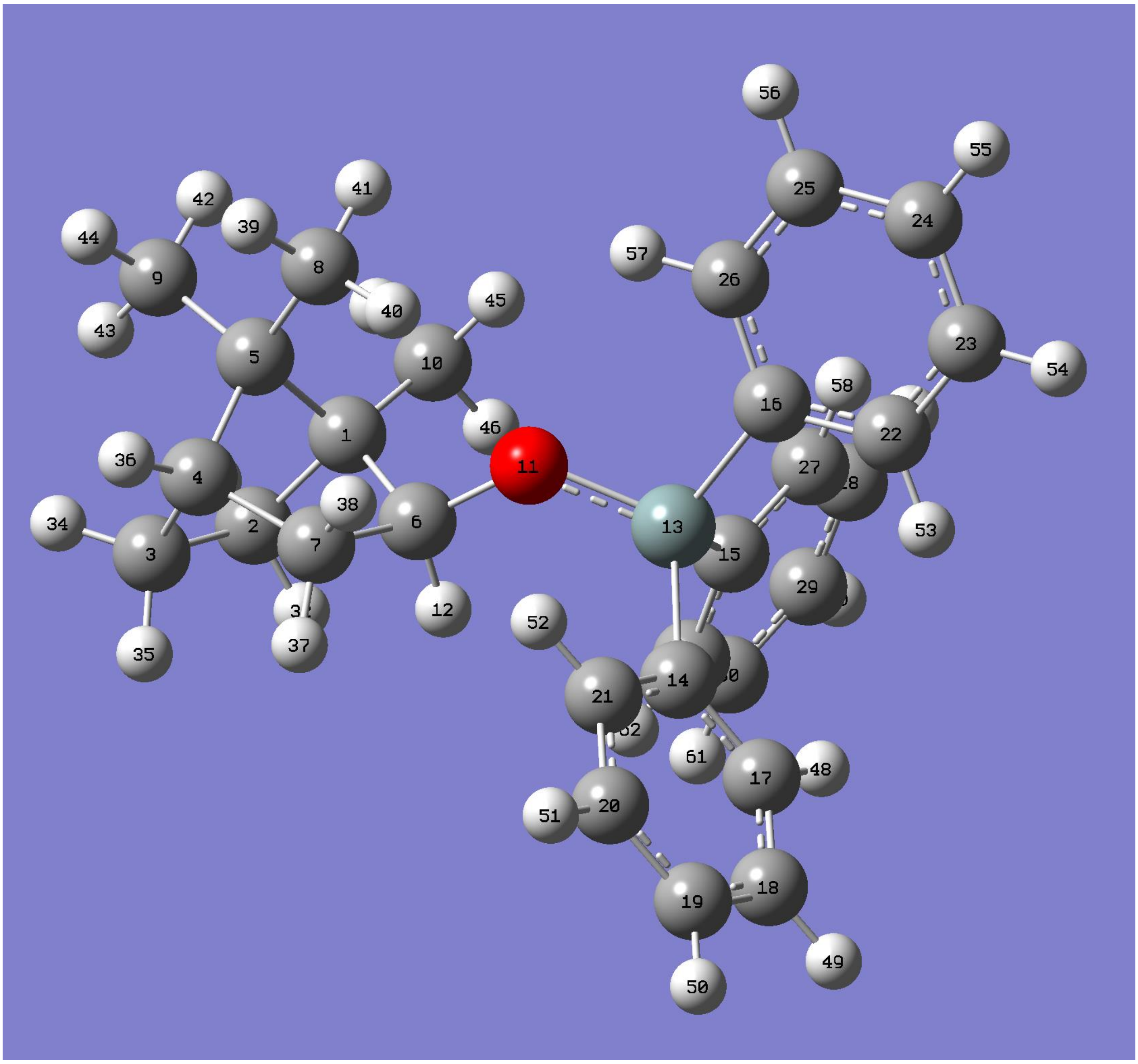

Compound isobornyl TPS **2g**

	In the gas phase	In chloroform	In benzene
**Energy**	E(RB3LYP) -1451.38843553 a.u.	E(RB3LYP) -1451.39269403 a.u.	E(RB3LYP) -1451.39098091 a.u.
	E(RmPW1PW91) -1451.10364753 a.u.	E(RmPW1PW91) -1451.10854341 a.u.	E(RmPW1PW91) -1451.10657274 a.u.
	E(RwB97XD) -1451.00150179 a.u.	E(RwB97XD) -1451.00643840 a.u.	E(RwB97XD) -1451.00444885 a.u.

Reference: TMS B3LYP/6-311+G(2d,p) GIAO

Reference shielding: 31.8821 ppm

^{1}H NMR chemical shifts (ppm), GIAO//6-311+G(2d,p)

Atom	Atom	In the gas phase			In chloroform			In benzene		
label	Assignment	B3LYP	mPW1PW91	wB97XD	B3LYP	mPW1PW91	wB97XD	B3LYP	mPW1PW91	wB97XD
12-H	2-endo	4.0076	4.1250	3.9531	4.0498	4.1647	3.9920	4.0321	4.1482	3.9759
32-H	6-endo	0.6169	0.7038	0.6118	0.6331	0.7203	0.6269	0.6273	0.7147	0.6220
33-H	6-exo	1.2677	1.3575	1.2665	1.2837	1.3764	1.2859	1.2768	1.3681	1.2773
34-H	5-exo	1.5957	1.6724	1.5969	1.6038	1.6836	1.6091	1.6000	1.6784	1.6034
35-H	H-5endo	0.8400	0.9337	0.8479	0.8626	0.9595	0.8749	0.8527	0.9484	0.8633
36-H	4-exo	1.5735	1.7083	1.6329	1.6042	1.7408	1.6668	1.5914	1.7272	1.6525
37-H	3-endo	1.7013	1.8055	1.7138	1.7267	1.8329	1.7421	1.7167	1.8223	1.7311
38-H	3-exo	2.1615	2.2679	2.1521	2.1672	2.2775	2.1590	2.1662	2.2747	2.1574
39-H	9-CH3	0.8499	0.9544	0.8895	0.8630	0.9716	0.9087	0.8564	0.9633	0.8995
40-H	9-CH3	2.1086	2.1895	2.1105	2.0641	2.1427	2.0606	2.0853	2.1650	2.0843
41-H	9-CH3	0.9656	1.0745	0.9824	0.9758	1.0881	0.9947	0.9698	1.0804	0.9876
42-H	8-CH3	0.7350	0.8317	0.7706	0.7305	0.8297	0.7681	0.7321	0.8302	0.7687
43-H	8-CH3	0.8216	0.8988	0.8259	0.8485	0.9264	0.8539	0.8369	0.9144	0.8417
44-H	8-CH3	0.7435	0.8477	0.8066	0.7488	0.8569	0.8167	0.7462	0.8526	0.8120
45-H	10-CH3	1.0192	1.1147	1.0360	1.0093	1.1059	1.0236	1.0115	1.1075	1.0268
46-H	10-CH3	-0.2586	-0.1376	-0.2206	-0.3307	-0.2174	-0.3021	-0.2959	-0.1788	-0.2627
47-H	10-CH3	0.2937	0.3858	0.3323	0.3266	0.4243	0.3733	0.3107	0.4057	0.3535
48-H	15-o-14	7.8247	8.0296	7.9656	7.8838	8.0903	8.0270	7.8606	8.0666	8.0030
49-H	16-m-14	7.5589	7.7673	7.7232	7.6662	7.8868	7.8464	7.6222	7.8378	7.7958
50-H	17-p-14	7.6185	7.8347	7.7783	7.7323	7.9619	7.9095	7.6862	7.9102	7.8561
51-H	16-m-14	7.5891	7.7923	7.7501	7.6912	7.9066	7.8668	7.6503	7.8606	7.8199
52-H	15-o-14	8.1615	8.3791	8.3192	8.2112	8.4316	8.3703	8.1938	8.4131	8.3525
53-H	15-o-16	7.4103	7.6156	7.5465	7.4753	7.6816	7.6131	7.4509	7.6571	7.5883
54-H	16-m-16	7.3920	7.5940	7.5406	7.5005	7.7147	7.6648	7.4563	7.6657	7.6142
55-H	17-p-16	7.5587	7.7710	7.7286	7.6745	7.9007	7.8604	7.6265	7.8469	7.8056
56-H	16-m-16	7.6561	7.8560	7.7996	7.7611	7.9737	7.9193	7.7173	7.9246	7.8693
57-H	15-o-16	8.4418	8.6558	8.6344	8.4840	8.7001	8.6762	8.4659	8.6810	8.6582
58-H	15-o-15	7.4229	7.6367	7.5564	7.4113	7.6244	7.5421	7.4191	7.6327	7.5512
59-H	16-m-15	7.4636	7.6618	7.6290	7.5519	7.7614	7.7320	7.5180	7.7229	7.6921
60-H	17-p-15	7.6083	7.8225	7.7816	7.7221	7.9494	7.9122	7.6767	7.8986	7.8598
61-H	16-m-15	7.5886	7.7914	7.7475	7.7027	7.9176	7.8773	7.6562	7.8661	7.8243
62-H	15-o-15	7.9654	8.1750	8.1179	8.0343	8.2461	8.1893	8.0061	8.2172	8.1603

Reference: TMS B3LYP/6-311+G(2d,p) GIAO

Reference shielding: 182.466 ppm

^{13}C NMR chemical shifts (ppm), GIAO//6-311+G(2d,p)

Atom	Atom	In the gas phase			In chloroform			In benzene		
label	Assignment	B3LYP	mPW1PW91	wB97XD	B3LYP	mPW1PW91	wB97XD	B3LYP	mPW1PW91	wB97XD
1-C	1	56.1436	50.6568	49.6003	56.1760	50.6944	49.6353	56.1624	50.6787	49.6207
2-C	6	29.6393	24.1638	23.3100	29.5180	24.0259	23.1890	29.5669	24.0815	23.2376
3-C	5	31.1579	25.8734	25.0377	30.9252	25.6237	24.7992	31.0188	25.7238	24.8944
4-C	4	50.5120	44.7250	43.0715	50.3909	44.5905	42.9410	50.4419	44.6461	42.9950
5-C	7	53.9359	48.2353	46.9770	53.9962	48.3036	47.0362	53.9699	48.2737	47.0102
6-C	2	83.4463	77.4507	76.0887	83.6139	77.6418	76.2852	83.5320	77.5489	76.1893
7-C	3	40.8608	35.2311	34.6490	40.7544	35.1151	34.5436	40.7951	35.1595	34.5833
8-C	9	19.0570	14.3497	13.8862	18.7665	14.0496	13.5879	18.8855	14.1721	13.7099
9-C	8	20.6010	15.8807	15.4665	20.3117	15.5845	15.1711	20.4264	15.7015	15.2881
10-C	10	14.0648	9.5870	9.1600	13.8319	9.3435	8.9263	13.9224	9.4380	9.0166
14-C	11-ipso-14	142.4758	136.7623	136.6129	142.5887	136.8061	136.6489	142.5532	136.8027	136.6486
15-C	11-ipso-15	141.2426	135.4138	135.4634	141.6474	135.7543	135.8132	141.5149	135.6515	135.7074
16-C	11-ipso-16	139.5934	133.9182	133.7783	139.5691	133.8371	133.6856	139.6007	133.8967	133.7501
17-C	12-o-14	140.1765	135.6252	135.6535	140.4730	135.9150	135.9428	140.3453	135.7904	135.8186
18-C	13-m-14	131.0669	126.6728	126.8910	131.2744	126.9383	127.1818	131.1596	126.7975	127.0296
19-C	14-p-14	133.5565	129.3226	129.5679	133.8796	129.7248	129.9948	133.7308	129.5412	129.8008
20-C	13-m-14	131.9253	127.4595	127.6068	132.0318	127.6276	127.7943	131.9811	127.5498	127.7082
21-C	12-o-14	141.0095	136.4469	136.5858	140.9228	136.3415	136.4575	140.9989	136.4272	136.5546
22-C	12-o-16	142.2516	137.9431	138.0724	141.8678	137.5233	137.6350	142.0439	137.7169	137.8364
23-C	13-m-16	132.4493	128.0301	128.1363	132.5034	128.1410	128.2673	132.4827	128.0947	128.2128
24-C	14-p-16	134.0701	129.8500	130.1029	134.4614	130.3153	130.5904	134.2889	130.1091	130.3748
25-C	13-m-16	130.6966	126.2866	126.5294	131.0692	126.7197	126.9833	130.8964	126.5195	126.7745
26-C	12-o-16	140.4181	135.7936	136.0970	140.8207	136.1903	136.4809	140.6487	136.0212	136.3176
27-C	12-o-15	140.0214	135.6039	135.6481	139.9718	135.5139	135.5508	140.0238	135.5865	135.6267
28-C	13-m-15	131.4836	127.0448	127.1453	131.7307	127.3518	127.4802	131.6106	127.2061	127.3218
29-C	14-p-15	133.4490	129.2184	129.4871	133.7630	129.6141	129.8941	133.6026	129.4175	129.6919
30-C	13-m-15	131.2854	126.8739	127.1031	131.5056	127.1647	127.4161	131.3837	127.0105	127.2521
31-C	12-o-15	139.8403	135.4237	135.7030	139.8366	135.4210	135.6752	139.8431	135.4265	135.6928

Table S23 Comparison of the coefficient of determination (r2) and RMS between experimental and calculated ^{1}H NMR chemical shifts for compound 1d-1g and 2d-2g using various calculation methods.

Compound	Method/Basis set	$CDCl_3$				C_6D_6				CD_3OD			
		in the gas phase		in $CHCl_3$		in the gas phase		in C_6H_6		in the gas phase		in CH_3OH	
		r^2	RMS	r^2	RMS	r^2	RMS	r^2	RMS	r^2	RMS	r^2	RMS
Borneol **1a**	B3LYP/6-311+G(2d,p)	0.9814	0.141	0.9930	0.111	0.9930	0.135	0.9938	0.141	0.9885	0.122	0.9982	0.110
	ωB97XD/6-311+G(2d,p)	0.9803	0.135	0.9930	0.094	0.9928	0.111	0.9939	0.114	0.9875	0.111	0.9984	0.083
	***m*PW1PW91/6-311+G(2d,p)**	0.9820	0.143	0.9938	0.114	0.9933	0.185	0.9940	0.190	0.9889	0.131	0.9988	0.120
Bornyl acetate **1b**	B3LYP/6-311+G(2d,p)	0.9950	0.101	0.9964	0.081	0.9916	0.114	0.9911	0.119	0.9958	0.111	0.9975	0.081
	ωB97XD/6-311+G(2d,p)	0.9966	0.083	0.9977	0.061	0.9926	0.115	0.9920	0.122	0.9970	0.094	0.9985	0.065
	***m*PW1PW91/6-311+G(2d,p)**	0.9964	0.100	0.9975	0.094	0.9929	0.133	0.9922	0.143	0.9971	0.088	0.9983	0.074
Bornyl benzoate **1c**	B3LYP/6-311+G(2d,p)	0.9944	0.103	0.9952	0.090	0.9924	0.124	0.9920	0.131	0.9954	0.105	0.9965	0.082
	ωB97XD/6-311+G(2d,p)	0.9908	0.129	0.9921	0.116	0.9893	0.164	0.9892	0.168	0.9908	0.131	0.9935	0.113
	***m*PW1PW91/6-311+G(2d,p)**	0.9930	0.107	0.9940	0.101	0.9906	0.164	0.9903	0.173	0.9940	0.100	0.9953	0.089
Isoborneol **2a**	B3LYP/6-311+G(2d,p)	0.9888	0.109	0.9934	0.092	0.9904	0.125	0.9875	0.138	0.9915	0.100	0.9959	0.089
	ωB97XD/6-311+G(2d,p)	0.9906	0.096	0.9949	0.075	0.9929	0.102	0.9895	0.118	0.9922	0.088	0.9960	0.072
	***m*PW1PW91/6-311+G(2d,p)**	0.9904	0.086	0.9952	0.081	0.9929	0.168	0.9901	0.182	0.9923	0.088	0.9972	0.094
Isobornyl acetate **2b**	B3LYP/6-311+G(2d,p)	0.9912	0.203	0.9943	0.176	0.9881	0.175	0.9886	0.170	0.9917	0.213	0.9962	0.173
	ωB97XD/6-311+G(2d,p)	0.9907	0.167	0.9943	0.137	0.9893	0.134	0.9901	0.130	0.9909	0.182	0.9961	0.137
	***m*PW1PW91/6-311+G(2d,p)**	0.9905	0.209	0.9936	0.187	0.9877	0.207	0.9883	0.205	0.9910	0.211	0.9932	0.176
Isobornyl benzoate **2c**	B3LYP/6-311+G(2d,p)	0.9914	0.218	0.9936	0.751	0.9874	0.214	0.9877	0.215	0.9937	0.218	0.9937	0.218
	ωB97XD/6-311+G(2d,p)	0.9911	0.183	0.9934	0.160	0.9884	0.180	0.9887	0.183	0.9932	0.182	0.9960	0.150
	***m*PW1PW91/6-311+G(2d,p)**	0.9907	0.231	0.9929	0.215	0.9873	0.260	0.9876	0.264	0.9931	0.230	0.9958	0.209

Table S24 Comparison of the coefficient of determination (r2) and RMS between experimental and calculated ^{1}H NMR chemical shifts for compounds 1d–1g and 2d–2g using various calculation methods.

Compound	Method/Basis set	$CDCl_3$				C_6D_6			
		in the gas phase		in $CHCl_3$		in the gas phase		in C_6H_6	
		r^2	RMS	r^2	RMS	r^2	RMS	r^2	RMS
Bornyl TBDMS **1d**	B3LYP/6-311+G(2d,p)	0.9963	0.074	0.9977	0.076	0.9960	0.094	0.9940	0.103
	*ω*B97XD/6-311+G(2d,p)	0.9952	0.068	0.9981	0.055	0.9952	0.106	0.9939	0.092
	***m*PW1PW91/6-311+G(2d,p)**	0.9956	0.090	0.9986	0.120	0.9953	0.071	0.9944	0.088
Bornyl DMMPS **1e**	B3LYP/6-311+G(2d,p)	0.9855	0.133	0.9893	0.119	0.9941	0.146	0.9924	0.150
	*ω*B97XD/6-311+G(2d,p)	0.9792	0.173	0.9895	0.121	0.9914	0.189	0.9923	0.156
	***m*PW1PW91/6-311+G(2d,p)**	0.9844	0.116	0.9908	0.089	0.9936	0.110	0.9935	0.083
Bornyl TBDPS **1f**	B3LYP/6-311+G(2d,p)	0.9646	0.182	0.9684	0.175	0.9830	0.181	0.9836	0.180
	*ω*B97XD/6-311+G(2d,p)	0.9403	0.244	0.9679	0.188	0.9685	0.245	0.9829	0.199
	***m*PW1PW91/6-311+G(2d,p)**	0.9621	0.193	0.9705	0.195	0.9825	0.171	0.9850	0.158
Bornyl TPS **1g**	B3LYP/6-311+G(2d,p)	0.9834	0.126	0.9871	0.114	0.9946	0.111	0.9945	0.112
	*ω*B97XD/6-311+G(2d,p)	0.9846	0.126	0.9880	0.117	0.9949	0.123	0.9944	0.127
	***m*PW1PW91/6-311+G(2d,p)**	0.9852	0.166	0.9887	0.162	0.9957	0.110	0.9954	0.115
Isobornyl TBDMS 2**d**	B3LYP/6-311+G(2d,p)	0.9939	0.071	0.9960	0.067	0.9924	0.085	0.9917	0.089
	*ω*B97XD/6-311+G(2d,p)	0.9910	0.081	0.9965	0.051	0.9957	0.068	0.9933	0.070
	***m*PW1PW91/6-311+G(2d,p)**	0.9917	0.077	0.9975	0.104	0.9924	0.076	0.9937	0.108
Isobornyl DMMPS **2e**	B3LYP/6-311+G(2d,p)	0.9792	0.160	0.9808	0.149	0.9901	0.132	0.9891	0.129
	*ω*B97XD/6-311+G(2d,p)	0.9739	0.194	0.9757	0.158	0.9854	0.171	0.9876	0.133
	***m*PW1PW91/6-311+G(2d,p)**	0.9746	0.139	0.9803	0.106	0.9891	0.101	0.9900	0.076
Isobornyl TBDPS 2**f**	B3LYP/6-311+G(2d,p)	0.9416	0.237	0.9403	0.243	0.9355	0.240	0.9410	0.244
	*ω*B97XD/6-311+G(2d,p)	0.9395	0.231	0.9372	0.237	0.9347	0.234	0.9321	0.239
	***m*PW1PW91/6-311+G(2d,p)**	0.9431	0.246	0.9409	0.258	0.9373	0.242	0.9348	0.250
Isobornyl TPS 2**g**	B3LYP/6-311+G(2d,p)	0.9686	0.196	0.9686	0.202	0.9581	0.202	0.9569	0.207
	*ω*B97XD/6-311+G(2d,p)	0.9676	0.186	0.9672	0.192	0.9570	0.196	0.9553	0.202
	***m*PW1PW91/6-311+G(2d,p)**	0.9693	0.215	0.9688	0.228	0.9593	0.221	0.9577	0.230

Table S25 Comparison of the coefficient of determination (r2) and RMS between experimental and calculated ^{13}C NMR chemical shifts for compound 1d-1g and 2d-2g using various calculation methods.

		**$CDCl_3$**				**C_6D_6**				**CD_3OD**			
**Compound**	**Method/Basis set**	in the gas phase		in $CHCl_3$		in the gas phase		in C_6H_6		in the gas phase		in CH_3OH	
		r^2	RMS	r^2	RMS	r^2	RMS	r^2	RMS	r^2	RMS	r^2	RMS
Borneol **1a**	B3LYP/6-311+G(2d,p)	0.9949	3.809	0.9945	3.785	0.9951	3.783	0.9950	3.769	0.9967	3.168	0.9962	3.153
	ωB97XD/6-311+G(2d,p)	0.9955	3.473	0.9952	3.607	0.9953	3.645	0.9953	3.726	0.9967	4.043	0.9964	4.223
	***m*PW1PW91/6-311+G(2d,p)**	0.9946	2.927	0.9943	3.070	0.9947	3.095	0.9946	3.181	0.9963	3.383	0.9958	3.581
Bornyl acetate **1b**	B3LYP/6-311+G(2d,p)	0.9957	3.686	0.9956	3.698	0.9966	3.543	0.9965	3.557	0.9964	2.794	0.9962	2.831
	ωB97XD/6-311+G(2d,p)	0.9972	3.157	0.9970	3.312	0.9977	2.720	0.9975	3.399	0.9975	3.890	0.9972	4.089
	***m*PW1PW91/6-311+G(2d,p)**	0.9963	2.596	0.9961	2.770	0.9969	3.305	0.9968	2.827	0.9968	3.198	0.9966	3.423
Bornyl benzoate **1c**	B3LYP/6-311+G(2d,p)	0.9879	4.097	0.9859	4.141	0.9869	3.975	0.9868	4.005	0.9879	3.279	0.9875	3.363
	ωB97XD/6-311+G(2d,p)	0.9908	4.129	0.9884	4.245	0.9893	4.227	0.9891	4.297	0.9908	4.350	0.9897	4.511
	***m*PW1PW91/6-311+G(2d,p)**	0.9901	3.641	0.9898	3.766	0.9907	3.733	0.9905	3.808	0.9916	4.875	0.9910	5.027
Isoborneol **2a**	B3LYP/6-311+G(2d,p)	0.9962	3.893	0.9956	3.828	0.9961	3.804	0.9959	3.769	0.9971	3.221	0.9964	3.164
	ωB97XD/6-311+G(2d,p)	0.9961	3.383	0.9956	3.589	0.9959	3.617	0.9954	3.738	0.9966	4.090	0.9958	4.382
	***m*PW1PW91/6-311+G(2d,p)**	0.9959	2.832	0.9952	3.039	0.9957	3.054	0.9956	3.176	0.9966	3.430	0.9958	3.731
Isobornyl acetate **2b**	B3LYP/6-311+G(2d,p)	0.9901	3.576	0.9904	3.603	0.9909	3.452	0.9911	3.472	0.9907	2.764	0.9911	2.816
	ωB97XD/6-311+G(2d,p)	0.9945	4.154	0.9945	4.222	0.9950	4.280	0.9950	4.327	0.9948	5.044	0.9950	5.122
	***m*PW1PW91/6-311+G(2d,p)**	0.9938	3.735	0.9940	3.799	0.9945	3.850	0.9946	3.895	0.9944	4.589	0.9946	4.662
Isobornyl benzoate **2c**	B3LYP/6-311+G(2d,p)	0.9894	3.563	0.9899	3.600	0.9901	3.464	0.9903	3.488	0.9904	2.775	0.9909	2.839
	ωB97XD/6-311+G(2d,p)	0.9935	4.310	0.9937	4.354	0.9940	4.393	0.9942	4.441	0.9940	5.165	0.9942	5.214
	***m*PW1PW91/6-311+G(2d,p)**	0.9933	3.774	0.9936	3.820	0.9938	3.850	0.9940	3.882	0.9940	4.590	0.9944	4.639

Table S26 Comparison of the coefficient of determination (r2) and RMS between experimental and calculated ^{13}C NMR chemical shifts for compounds 1d–1g and 2d–2g using various calculation methods.

Compound	Method/Basis set	$CDCl_3$				C_6D_6			
		in the gas phase		in $CHCl_3$		in the gas phase		in C_6H_6	
		r^2	RMS	r^2	RMS	r^2	RMS	r^2	RMS
Bornyl TBDMS **1d**	B3LYP/6-311+G(2d,p)	0.9942	3.753	0.9937	3.699	0.9941	3.486	0.9938	3.456
	ωB97XD/6-311+G(2d,p)	0.9975	3.832	0.9952	3.484	0.9973	4.127	0.9952	3.683
	***m*PW1PW91/6-311+G(2d,p)**	0.9974	3.339	0.9943	2.901	0.9973	3.617	0.9943	3.053
Bornyl DMMPS **1e**	B3LYP/6-311+G(2d,p)	0.9943	3.747	0.9941	3.716	0.9944	3.478	0.9943	3.459
	ωB97XD/6-311+G(2d,p)	0.9973	3.739	0.9956	3.416	0.9975	4.018	0.9956	3.610
	***m*PW1PW91/6-311+G(2d,p)**	0.9973	3.271	0.9947	2.853	0.9975	3.536	0.9948	2.997
Bornyl TBDPS **1f**	B3LYP/6-311+G(2d,p)	0.9942	3.832	0.9940	3.809	0.9940	3.570	0.9939	3.558
	ωB97XD/6-311+G(2d,p)	0.9963	4.014	0.9953	3.523	0.9961	4.315	0.9952	3.728
	***m*PW1PW91/6-311+G(2d,p)**	0.9964	3.466	0.9943	3.048	0.9962	3.749	0.9941	3.201
Bornyl TPS **1g**	B3LYP/6-311+G(2d,p)	0.9945	3.708	0.9960	3.694	0.9945	3.460	0.9944	3.451
	ωB97XD/6-311+G(2d,p)	0.9960	3.313	0.9958	3.460	0.9958	3.553	0.9957	3.640
	***m*PW1PW91/6-311+G(2d,p)**	0.9949	2.790	0.9948	2.952	0.9948	2.985	0.9947	3.081
Isobornyl TBDMS 2**d**	B3LYP/6-311+G(2d,p)	0.9955	3.984	0.9949	3.885	0.9954	3.729	0.9951	3.675
	ωB97XD/6-311+G(2d,p)	0.9981	3.552	0.9955	3.241	0.9981	3.843	0.9957	3.414
	***m*PW1PW91/6-311+G(2d,p)**	0.9982	3.035	0.9975	2.731	0.9982	3.310	0.9952	2.861
Isobornyl DMMPS **2e**	B3LYP/6-311+G(2d,p)	0.9953	3.847	0.9951	3.791	0.9952	3.603	0.9951	3.570
	ωB97XD/6-311+G(2d,p)	0.9977	3.613	0.9957	3.309	0.9976	3.894	0.9957	3.500
	***m*PW1PW91/6-311+G(2d,p)**	0.9982	3.134	0.9953	2.761	0.9981	3.406	0.9953	2.909
Isobornyl TBDPS 2**f**	B3LYP/6-311+G(2d,p)	0.9951	3.776	0.9948	3.716	0.9950	3.520	0.9949	3.486
	ωB97XD/6-311+G(2d,p)	0.9958	3.213	0.9955	3.363	0.9957	3.486	0.9956	3.579
	***m*PW1PW91/6-311+G(2d,p)**	0.9953	2.693	0.9950	2.844	0.9953	2.927	0.9951	3.022
Isobornyl TPS 2**g**	B3LYP/6-311+G(2d,p)	0.9936	3.828	0.9932	3.772	0.9935	3.583	0.9933	3.551
	ωB97XD/6-311+G(2d,p)	0.9952	3.208	0.9948	3.366	0.9951	3.469	0.9949	3.566
	***m*PW1PW91/6-311+G(2d,p)**	0.9941	2.680	0.9936	2.841	0.9940	2.899	0.9937	2.998
